# Reference ranges (“normal values”) for cardiovascular magnetic resonance (CMR) in adults and children: 2020 update

**DOI:** 10.1186/s12968-020-00683-3

**Published:** 2020-12-14

**Authors:** Nadine Kawel-Boehm, Scott J. Hetzel, Bharath Ambale-Venkatesh, Gabriella Captur, Christopher J. Francois, Michael Jerosch-Herold, Michael Salerno, Shawn D. Teague, Emanuela Valsangiacomo-Buechel, Rob J. van der Geest, David A. Bluemke

**Affiliations:** 1 Department of Radiology, Kantonsspital Graubuenden, Loestrasse 170, 7000 Chur, Switzerland; 2 Institute for Diagnostic, Interventional and Pediatric Radiology (DIPR), Bern University Hospital, University of Bern, Freiburgstrasse 10, 3010 InselspitalBern, Switzerland; 3 Department of Biostatistics and Medical Informatics, University of Wisconsin, 610 Walnut St, Madison, WI 53726 USA; 4 Department of Radiology, Johns Hopkins University, 600 N Wolfe Street, Baltimore, MD 21287 USA; 5 MRC Unit of Lifelong Health and Ageing At UCL, 5-19 Torrington Place, Fitzrovia, London, WC1E 7HB UK; 6 Inherited Heart Muscle Conditions Clinic, Royal Free Hospital NHS Foundation Trust, Hampstead, London, NW3 2QG UK; 7 Department of Radiology, University of Wisconsin School of Medicine and Public Health, 600 Highland Avenue, Madison, WI 53792 USA; 8 Department of Radiology, Brigham and Women’s Hospital, 75 Francis Street, Boston, MA 02115 USA; 9 Cardiovascular Division, University of Virginia Health System, 1215 Lee Street, Charlottesville, VA 22908 USA; 10 Department of Radiology, National Jewish Health, 1400 Jackson St, Denver, CO 80206 USA; 11 Division of Paediatric Cardiology, University Children’s Hospital Zurich, Steinwiesstrasse 75, 8032 Zurich, Switzerland; 12 Department of Radiology, Leiden University Medical Center, Albinusdreef 2, 2333ZA Leiden, The Netherlands

**Keywords:** Normal values, Reference range, Cardiac magnetic resonance

## Abstract

Cardiovascular magnetic resonance (CMR) enables assessment and quantification of morphological and functional parameters of the heart, including chamber size and function, diameters of the aorta and pulmonary arteries, flow and myocardial relaxation times. Knowledge of reference ranges (“normal values”) for quantitative CMR is crucial to interpretation of results and to distinguish normal from disease. Compared to the previous version of this review published in 2015, we present updated and expanded reference values for morphological and functional CMR parameters of the cardiovascular system based on the peer-reviewed literature and current CMR techniques. Further, databases and references for deep learning methods are included.

## Background

Cardiovascular magnetic resonance (CMR) provides a wealth of information to help distinguish health from disease. In addition to non-invasively defining chamber sizes and global function, CMR can also assess regional cardiac function as well as tissue composition (myocardial T1, T2 and T2* relaxation time). Advantages of quantitative evaluation of CMR images are objective differentiation between pathology and normal conditions, grading of disease severity, monitoring changes during therapy and evaluating prognosis [1].

Knowledge of the range of normal structure and function is required to interpret abnormal cardiac conditions. Thus, the aim of this review is to provide reference intervals (“normal values”) for morphological and functional CMR parameters of the cardiovascular system based on a systematic review of the literature using current CMR techniques and sequences.

Since the initial publication of the “normal value review” in 2015 [1], new research related to CMR reference values have been published and are now integrated in this update. Previous topics were expanded with new sections including morphological and functional parameters in athletes, myocardial T2 mapping, myocardial perfusion, left-ventricular (LV) trabeculation and normal dimensions of the pulmonary arteries in adults and children. Further, feature tracking is increasingly used to assess myocardial strain and reference intervals are now available for that technology. Deep learning methods are rapidly being incorporated into clinical software analysis packages [2, 3]. These new analytic methods are expected to accelerate quantification of myocardial function from CMR images. To date, reference ranges based on cohorts of healthy subjects using deep learning methods have not been presented. However due to the potential importance of this topic, we present algorithms and major references related to CMR on these methods.

## Methods

A literature search was performed in PubMed to identify publications of CMR reference intervals for each section. When feasible (discussed further below), we sought to provide weighted means calculated based on these published normal values in healthy individuals. General criteria used for inclusion of data in this review are as follows:

- Sample size of at least 40 subjects. 40 subjects is accepted as the smallest sample size that allows calculation of reference ranges using a parametric method for data with a Gaussian distribution [4]. In some circumstances, separate reference ranges need to be provided by gender. In that case, the sample size of included studies were at least a minimum of 40 subjects per gender. Exceptions to sample size of 40 subjects per group were made for clinically relevant parameters where no publication was available with sufficient sample size for certain parameters. However, reference ranges based on a smaller sample size are of limited validity and should be applied with caution.
- Only values of “healthy” reference cohorts were included. In particular, reference cohorts that included subjects with a disease or condition known to affect the measured parameter (e.g. hypertension and diabetes) were excluded. For publications that described population statistics (e.g., the MESA study, UK Biobank), we used data only from subgroups of individuals without risk factors or conditions known to affect the CMR parameter. In cases where the original manuscript did not provide sufficient information to allow upper and lower limits to be calculated, authors were contacted for clarification.
- If two or more publications were determined to refer to the same healthy reference cohort, the values of the cohort were included only once.

Manuscripts were then excluded from consideration as follows: (a) obsolescent CMR technique, (b) missing data that were not provided by the authors of the original publication on request and/ or (c) insufficient or inconsistent description of methods and/or (d) methods of analysis that were not consistent with current Society for Cardiovascular Magnetic Resonance (SCMR) guidelines [5] as of the time of this review.

Technical factors such as sequence parameters are relevant for CMR, and these factors are provided in relationship to the reference values. In addition, factors related to post processing will affect the CMR analysis and these factors are also described. Finally, when available, the relationship of demographic factors (e.g. age, gender, and ethnicity) to reference values are described in each section.

### Statistical methods

Statistical analyses were performed with R for statistical computing (version 3.5, R Core Team, Vienna, Austria). Results from multiple studies reporting normal values for the same CMR parameters were combined using a random effects meta-analysis model as implemented by the metamean function in the meta library in R. This produced a weighted, pooled estimate of the population mean of the CMR parameters in the combined studies. Upper and lower limits of normal values were calculated as ± 2SDp, where SDp is the pooled standard deviation calculated from the standard deviations reported in each study. Mean values and limits of normal values were “rounded up” to avoid excess digits beyond the measurement capability of CMR.

## Left ventricular dimensions and functions in the adult

### CMR acquisition parameters

The primary method used to assess the LV is balanced steady-state free precession (bSSFP) technique at 1.5 or 3 T CMR (Table 1). bSSFP technique yields improved blood-myocardial contrast compared to its predecessor, fast gradient echo (FGRE) sequence.

**Table 1:** References, normal adult left ventricular volumes, function and dimensions

First author, year	CMR technique	n, male:female	Age range (years)
Hudsmith, 2005 [22]	1.5 T, short axis bSSFP, papillary muscles included in LV mass	63:45	21–68
Maceira, 2006 [10]	1.5 T, short axis bSSFP, papillary muscles included in LV mass	60:60	20–80
Chang, 2012 [23]	1.5 T, short axis bSSFP, papillary muscles included in LV volume	64:60	20–70
Macedo, 2013 [24]	1.5 T, short axis bSSFP, papillary muscles included in LV mass	54:53	20–80
Yeon, 2015 [25]	1.5 T, short axis bSSFP, papillary muscles included in LV volume	512:340	(61 ± 9)^a^
Le, 2016 [11]	3 T, short axis bSSFP, papillary muscles included in LV mass	91:89	20–69
Le Ven, 2016 [14]	1.5 T, Short axis bSSFP, papillary muscles included in LV mass	196:238	18–36
Lei, 2017 [15]	3 T, short axis bSSFP, papillary muscles included in LV volume	60:60	23–83
Petersen, 2017 [16]	1.5 T, short axis bSSFP, papillary muscles included in LV volume	368:432	45–74
Bentatou, 2018 [12]	1.5 T, short axis bSSFP, papillary muscles included in LV mass	70:70	20–69
Buelow, 2018 [13]	1.5 T, short axis bSSFP, papillary muscles included in LV mass	291:326	20–80^b^
Liu, 2018 [26]	1.5 T, short axis bSSFP, papillary muscles included in LV mass	50:50	20–70

*n* number of study subjects, *bSSFP* balanced steady-state free precession, *LV* left ventricle

^a^Mean ± SD (age-range not provided in original publication)

^b^6 subjects > 80 years included

### CMR analysis methods

Papillary muscle mass has been shown to significantly affect LV volumes and mass [6–8]. No uniformly accepted convention has been used for analyzing trabeculation and papillary muscle mass. Post-processing recommendations by the SCMR [9] stipulate that papillary muscles should either be consistently included in the LV volume or in the LV mass, but not in both. Tables of normal values should specify the status of the papillary muscles in the CMR analysis.

**Fig. 1:**
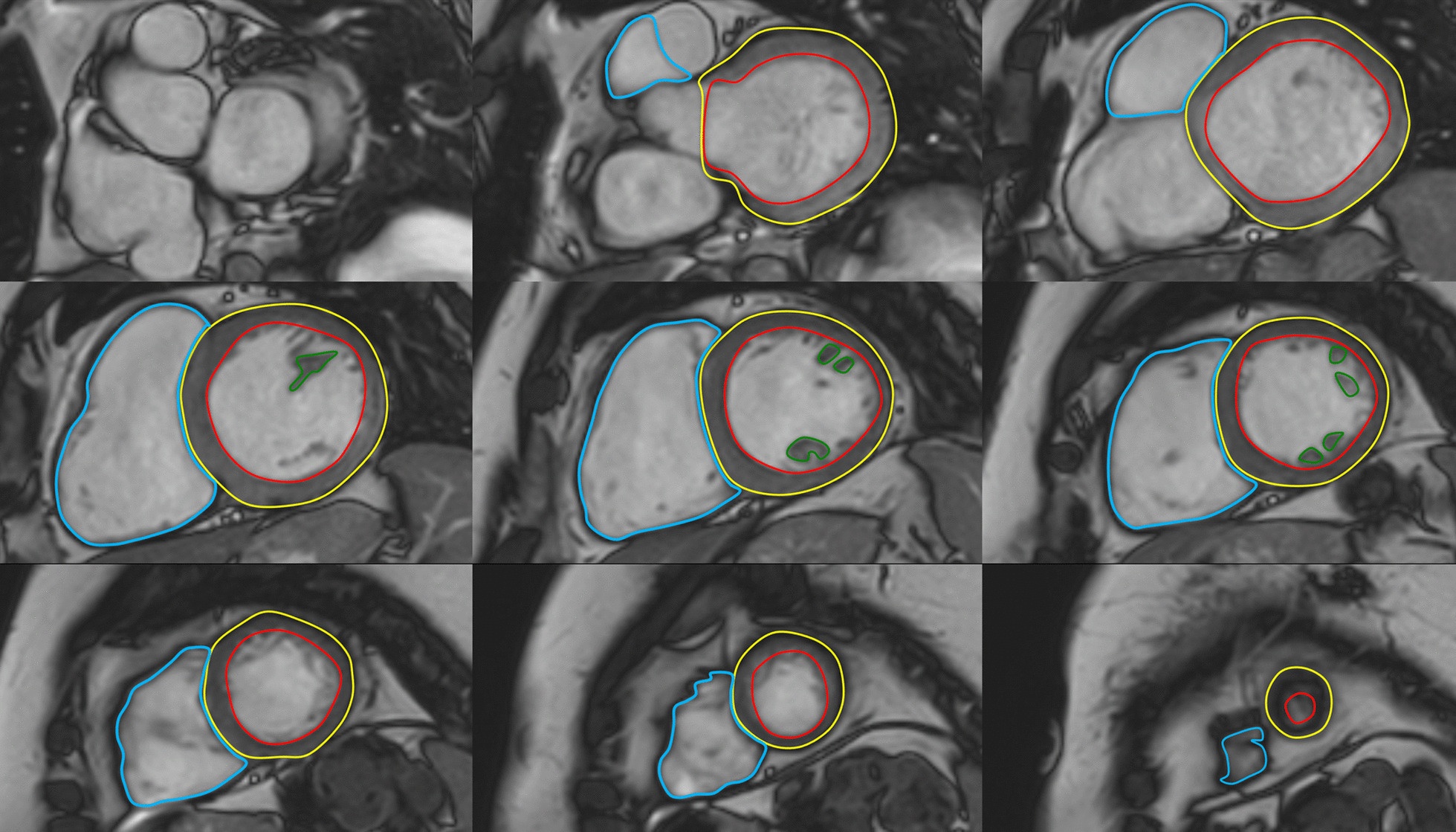
Contouring of the left ventricle (LV) and right ventricle (RV). Note that LV papillary muscle mass has been isolated and added to LV mass. RV papillary muscles and trabeculations were included in the RV volume

The majority of published articles used semi-automatic software for analysis of LV function and structure [10–16]. Short-axis images are most commonly analyzed on a per-slice basis, deriving LV mass and volume by applying the Simpson’s method (“stack of disks”) [17]. An example of LV contouring is shown in Fig. 1. Automated CMR analysis facilitated by machine learning is rapidly making inroads in LV volume and mass quantification [3]. The primary focus of early manuscripts has been on agreement between manual and automatic contouring [2]. However, to date, CMR variables for healthy cohorts have not been reported using machine learning methods.

**Fig. 2:**
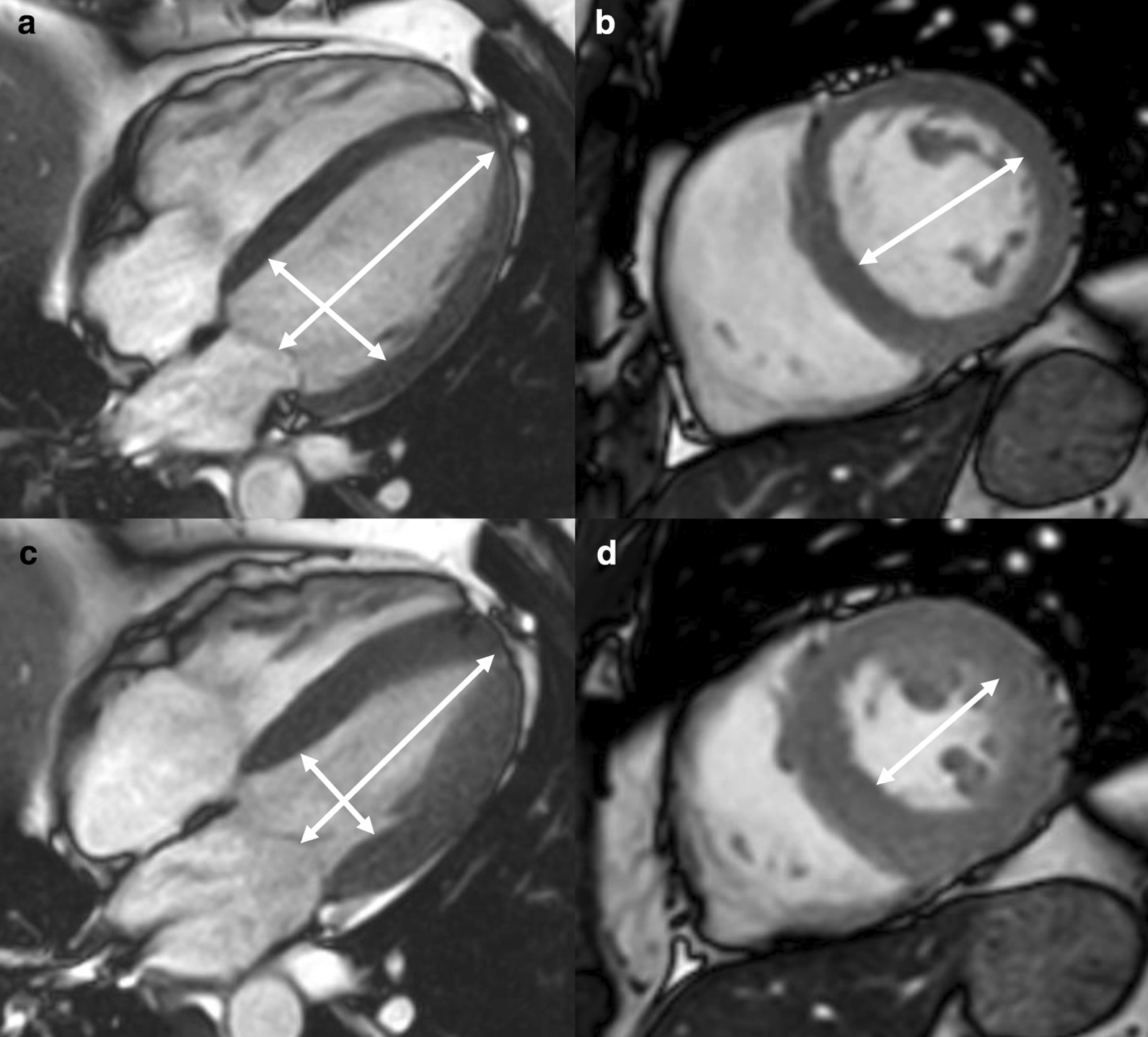
Measurements of LV diameters obtained on cine bSSFP images during diastole (**a**, **b**) and systole (**c**, **d**) on the 4 chamber view (**a**, **c**) and short axis view (**b**, **d**). The longitudinal diameter of the LV was measured on the 4 chamber view as the distance between the mitral valve plane and the LV apex (**a**, **c**). On the 4 chamber view the transverse diameter was defined as the distance between the septum and the lateral wall at the basal level [18]. On the short axis view the transverse diameter was obtained at the level of the basal papillary muscles (**b**, **d**) [15]

Measurements of LV diameter obtained on cine bSSFP images at diastole and systole on a 4 chamber view and short axis view are shown in Fig. 2.

### Demographic parameters

Gender is independently related to ventricular volumes and mass. Absolute and normalized volumes decrease in relationship to age in adults [10] in a continuous manner. For convenience, both average, and values per age decile are given in Tables 2, 3, 4 and 5 based on the peer-reviewed literature.

**Table 2:** Left ventricular parameters in the adult for men and women (ages 18–83), papillary muscles included in left ventricular mass

Parameter	Men				Women			
	n	Mean_p_	SD_p_	LL–UL^h^	n	Mean_p_	SD_p_	LL–UL^h^
LVEDV (ml)^a^	464	155	30	95–215	485	123	22	78–167
LVEDV/BSA (ml/m^2^)^b^	875	79	15	50–108	931	73	12	50–96
LVESV (ml)^a^	464	55	15	25–85	485	43	11	21–64
LVESV/BSA (ml/m^2^)^b^	875	29	9	11–47	931	25	7	10–40
LVSV (ml)^c^	410	103	21	61–145	432	83	16	52–114
LVSV/BSA (ml/m^2^)^d^	701	52	10	33–72	758	49	8	33–64
LVEF (%)^b^	875	64	8	49–79	931	66	7	52–79
LVM (g)^a^	464	121	28	66–176	485	83	21	41–125
LVM/BSA (g/m^2^)^e^	805	62	11	39–85	861	49	10	30–68
LVCO (l/min)^f^	91	5.6	1.1	3.4–7.8	89	4.5	0.9	2.7–6.3
LVCI (l/min/m^2^)^f^	91	3.0	0.6	1.8–4.2	89	2.9	0.5	1.9–3.9
LVM/LVEDV (g/ml)^g^	287	0.7	0.1	0.4–0.9	327	0.6	0.1	0.3–0.8

*n* number of study subjects included in the weighted mean values, *mean*_*p*_ pooled weighted mean, *SD*_*p*_ pooled standard deviation, *LL* lower limit, *UL* upper limit, *LV* left ventricular, *EDV* end-diastolic volume, *ESV* end-systolic volume, *SV* stroke volume, *EF* ejection fraction, *LVM* left ventricular mass, *CO* cardiac output, *CI* cardiac index, *BSA* body surface area

^a^Pooled weighted values from references [10, 11, 14, 22, 24]

^b^Pooled weighted values from references [10–14, 22, 24, 26]

^c^Pooled weighted values from references [10, 11, 14, 22]

^d^Pooled weighted values from references [10, 11, 13, 14, 22]

^e^Pooled weighted values from references [10, 11, 13, 14, 22, 24, 26]

^f^Values from reference [11]

^g^Pooled weighted values from references [11, 14]

^h^Calculated as mean_p_ ± 2*SD_p_

**Table 3:** Left ventricular parameters for adult men by age group, papillary muscles included in left ventricular mass

Parameter	20–29 years		30–39 years		40–49 years		50–59 years		60–69 years	
	n	Mean_p_ ± SD_p_ (LL–UL)^c^	n	Mean_p_ ± SD_p_ (LL–UL)^c^	n	Mean_p_ ± SD_p_ (LL–UL)^c^	n	Mean_p_ ± SD_p_ (LL–UL)^c^	n	Mean_p_ ± SD_p_ (LL–UL)^c^
LVEDV/BSA (ml/m^2^)	51^a^	86 ± 13 (61–112)	105^a^	81 ± 11 (59–103)	110^a^	83 ± 14 (55–110)	78^a^	77 ± 14 (49–105)	34 ^b^	78 ± 11 (57–99)
LVESV/BSA (ml/m^2^)	51^a^	34 ± 10 (14–53)	105^a^	30 ± 8 (15–46)	110^a^	32 ± 9 (13–50)	78^a^	29 ± 8 (12–45)	34 ^b^	30 ± 8 (13–46)
LVSV/BSA (ml/m^2^)	41^b^	54 ± 7 (40–68)	93^b^	51 ± 8 (34–67)	101^b^	52 ± 8 (36–68)	63^b^	49 ± 10 (30–69)	34 ^b^	48 ± 8 (34–63)
LVEF (%)	51^a^	60 ± 7 (46–74)	105^a^	63 ± 7 (49–77)	110^a^	62 ± 7 (48–76)	78^a^	63 ± 7 (49–78)	34 ^b^	62 ± 7 (48–76)
LVM/BSA (g/m^2^)	51^a^	66 ± 11 (44–87)	105^a^	64 ± 11 (41–86)	110^a^	64 ± 10 (43–84)	78^a^	62 ± 10 (42–83)	34 ^b^	62 ± 12 (38–87)

*n* number of study subjects included in the weighted mean values, *mean*_*p*_ pooled weighted mean, *SD*_*p*_ pooled standard deviation, *LL* lower limi, *UL* upper limit, *LV* left ventricular, *EDV* end-diastolic volume, *ESV* end-systolic volume, *SV* stroke volume, *EF* ejection fraction, *LVM* left ventricular mass, *BSA* body surface area

^a^Pooled weighted values from references [10, 13, 24]

^b^Pooled weighted values from references [10, 13]

^c^Calculated as mean_p_ ± 2*SD_p_

**Table 4:** Left ventricular parameters for adult women by age group, papillary muscles included in left ventricular mass

Parameter	20–29 years		30–39 years		40–49 years		50–59 years		60–69 years	
	n	Mean_p_ ± SD_p_ (LL–UL)^c^	n	Mean_p_ ± SD_p_ (LL–UL)^c^	n	Mean_p_ ± SD_p_ (LL–UL)^c^	n	Mean_p_ ± SD_p_ (LL–UL)^c^	n	Mean_p_ ± SD_p_ (LL–UL)^c^
LVEDV/BSA (ml/m^2^)	43^a^	77 ± 12 (54–100)	110^a^	77 ± 13 (52–102)	127^a^	73 ± 12 (50–96)	93^a^	68 ± 10 (48–89)	41^b^	68 ± 8 (51–84)
LVESV/BSA (ml/m^2^)	43^a^	29 ± 7 (16–43)	110^a^	29 ± 10 (9–49)	127^a^	27 ± 7 (12–42)	93^a^	24 ± 7 (10–38)	41^b^	25 ± 5 (14–35)
LVSV/BSA (ml/m^2^)	33^b^	50 ± 6 (38–63)	92^b^	49 ± 7 (34–64)	116^b^	48 ± 8 (32–64)	84^b^	47 ± 6 (34–59)	41^b^	44 ± 7 (31–58)
LVEF (%)	43^a^	62 ± 6 (50–73)	110^a^	64 ± 6 (52–77)	127^a^	63 ± 7 (50–76)	93^a^	65 ± 6 (52–78)	41^b^	65 ± 6 (53–77)
LVM/BSA (g/m^2^)	43^a^	51 ± 11 (29–72)	110^a^	50 ± 9 (32–68)	127^a^	49 ± 9 (32–66)	93^a^	51 ± 10 (31–70)	41^b^	52 ± 11 (31–74)

*n* number of study subjects included in the weighted mean values, *mean*_*p*_ pooled weighted mean, *SD*_*p*_ pooled standard deviation, *LL* lower limit, *UL* upper limit, *LV* left ventricular, *EDV* end-diastolic volume, *ESV* end-systolic volume, *SV* stroke volume, *EF* ejection fraction, *LVM* left ventricular mass, *BSA* body surface area

^a^Pooled weighted values from references [10, 13, 24]

^b^Pooled weighted values from references [10, 13]

^c^Calculated as mean_p_ ± 2*SD_p_

**Table 5:** Left ventricular parameters in the adult for men and women (ages 16–83), papillary muscles included in left ventricular volume

Parameter	Men				Women			
	n	Mean_p_	SD_p_	LL–UL^g^	n	Mean_p_	SD_p_	LL–UL^g^
LVEDV (ml)^a^	832	145	31	83–207	1064	112	21	70–155
LVEDV/BSA (ml/m^2^)^b^	832	77	15	47–107	1064	69	12	45–93
LVESV (ml)^a^	832	53	18	19–88	1064	39	12	15–64
LVESV/BSA (ml/m^2^)^b^	832	29	9	11–47	1064	24	7	10–38
LVSV (ml)^a^	832	91	18	55–127	1064	73	13	47–99
LVSV/BSA (ml/m^2^)^c^	772	48	9	30–66	1004	45	7	30–59
LVEF (%)^b^	832	63	6	51–76	1064	66	7	52–79
LVM (g)^a^	832	105	24	57–152	1064	73	15	43–103
LVM/BSA (g/m^2^)^b^	832	56	10	36–75	1064	45	7	30–59
LVCO (l/min)^d^	464	6.1	1.1	3.9–8.3	632	4.9	1.0	3.0–6.9
LVCI (l/min/m^2^)^e^	404	3.2	0.6	2.1–4.3	572	2.9	0.5	1.9–4.0
LVM/LVEDV (g/ml)^f^	708	0.7	0.2	0.3–1.2	944	0.7	0.1	0.4–1.0

*n* number of study subjects included in the weighted mean values, *mean*_*p*_ pooled weighted mean, *SD*_*p*_ pooled standard deviation, *LV* left ventricular, *EDV* end-diastolic volume, *ESV* end-systolic volume, *SV* stroke volume, *EF* ejection fraction, *LVM* left ventricular mass, *CO* cardiac output, *CI* cardiac index, *BSA* body surface area

^a^Pooled weighted values from references [15, 16, 19, 23, 25]

^b^Pooled weighted values from references [15, 16, 18, 19, 23, 25]

^c^Pooled weighted values from references [16, 18, 19, 23, 25]

^d^Pooled weighted values from references [15, 23, 25]

^e^Pooled weighted values from references [23, 25]

^f^Pooled weighted values from references [16, 25]

^g^Calculated as mean_p_ ± 2*SD_p_

### Studies included in this review

Multiple studies have presented cohorts of normal individuals for determining normal LV dimensions . For the purpose of this review, only cohorts of 40 or more normal subjects stratified by gender using bSSFP CMR technique at 1.5 or 3 T have been included. In addition, a full description of the subject cohort (including the analysis methods used), age and gender of subjects was required to be included for this review. Two studies [18, 19] included papillary muscles in LV volume except if directly attached to the LV wall, in which case they were included in LV mass (LVM) instead. Since this approach was inconsistent with post-processing recommended by SCMR [9] and other manuscripts on the topic, both studies were excluded from the current analysis. Data at 1.5 and 3 T is now available for normal subjects using bSSFP short axis imaging. Since it has been shown that parameters of LV volumes and function do not vary by field strength, calculation of the weighted means of these parameters include studies performed at 1.5 T and 3 T [20]. Information on ethnicity in relationship to LV parameters is not available for the majority of papers reporting the bSSFP technique and is therefore not reported in this review. However, small differences in LV parameters by ethnicity have been reported in the Multi-ethnic Study of Atherosclerosis (MESA) study; for further information on the magnitude of such differences, the reader is referred to the work by Natori S et al. [21].

Normal adult values for LV dimensions and functions according to those studies that consistently included papillary muscles in the LVM are presented in Tables 2, 3, 4, whereas those that consistently included papillary muscles in the LV volume are presented in Table 5. For parameters with sufficient sample size, values are also presented per age decile (Tables 3, 4).

### Additional left ventricular function parameters

In addition to left ventricular ejection fraction (LVEF), Maceira et al. have provided additional functional parameters that may be useful in some settings [10]. These are summarized in Table 6. For diastolic function, the derivative of the time/volume filling curve expresses the peak filling rate (PFR). Both early (E) and active (A) transmitral filling rates are provided. In addition, longitudinal atrioventricular plane descent (AVPD) and sphericity index (volume observed/volume of sphere using long axis as diameter) at end diastole and end systole are given. These latter parameters are not routinely used for clinical diagnosis. A number of publications have also reported LV end-diastolic and end-systolic diameters by CMR; these parameters are summarized in Table 7.

**Table 6:** Functional and geometric parameters of the normal left ventricle in the adult, from reference [10]

Parameter	Men (n = 60)			Women (n = 60)		
	Mean	SD	LL–UL^a^	Mean	SD	LL–UL^a^
PFRE (ml/s)	527	140	247–807	477	146	185–769
PFRE /BSA (ml/m^2^)	270	70	130–410	279	81	117–441
PFRE/EDV (/s)	3.4	0.7	2.0–4.8	3.8	0.8	2.2–5.4
PFRA (ml/s)	373	82	209–537	283	69	145–421
PFRA/BSA (ml/m^2^)	193	44	105–281	168	44	80–256
PFRA/EDV (/s)	2.6	0.6	1.4–3.8	2.3	0.5	1.3–3.3
PFRE/PFRA	1.4	0.3	0.8–2.0	1.7	0.3	1.1–2.3
Septal AVPD (mm)	15	4	7–23	14	3	8–20
Septal AVPD /long length (%)	15	3	9–21	16	4	8–24
Lateral AVPD (mm)	18	4	10–26	17	3	11–23
Lateral AVPD /long length (%)	17	3	11–23	19	3	13–25
Sphericity index, diastole^b^	0.31	0.07	0.20–0.48	0.34	0.07	0.20–0.48
Sphericity index, systole	0.20	0.05	0.1–0.3	0.23	0.07	0.09–0.37

*n* number of study subjects, *SD* standard deviation, *LL* lower limit, *UL* upper limit, *BSA* body surface area, *PFR* peak filling rate, *E* early, *A* active, *AVPD* atrioventricular plane descent

^a^Calculated as mean ± 2*SD

^b^Pooled weighted mean and SD calculated from references [10, 18] with n = 195 men and n = 233 women.

**Table 7:** Left ventricular diameters in the adult for men and women, bSSFP technique

Parameter	Men				Women			
	n	Mean_p_	SD_p_	LL–UL^e^	n	Mean_p_	SD_p_	LL–UL^e^
LV end-diastolic diameter 4Ch (mm)^a^	227	52	5	42–62	188	49	5	39–59
LV end-diastolic diameter SAx (mm)^b^	400	53	5	44–62	572	49	4	41–57
LV end-systolic diameter 4Ch (mm)^c^	54	32	3	26–38	53	28	6	16–40
LV end-systolic diameter SAx (mm)^d^	60	34	3	28–40	60	31	4	23–39

*bSSFP* balanced steady-state free precession, *n* number of study subjects included in the weighted mean values, *mean*_*p*_ pooled weighted mean, *SD*_*p*_ pooled standard deviation, *LL* lower limit, *UL* upper limit, *LV* left ventricular, *4Ch* 4 chamber view, *SAx* short axis

^a^Pooled weighted values from references [18, 24]

^b^Pooled weighted values from references [15, 25]

^c^Values from reference [24]

^d^Values from reference [15]

^e^Calculated as mean_p_ ± 2*SD_p_

## Right ventricular dimensions and functions in the adult

### CMR acquisition parameters

For measurement of right ventricular (RV) volumes, a stack of cine bSSFP images is acquired either in the short axis plane or transaxial plane [9].

### CMR analysis methods

Similar to the LV, analysis of the RV is usually performed on a per slice basis by manual contouring of the endocardial and epicardial borders. Volumes are calculated based on the Simpson’s method [17]. The RV volumes and mass are significantly affected by inclusion or exclusion of trabeculations and papillary muscles [27, 28]. For manual contouring, inclusion of trabeculations and papillary muscles as part of the RV volume will achieve higher reproducibility [9, 27, 28]. However, semiautomatic software is increasingly used for volumetric analysis, enabling automatic delineation of papillary muscles [29]. Therefore, normal values for both methods are provided. An example for RV contouring is shown in Fig. 1. Detailed recommendations for RV acquisitions and post processing have been published [9].

### Demographic parameters

RV mass and volumes are dependent on body surface area (BSA) [14, 29]. Absolute and RV volumes indexed by BSA are significantly larger in males compared to females [11, 14, 16, 18, 22, 29]. Further, RV volumes decrease with greater age [11, 14, 16, 18, 22, 29].

### Studies included in this review

Criteria regarding study inclusion are identical compared to the LV. Nine studies based on bSSFP imaging were included (Table 8). In one study, papillary muscles were included as part of the RV mass and excluded from the RV volume [29] with results presented for men and women in Table 9. In the remaining eight studies, the papillary muscles were included as part of the RV cavity volume rather than included in the RV mass [11, 14–16, 18, 22–24] with pooled weighted mean values presented for men and women (Table 10). For a subset of three of these studies [18, 23, 24], for parameters with a sufficient sample size pooled weighted mean values are presented based on age deciles between 20 and 59 years of age for both men (Table 11) and women (Table 12).

**Table 8:** References, normal right ventricular volumes, function and dimensions in the adult

First author, year	CMR technique	n, male:female	Age range (years)
Hudsmith, 2005 [22]	1.5 T, short axis bSSFP, papillary muscles included in RV volume	63:45	21–68
Maceira, 2006 [29]	1.5 T, short axis bSSFP, papillary muscles included in RV mass	60:60	20–80
Chang, 2012 [23]	1.5 T, short axis bSSFP, papillary muscles included in RV volume	64:60	20–70
Macedo, 2013 [24]	1.5 T, short axis bSSFP, papillary muscles included in RV volume	54:53	20–80
Le Ven, 2015 [14]	1.5 T, short axis bSSFP, papillary muscles included in RV volume	196:238	18–36
Lei, 2016 [15]	3 T, short axis bSSFP, papillary muscles included in RV volume	60:60	23–83
Le, 2016 [11]	3 T, short axis bSSFP, papillary muscles included in RV volume	91:89	20–69
Aquaro, 2017 [18]	1.5 T, short axis bSSFP, papillary muscles included in RV volume	173:135	16– > 60
Petersen, 2017 [16]	1.5 T, short axis bSSFP, papillary muscles included in RV volume	368:432	45–74

*n* number of study subjects, *bSSFP* balancedsteady-state free precession, *RV* right ventricular

**Table 9:** Right ventricular parameters in the adult for men and women (ages 20–79), papillary muscles included in right ventricular mass, from reference [29]

Parameter	Men (n = 60)			Women (n = 60)		
	Mean	SD	LL–UL^a^	Mean	SD	LL–UL^a^
RVEDV (ml)	163	27	109–217	127	24	79–175
RVEDV/BSA (ml/m^2^)	83	13	58–109	74	12	51–97
RVESV (ml)	57	17	23–91	44	15	13–75
RVESV/BSA (ml/m^2^)	29	9	12–46	26	8	9–42
RVSV (ml)	106	18	71–141	83	13	56–110
RVSV/BSA (ml/m^2^)	54	8	38–71	48	7	35–61
RVEF (%)	66	7	51–80	66	7	52–80
RVM (g)	66	15	37–95	48	11	26–71
RVM/BSA (g/m^2^)	34	7	20–48	28	6	16–40

*n* number of study subjects, *SD* standard deviation, *LL* lower limit, *UL* upper limit, *RV* right ventricular, *EDV* end-diastolic volume, *ESV* end-systolic volume, *SV* stroke volume, *EF* ejection fraction, *RVM* right ventricular mass, *BSA* body surface area

^a^Calculated as mean ± 2*SD

**Table 10:** Right ventricular parameters in the adult for men and women (ages 20–83), papillary muscles included in right ventricular volume

Parameter	Men				Women			
	n	Meanp	SDp	LL–ULg	n	Meanp	SDp	LL–ULg
RVEDV (ml)^a^	896	166	39	87–244	977	122	27	68–176
RVEDV/BSA (ml/m^2^)^b^	1069	88	17	53–123	1112	76	14	48–104
RVESV (ml)^a^	896	73	22	29–117	977	50	15	20–80
RVESV/BSA (ml/m^2^)^b^	1069	38	11	17–59	1112	30	9	13–48
RVSV (ml)^c^	842	95	26	43–146	924	74	18	39–109
RVSV/BSA (ml/m^2^)^d^	955	52	12	28–75	999	48	9	29–66
RVEF (%)^b^	1069	57	8	42–72	1112	60	7	46–74
RVM (g)^e^	117	36	9	17–54	98	30	9	13–48
RVM/BSA (g/m^2^)^e^	117	19	4	10–28	98	17	5	7–28
RVCO (l/min)^f^	155	5.6	1.4	2.8–8.3	149	4.4	1.0	2.4–6.4
RVCI (l/min/m^2^)^f^	155	3.0	0.7	1.5–4.5	149	2.8	0.6	1.6–4.0

*n* number of study subjects included in the weighted mean values, *mean*_*p*_ pooled weighted mean, *SD*_*p*_ pooled standard deviation, *LL* lower limit, *UL* upper limit, *RV* right ventricular, *EDV* end-diastolic volume, *ESV* end-systolic volume, *SV* stroke volume, *EF* ejection fraction, *RVM* right ventricular mass, *CO* cardiac output, *CI* cardiac index, *BSA* body surface area

^a^Pooled weighted values from references [11, 14–16, 22–24]

^b^Pooled weighted values from references [11, 14–16, 18, 22–24]

^c^Pooled weighted values from references [11, 14–16, 22, 23]

^d^Pooled weighted values from references [11, 14, 16, 18, 22, 23]

^e^Pooled weighted values from references [22, 24]

^f^Pooled weighted values from references [11, 23]

^g^Calculated as mean_p_ ± 2*SD_p_

**Table 11:** Right ventricular parameters for adult men by age group, papillary muscles included in right ventricular volume

Parameter	20–29 years		30–39 years		40–49 years		50–59 years	
	n	Mean_p_ ± SD_p_ (LL–UL)^c^	n	Mean_p_ ± SD_p_ (LL–UL)^c^	n	Mean_p_ ± SD_p_ (LL–UL)^c^	n	Mean_p_ ± SD_p_ (LL–UL)*
RVEDV/BSA (ml/m^2^)^a^	50	94 ± 15 (63–124)	55	83 ± 13 (57–109)	49	81 ± 16 (50–112)	55	80 ± 16 (48–111)
RVESV/BSA (ml/m^2^)^a^	50	44 ± 11 (23–66)	55	38 ± 8 (22–53)	49	34 ± 8 (18–49)	55	35 ± 10 (16–54)
RVSV/BSA (ml/m^2^)^b^	40	51 ± 13 (26–77)	43	46 ± 10 (27–65)	40	44 ± 11 (23–65)	40	51 ± 13 (24–78)
RVEF (%)^a^	50	52 ± 8 (36–69)	55	55 ± 7 (41–68)	49	57 ± 8 (40–73)	55	57 ± 8 (41–74)

*n* number of study subjects included in the weighted mean values, *mean*_*p*_ pooled weighted mean, *SD*_*p*_ pooled standard deviation, *LL* lower limit, *UL* upper limit, *RV* right ventricular, *EDV* end-diastolic volume, *ESV* end-systolic volume, *SV* stroke volume, *EF* ejection fraction, *BSA* body surface area

^a^Pooled weighted values from references [18, 23, 24]

^b^Pooled weighted values from references [18, 23]

^c^Calculated as mean_p_ ± 2*SD_p_

**Table 12:** Right ventricular parameters for adult women by age group, papillary muscles included in right ventricular volume

Parameter	20–29 years		30–39 years		40–49 years		50–59 years	
	n	Mean_p_ ± SD_p_ (LL–UL)^c^	n	Mean_p_ ± SD_p_ (LL–UL)^c^	n	Mean_p_ ± SD_p_ (LL–UL)^c^	n	Mean_p_ ± SD_p_ (LL–UL)*
RVEDV/BSA (ml/m^2^) ^a^	47	78 ± 12 (55–101)	51	76 ± 12 (51–100)	46	74 ± 14 (46–102)	46	69 ± 13 (42–95)
RVESV/BSA (ml/m^2^) ^a^	47	33 ± 12 (10–56)	51	31 ± 8 (15–48)	46	29 ± 8 (13–45)	46	28 ± 8 (11–44)
RVSV/BSA (ml/m^2^)^b^	37	46 ± 9 (28–63)	33	45 ± 12 (22–69)	35	47 ± 11 (24–69)	37	42 ± 10 (22–62)
RVEF (%)^a^	47	56 ± 11 (34–78)	51	58 ± 9 (39–77)	46	60 ± 8 (44–76)	46	61 ± 8 (44–78)

*n* number of study subjects included in the weighted mean values, *mean*_*p*_ pooled weighted mean, *SD*_*p*_ pooled standard deviation, *LL* lower limit, *UL* upper limit, *RV* right ventricular, *EDV* end-diastolic volume, *ESV* end-systolic volume, *SV* stroke volume, *EF* ejection fraction, *BSA* body surface area

^a^Pooled weighted values from references [18, 23, 24]

^b^Pooled weighted values from references [18, 23]

^c^Calculated as mean_p_ ± 2*SD_p_

### Additional RV function parameters

Similar to the LV, Maceira et al. have provided additional functional parameters, including early and active peak filling rate and the longitudinal AVPD, that may have relevance to specific applications and can be found in the original publication [29].

## Left atrial dimensions and functions in the adult

### CMR acquisition parameters

There is limited consensus in the literature about how to measure left atrial (LA) volume. The most common methods to measure LA volume are the modified Simpson’s method (analogous to that used to measure LV and RV volumes) and the biplane area-length method [30]. Dedicated 3-dimensional modeling software has also been employed [31].

In the Simpson’s method, a stack of cine bSSFP images either in the SAx, the horizontal long axis or transverse view, is required. For 3-dimensional modeling a stack of SAx images has been used [31]. Evaluation by the biplane area-length method is based on a 2 and 4 chamber view [11, 16, 32–34].

**Fig. 3:**
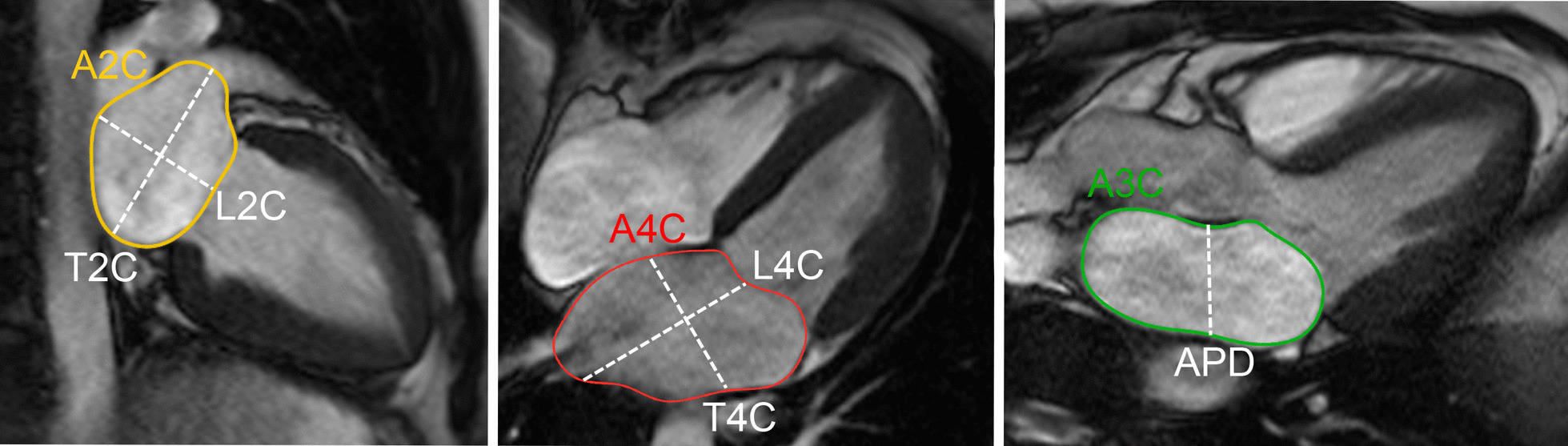
Measurement of left atrial area (A2Ch, A4Ch, A3C), longitudinal (L2Ch, L4Ch), transverse (T2Ch, T4Ch) and anteroposterior (APD) diameters on the 2-, 4- and 3-chamber views according to reference [31]

LA longitudinal and transverse diameters and area have been measured on 2, 3, and 4 chamber cine bSSFP images [31, 33, 35] (Fig. 3).

### CMR analysis methods

In many studies the LA appendage has been included as part of the LA volume and pulmonary veins are excluded [14, 31], but the practice of excluding both structures from the LA volume is increasingly gaining acceptance [11, 16, 32, 34].

The maximal LA volume is achieved during ventricular systole. In a cine acquisition, the maximum volume image can be defined as last image immediately before opening of the mitral valve. Accordingly the minimal LA volume image can be defined as the first image after closure of the mitral valve [36].

### Demographic parameters

Body surface area (BSA) has been shown to have a significant independent influence on LA volume and most diameters [31]. Per Sievers et al. [35], age was not an independent predictor of LA maximal volume or diameter in normal individuals. Men have a larger maximal LA volume compared to women [31, 35].

### Studies included in this review

There are nine publications for reference values of the adult LA (volume and/or diameter and/or area) based on bSSFP imaging with sufficient sample size (n > 40) and these are reported in Table 13. Four of these publications used the biplane area-length method, one used the Simpson’s method, one used both, one used a 3D modeling technique and the remainder measured diameters or areas. Publications reporting population-based cohort data rather than true normal data have been excluded from the current analysis as have publications that incompletely describe the measurement method used [22] or the manner in which pulmonary veins/LA appendage were handled. Normal values for  LA volumes and function are presented in Table 14, and normal values for LA diameters in Table 15.

**Table 13:** References, normal left atrial volumes, function and dimensions in the adult

First author, year	CMR technique	n, male:female	Age range (years)
Sievers, 2005 [35]	1.5 T, 2, 3 and 4 chamber bSSFP; measurement of diameters	59:52	25–73
Maceira, 2010 [31]	1.5 T, short axis, 2, 3 and 4 chamber bSSFP; 3D modeling and measurement of area and diameters; atrial appendage included, pulmonary veins excluded (for volume analysis)	60:60	20–80
Le, 2016 [11]	3 T, 2 and 4 chamber bSSFP; quantification of volume; biplane area-length method; atrial appendage and pulmonary veins excluded	91:89	20–69
Le Ven, 2016 [14]	1.5 T, short axis bSSFP; quantification of volume and function; Simpson’s method; atrial appendage included, pulmonary veins excluded	195:239	18–36
Aquaro, 2017 [18]	1.5 T, 4 chamber bSSFP; measurement of area	173:135	16– > 60
Li, 2017 [33]	3 T, short axis, 2, 3 and 4 chamber bSSFP; measurement of volume, function (biplane area-length and Simpson’s method atrial appendage excluded) and diameter	66:69	23–83
Petersen, 2017 [16]	1.5 T, 2 and 4 chamber bSSFP; quantification of volume and function; biplane area-length method; atrial appendage and pulmonary veins excluded	371:433	45–74
Zemrak, 2017 [34]	1.5 T, 2 and 4 chamber bSSFP; quantification of volume; biplane area-length method; atrial appendage and pulmonary veins excluded	109:174	(65 ± 9)^a^
Funk, 2018 [32]	1.5 T and 3 T, 2 and 4 chamber bSSFP; quantification of volume and function; biplane area-length method; atrial appendage and pulmonary veins excluded	105:77	19–76

*n* number of study subjects, *bSSFP* balanced steady-state free precession, *3D* 3-dimensional

^a^Mean ± SD (age-range not provided in original publication)

**Table 14:** Left atrial volumes and function in the adult for men and women, SSFP technique

Method	Parameter	Men				Women			
		n	Mean_p_	SD_p_	LL–UL^j^	n	Mean_p_	SD_p_	LL–UL^j^
Biplane area-length method; LA appendage excluded	Max. LA volume (ml)^a^	734	72	20	31–112	841	64	18	28–100
	Max. LA volume/BSA (ml/m^2^)^a^	734	38	11	17–59	841	39	11	17–61
	Min. LA volume (ml)^b^	171	25	10	6–44	146	22	8	7–38
	Min. LA volume/BSA (ml/m^2^)^c^	171	14	5	3–24	146	13	5	4–23
	LA stroke volume (ml)^d^	468	44	12	21–67	509	42	10	21–62
	LA stroke volume/BSA (ml/m^2^)^e^	363	22	6	10–34	432	22	6	10–34
	LA ejection fraction (%)^f^	534	62	8	46–77	578	63	8	48–78
Simpson’s method; LA appendage excluded	Max. LA volume (ml)^g^	66	70	15	40–99	69	66	13	39–93
	Max. LA volume/BSA (ml/m^2^)^g^	66	41	8	24–57	69	44	8	28–60
	Min. LA volume (ml)^g^	66	32	9	15–50	69	28	7	15–42
	Min. LA volume/BSA (ml/m^2^)^g^	66	19	5	9–28	69	19	4	11–27
	LA ejection fraction (%)^g^	66	54	8	38–70	69	57	6	45–69
Simpson’s method; LA appendage included	Max. LA volume (ml)^h^	256	78	18	42–115	298	66	14	37–94
	Max. LA volume/BSA (ml/m^2^)^h^	256	40	8	25–56	298	39	7	25–53
	Min. LA volume (ml)^i^	196	32	9	14–50	238	24	7	10–38
	Min. LA volume/BSA (ml/m^2^)^i^	196	17	4	9–25	238	15	4	7–23
	LA stroke volume (ml)^i^	196	47	13	21–73	238	39	10	19–59
	LA stroke volume/BSA (ml/m^2^)^i^	196	24	6	12–36	238	24	5	14–34
	LA ejection fraction (%)^i^	196	59	8	43–75	238	61	7	47–75

*n* number of study subjects included in the weighted mean values, *bSSFP* balanced steady-state free precession, *mean*_*p*_ pooled weighted mean, *SD*_*p*_ pooled standard deviation, *LL* lower limit, *UL* upper limit, *Max*. maximal, *Min*. minimal, *LA* left atrial, *BSA* body surface area

^a^Pooled weighted values from references [11, 16, 32–34]

^b^Pooled weighted values from references [22, 32, 33]

^c^Pooled weighted values from references [32, 33]

^d^Pooled weighted values from references [16, 32]

^e^Values from reference [16]

^f^Pooled weighted values from references [16, 22, 32, 33]

^g^Values from reference [33]

^h^Pooled weighted values from references [14, 31]

^i^Values from reference [14]

^j^Calculated as mean_p_ ± 2*SD_p_

**Table 15:** Left atrial diameter and area in the adult for men and women, bSSFP technique

Parameter	Men				Women			
	n	Mean_p_	SD_p_	LL–UL^f^	n	Mean_p_	SD_p_	LL–UL^f^
Max. LA area 2Ch (cm^2^)^a^	60	21	5	12–30	60	19	5	10–28
Max. LA area 2Ch/BSA (cm^2^/m^2^)^a^	60	11	2	6–16	60	11	2	6–16
Max. LA area 3Ch (cm^2^)^a^	60	19	4	12–26	60	17	4	10–24
Max. LA area 3Ch/BSA (cm^2^/m^2^)^a^	60	10	2	6–14	60	10	2	6–14
Max. LA area 4Ch (cm^2^)^b^	233	23	5	13–32	173	21	4	13–29
Max. LA area 4Ch/BSA (cm^2^/m^2^)^b^	233	12	2	7–16	195	12	2	8–15
Max. LA longitudinal diameter 2Ch (cm)^c^	185	4.9	0.7	3.5–6.2	181	4.6	0.7	3.3–5.9
Max. LA longitudinal diameter 2Ch/BSA (cm/m^2^)^c^	185	2.6	0.5	1.6–3.6	181	2.8	0.6	1.6–3.9
Max. LA transverse diameter 2Ch (cm)^d^	126	4.4	0.6	3.2–5.6	129	4.3	0.5	3.3–5.2
Max. LA transverse diameter 2Ch/BSA (cm/m^2^)^d^	126	2.4	0.3	1.7–3.0	129	2.7	0.3	2.2–3.2
Max. LA longitudinal diameter 3Ch (cm)^e^	66	5.5	0.6	4.2–6.8	69	5.4	0.7	4.0–6.7
Max. LA longitudinal diameter 3Ch/BSA (cm/m^2^)^e^	66	3.2	0.4	2.4–4.0	69	3.6	0.5	2.7–4.6
Max. LA antero-posterior diameter 3Ch (cm)^c^	185	3.0	0.5	2.0–4.0	181	3.0	0.5	2.0–4.0
Max. LA antero-posterior diameter 3Ch/BSA (cm/m^2^)^c^	185	1.6	0.3	1.0–2.2	181	1.8	0.4	1.1–2.5
Max. LA longitudinal diameter 4Ch (cm)^d^	126	5.8	0.6	4.6–7.1	129	5.5	0.6	4.2–6.8
Max. LA longitudinal diameter 4Ch/BSA (cm/m^2^)^d^	126	3.2	0.4	2.3–4.1	129	3.5	0.5	2.5–4.4
Max. LA transverse diameter 4Ch (cm)^c^	185	4.3	0.5	3.3–5.3	181	4.1	0.5	3.1–5.1
Max. LA transverse diameter 4Ch/BSA (cm/m^2^)^c^	185	2.2	0.3	1.6–2.9	181	2.5	0.4	1.8–3.2

*n* number of study subjects included in the weighted mean values, *bSSFP* balanced steady-state free precession, *mean*_*p*_ pooled weighted mean, *SD*_*p*_ pooled standard deviation, *LL* lower limit, *UL* upper limit, *Max*. maximal, *LA* left atrial, *2Ch* 2 chamber view, *3Ch* 3 chamber view, *4Ch* 4 chamber view, *BSA* body surface area

^a^Values from reference [31]

^b^Pooled weighted values from references [18, 31]

^c^Pooled weighted values from references [31, 33, 35]

^d^Pooled weighted values from references [31, 33]

^e^Values from reference [33]

^f^Calculated as mean_p_ ± 2*SD_p_

## Right atrial dimensions and functions in the adult

### CMR acquisition parameters

**Fig. 4:**
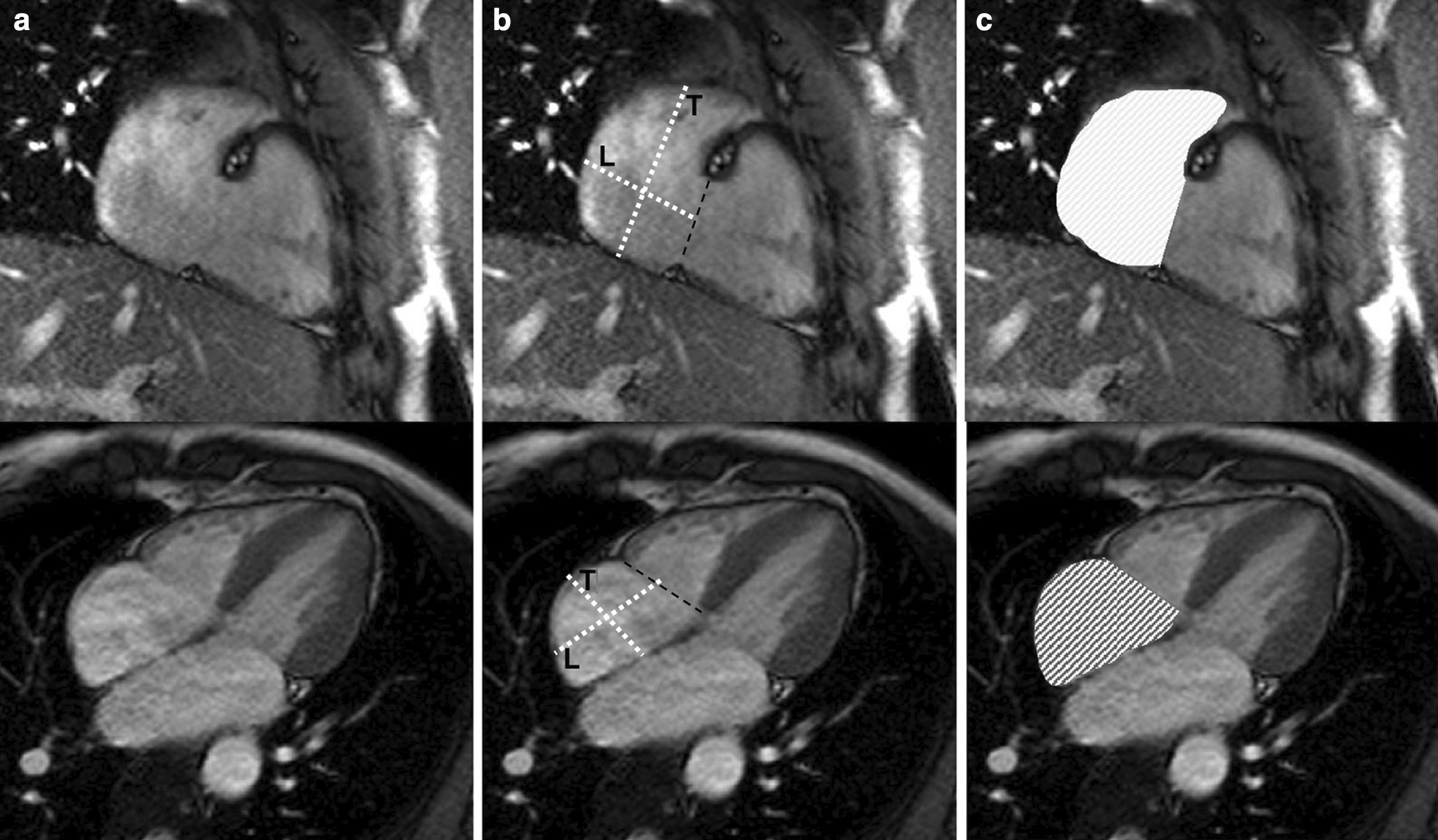
Measurement of right atrial (RA) parameters according to [37]. Areas and diameters were measured in atrial diastole (maximal size of the left atrium) on the 2-chamber (top row) and 4-chamber (bottom row) views. In B), longitudinal diameter (L) is obtained from the posterior wall of the RA to the center of the tricuspid plane, and transverse diameter (T) is obtained perpendicular to the longitudinal diameter, at the mid level of the RA. C shows measurements of the area for both views including the RA appendage

There is no consensus in the literature regarding acquisition and measurement method for the right atrium (RA). Published methods for RA volume include the modified Simpson’s method, the biplane area-length method and 3D-modeling [23, 24, 37]. For Simpson’s method and 3D modeling, a stack of cine bSSFP images in the SAx view are analyzed. For the biplane area-length method, a 4-chamber view and a RV 2-chamber view are utilized [33] (Fig. 4).

### CMR analysis methods

The inferior and superior vena cava are excluded from the RA volume but there is variability in the inclusion [14, 37] or exclusion [33] of the RA appendage.

The maximal RA volume is achieved during ventricular systole and can be defined as the last cine image before opening of the tricuspid valve. The minimal RA volume can be defined as the first cine image after closure of the tricuspid valve.

### Demographic parameters

Maceira et al. demonstrated the relationship of most RA parameters to BSA, but there was no influence of age on atrial parameters and no influence of gender on atrial volumes [37]. Other studies have demonstrated an influence of gender [14, 33] and age [11, 33] on some RA parameters. In the study by LeVen et al. gender was independently associated with RA end-diastolic volume and RA end-systolic volume with men having greater values compared to women [14]. In the study by Li et al. the longitudinal RA diameter measured in the 2 chamber and 4 chamber view indexed to BSA and the indexed transverse diameter measured on the 4 chamber view were greater in women than in men [33]. Further, the RA volume indexed to BSA was larger in males than in females [33]. Le et al. found a week correlation between the RA area indexed to BSA with age [11].

### Studies included in this review

There are five publications with reference values for the RA based on bSSFP imaging with sufficient sample size to be included [11, 14, 18, 33, 37] (Table 16). Pooled weighted mean values for RA volumes and function are provided in Table 17 using the biplane area-length method (RA appendage excluded) or Simpson’s method (either RA appendage included or excluded) for men and women. Pooled weighted mean values for RA areas and diameters are provided in Table 18 for men and women.

**Table 16:** References, normal right atrial volumes, function and dimensions in the adult

First author, year	CMR technique	n, male:female	Age range (years)
Maceira, 2013 [37]	1.5 T, short axis, RV 2 chamber and 4 chamber bSSFP, 3D modeling and measurement of area and diameters, atrial appendage included for volume analysis	60:60	20–80
Le Ven, 2015 [14]	1.5 T, short axis bSSFP, quantification of volume and function (Simpson’s method), atrial appendage included	196:238	25–73
Le, 2016 [11]	3.0 T, 4 chamber bSSFP, measurement of area	91:89	20–69
Aquaro, 2017 [18]	1.5 T, 4 chamber bSSFP, measurement of area	173:135	16– > 60
Li, 2017 [33]	3.0 T, Short axis, RV 2 chamber and 4 chamber bSSFP, measurement of diameter, volume and function (biplane area-length and Simpson’s method), atrial appendage excluded	66:69	23–83

*n* number of study subject, *bSSFP* balanced steady-state free precession, *RV* right ventricular

**Table 17:** Right atrial volumes and function in the adult for men and women

Method	Parameter	Men				Women			
		N	Mean_p_	SD_p_	LL–UL^d^	n	Mean_p_	SD_p_	LL–UL^d^
Biplane area-length method; RA appendage excluded	Max. RA volume (ml)^a^	66	65	20	24–105	69	53	14	24–81
	Max. RA volume/BSA (ml/m^2^)^a^	66	38	12	15–61	69	35	10	16–54
	Min. RA volume (ml)^a^	66	32	12	9–55	69	23	7	9–37
	Min. RA volume/BSA (ml/m^2^)^a^	66	19	7	5–32	69	15	5	6–24
	RA ejection fraction (%)^a^	66	50	9	32–68	69	56	9	38–74
Simpson’s method; RA appendage excluded	Max. RA volume (ml)^a^	66	89	22	46–132	69	77	16	45–108
	Max. RA volume/BSA (ml/m^2^)^a^	66	52	12	28–76	69	51	10	31–71
	Min. RA volume (ml)^a^	66	46	16	14–79	69	35	9	17–53
	Min. RA volume/BSA (ml/m^2^)^a^	66	27	9	9–45	69	23	6	12–35
	RA ejection fraction (%)^a^	66	49	10	29–69	69	54	9	36–72
Simpson’s method; RA appendage included	Max. RA volume (ml)^b^	256	108	25	59–158	298	85	18	49–122
	Max. RA volume/BSA (ml/m^2^)^b^	256	56	12	32–79	298	50	10	31–69
	Min. RA volume (ml)^c^	196	50	17	16–84	238	33	11	11–55
	Min. RA volume/BSA (ml/m^2^)^c^	196	26	8	10–42	238	20	6	8–32
	RA stroke volume (ml)^c^	196	58	16	26–90	238	47	12	23–71
	RA stroke volume/BSA (ml/m^2^)^c^	196	30	8	14–46	238	28	7	14–42
	RA ejection fraction (%)^c^	196	54	10	34–74	238	59	9	41–77

*n* number of study subjects included in the weighted mean values, *mean*_*p*_ pooled weighted mean, *SD*_*p*_ pooled standard deviation, *LL* lower limit, *UL* upper limit, *Max*. maximal, *Min*. minimal, *RA* right atrial, *BSA* body surface area

^a^Values from reference [33]

^b^Pooled weighted values from references [14, 37]

^c^Values from reference [14]

^d^Calculated as mean_p_ ± 2*SD_p_

**Table 18:** Right atrial diameter and area in the adult for men and women, bSSFP technique

Parameter	Men				Women			
	n	Mean_p_	SD_p_	LL–UL^d^	n	Mean_p_	SD_p_	LL–UL^d^
Max. RA area 2Ch (cm^2^)^a^	60	23	4	15–31	60	21	4	13–29
Max. RA area 2Ch/BSA (cm^2^/m^2^)^a^	60	12	2	7–17	60	12	2	7–17
Max. RA area 4Ch (cm^2^)^b^	324	21	4	13–30	284	19	3	12–26
Max. RA area 4Ch/BSA (cm^2^/m^2^)^b^	324	11	2	7–15	284	12	2	8–15
Max. RA longitudinal diameter 2Ch (cm)^c^	126	5.5	0.6	4.2–6.7	129	5.1	0.6	3.9–6.3
Max. RA longitudinal diameter 2Ch/BSA (cm/m^2^)^c^	126	3.0	0.4	2.3–3.7	129	3.2	0.4	2.3–4.1
Max. RA transverse diameter 2Ch (cm)^c^	126	4.2	0.9	2.4–6.0	129	4.1	0.9	2.4–5.9
Max. RA transverse diameter 2Ch/BSA (cm/m^2^)^c^	126	2.3	0.5	1.3–3.3	129	2.6	0.6	1.5–3.7
Max. RA longitudinal diameter 4Ch (cm)^c^	126	5.3	0.6	4.0–6.6	129	5.1	0.6	4.0–6.3
Max. RA longitudinal diameter 4Ch/BSA (cm/m^2^)^c^	126	2.9	0.4	2.2–3.7	129	3.2	0.4	2.4–4.0
Max. RA transverse diameter 4Ch (cm)^c^	126	4.8	0.6	3.7–5.9	129	4.3	0.6	3.2–5.4
Max. RA transverse diameter 4Ch/BSA (cm/m^2^)^c^	126	2.6	0.3	2.1–3.2	129	2.7	0.3	2.0–3.4

*n* number of study subjects included in the weighted mean values, *mean*_*p*_ pooled weighted mean, *SD*_*p*_ pooled standard deviation, *LL* lower limit, *UL* upper limit, *Max*. maximal, *RA* right atrial, *2Ch* 2 chamber view, *3Ch* 3 chamber view, *4Ch* 4 chamber view, *BSA* body surface area

^a^Values from reference [37]

^b^Pooled weighted values from references [11, 18, 37]

^c^Pooled weighted values from references [33, 37]

^d^Calculated as mean_p_ ± 2*SD_p_

### Additional RA function parameters

Reference ranges for parameters characterizing RA function, including the reservoir, conduit and pump function, can be found in a separate publication by Maceira et al. [38].

## Left and right ventricular dimensions and function in children

The presentation of normal values in children is different than in the adult population due to continuous changes in body weight and height as a function of age. Normal data in children are frequently presented in percentiles and/or z-scores (standard deviation score). Z-scores are given as

$${\text{z - value}} = \left( {{\text{measurement}}{-}{\text{mean of the population}}} \right)/\left( {\text{standard deviation of the mean of the population}} \right).$$

Even though previous studies [39–41] have reported a linear correlation between ventricular volumes and BSA in children, there is increasing evidence that the assumption of a simple linear or exponential relationship between somatic growth and age may not be correct. Moreover the relationship between cardiac growth and body growth is still not clearly understood and may vary along age in the developing child [42, 43].

The construction of reference curves using the Lamda-Mu-Sigma (LMS) method is a different way of creating normalized growth percentile curves. In this approach after a power transformation skewness of the data can be transformed into normality and trends are summarized in a smooth curve (L); trends in the mean (M) and coefficient of variation (S) are similarly smoothed. LMS curves are easy to use in daily practice and can account for nonlinear relationships between body and cardiac size and age.

The LMS method is highly efficient to obtain normality in small datasets, for instance in the group of young children. Thus, even extreme values (small children) can be so converted into exact standard deviation scores [44].

### Demographic parameters

The largest cohort of normal data on ventricular size and function in paediatric patients using the bSSFP sequence refers to a population of 141 healthy children collected in three European reference centers. All subjects were Caucasian and included 68 boys and 73 girls. Age distribution, body size and heart rate were equal between genders. Only 12/141 children were younger than 6 years [45].

Boys had larger ventricles than girls [45]. LVEF was found to be slightly higher in boys (67% vs 65%; p 0.01), but not for the RV [45]**.** Gender differences are more marked in older children, indicating that gender is more important after puberty and in adulthood.

### Studies included in this review

**Fig. 5:**
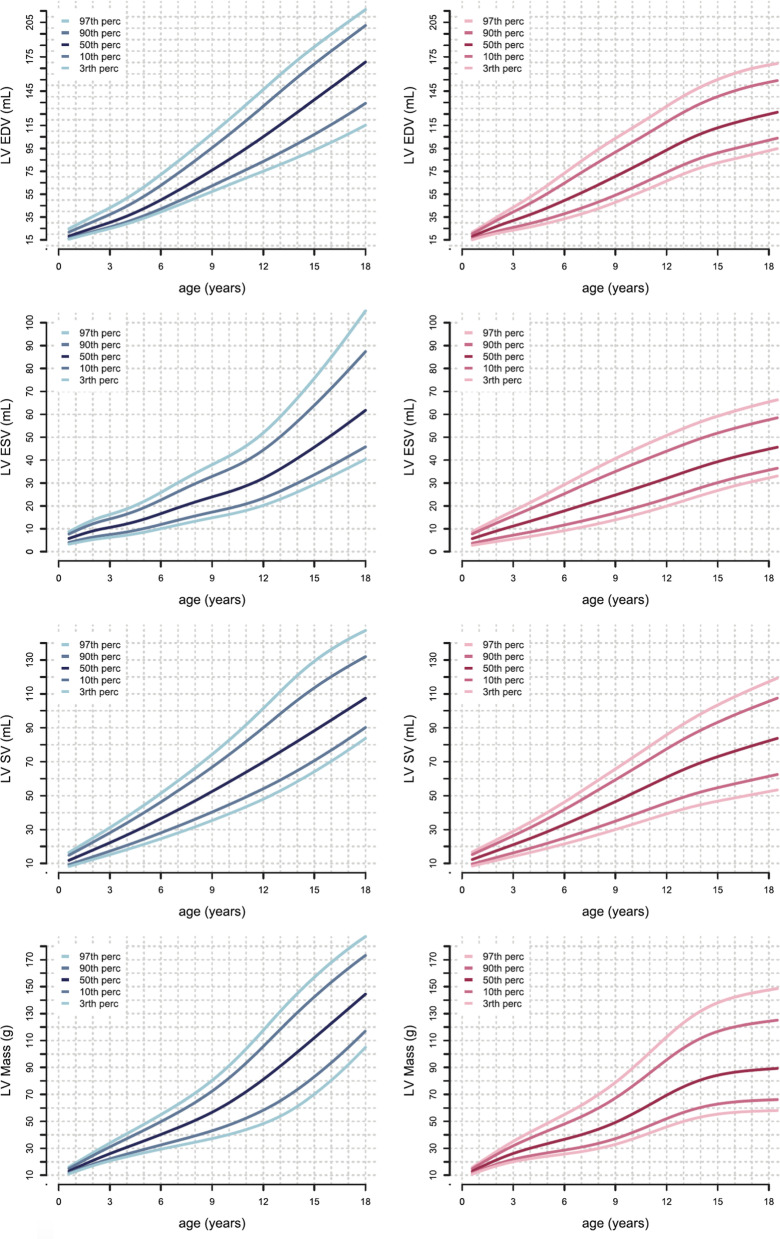
Reference curves for LV dimensions and function in children, reprinted with permission from reference [45]. Curves for boys are displayed in blue on the left, curves for girls are shown in pink on the right. Reference lines show the 3rd, 10th, 90th and 97th percentile. *LV* left ventricle, *ED* end diastolic, *ES* end systolic, *SV* stroke volume

**Fig. 6:**
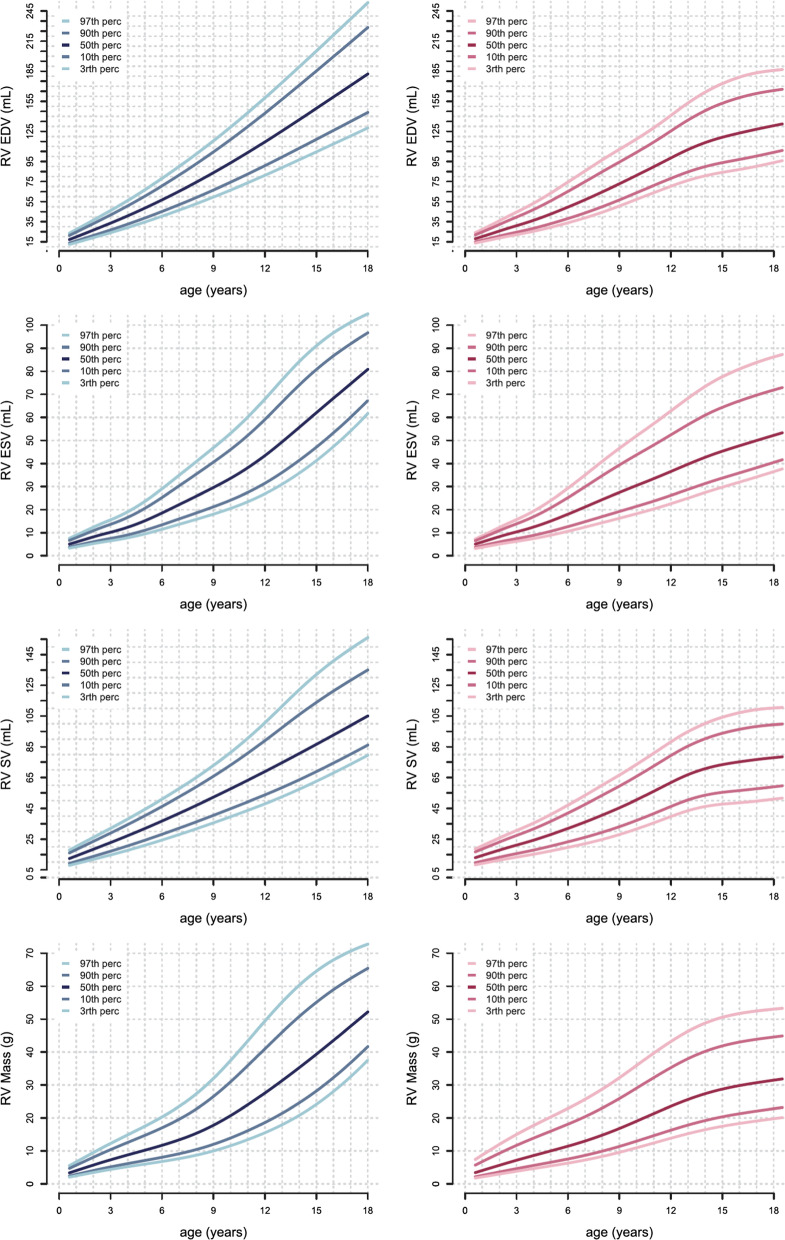
Reference curves for RV dimensions and function in children, reprinted with permission from reference [45]. Curves for boys are displayed in blue on the left, curves for girls are shown in pink on the right. Reference lines show the 3rd, 10th, 90th and 97th percentile. *LV* left ventricle, *ED* end diastolic, ES end systolic, *SV* stroke volume

Table 19 shows studies meeting inclusion criteria. The reference values for the LV and RV presented in the study by van der Ven [45] have been pooled from three previous studies [39–41], that have been reported separately in the previous version of our review [1]. Data are presented in percentile curves referred to age by using the LMS Method (Figs. 5, 6).

**Table 19:** References, normal dimensions of cardiac chambers in children

First author, year	CMR technique	n, male:female	Age range (years)
van der Ven, 2019 [45]	1.5 T, short axis bSSFP; dimensions of LV and RV; papillary muscles included in LV mass; RV mass measured at end-systole, major trabeculae included in RV mass when connected to the ventricular wall, trabeculae not connected to the wall included in RV volume	68:73	< 1–18
Sarikouch, 2011 [47]	1.5 T, axial bSSFP; pulmonary veins, superior and inferior vena cava and coronary sinus excluded, atrial appendages included from/in left and right atrial volume, respectively	56:59	4–20

*n* number of study subjects, *bSSFP* balanced steady-state free precession, *LV* left ventricular, *RV* right ventricular

### CMR analysis methods

For calculation of reference values from reference [45], the original bSSFP images (short axis) have been re-analysed by manual segmentation by one operator, after consensus on the segmentation rules was established within the group. These followed the standards proposed by SCMR [46], except for the trabeculations of the RV, required for calculating the RV mass. In the RV major trabeculae were included in the myocardium if they were visualized as being connected to the RV wall in more than 2 adjacent slices. Trabecular islands not connected to the wall were included in the blood pool [45].

## Left and right atrial dimensions and function in children

### CMR acquisition parameters

 LA and RA dimensions and function were evaluated using bSSFP technique in a single publication [47], (Table 19). Measurements were obtained on a stack of transverse cine bSSFP images with a slice thickness between 5 and 6 mm without interslice gap [47].

### CMR analysis methods

In [47], the pulmonary veins, the superior and inferior vena cava and the coronary sinus were excluded from the LA and RA volume, respectively, while the atrial appendages were included in the volume of the respective atrium. The maximal atrial volume was measured at ventricular end-systole and the minimal atrial volume at ventricular end-diastole.

### Demographic parameters

LA and RA volumes show an increase with age with a plateau after the age of 14 for girls only. Absolute and indexed volumes have been shown to be significantly greater for boys compared to girls (except for the indexed maximal volumes for both atria) [47].

### Studies included in this review

Sarikouch et al. evaluated atrial parameters of 115 healthy children (Table 19) [47] using bSSFP imaging. Data is presented as L, M, S values to enable calculation of the standard deviation score and in percentiles (Tables 20, 21).

**Table 20:** Normal left atrial and right atrial volume in boys; LMS parameters to calculate z-scores and percentiles relative to age according to reference [47]

Age^a^	Left atrium						Right atrium					
	LMS-parameters			Percentiles (ml/m^2^)			LMS-parameters			Percentiles (ml/m^2^)		
	L	M	S	P3	P50	P97	L	M	S	P3	P50	P97
6	1.378	36.715	0.263	14	37	55	1.806	33.342	0.191	20	39	68
7	1.378	38.610	0.246	17	39	56	1.806	48.385	0.203	22	43	71
8	1.378	40.291	0.229	20	40	57	1.806	51.247	0.205	24	47	73
9	1.378	41.762	0.212	22	42	58	1.806	51.742	0.205	26	49	74
10	1.378	43.375	0.197	25	43	59	1.806	52.579	0.204	28	52	75
11	1.378	45.120	0.183	27	45	61	1.806	54.891	0.200	30	54	76
12	1.378	46.671	0.171	29	47	62	1.806	56.348	0.197	32	57	77
13	1.378	47.784	0.161	31	48	62	1.806	57.830	0.193	33	59	78
14	1.378	48.331	0.152	33	48	62	1.806	59.473	0.188	34	61	79
15	1.378	48.581	0.142	34	49	62	1.806	61.042	0.181	35	63	80
16	1.378	49.112	0.131	36	49	61	1.806	63.114	0.171	37	65	81
17	1.378	50.353	0.120	38	50	62	1.806	64.322	0.161	38	67	82
18	1.378	52.583	0.111	40	53	64	1.806	66.227	0.145	40	69	84
19	1.378	55.860	0.103	44	56	67	1.806	72.157	0.110	43	71	85
20	1.378	59.928	0.097	48	60	71	1.806	77.498	0.064	45	72	86

LMS: L = Lambda (skewness of the distribution), M = Mu (median), S = Sigma (variance)

Standard deviation score (SDS) = [(X/M)L – 1]/(L*S), where X is the measured atrial volume in ml/m2 and L, M and S are the values interpolated for the child’s age; lower and upper limits correspond to a score of -2 and 2 and to the 3rd and 97th percentile, respectively

^a^Age in years

**Table 21:** Normal left atrial and right atrial volume in girls; LMS parameters to calculate z-scores and percentiles relative to age according to reference [47]

Age^a^	Left atrium						Right atrium					
	LMS-parameters			Percentiles (ml/m^2^)			LMS-parameters			Percentiles (ml/m^2^)		
	L	M	S	P3	P50	P97	L	M	S	P3	P50	P97
4	− 1.100	37.566	0.248	22	34	44	0.889	47.196	0.328	18	47	79
5	− 0.956	38.333	0.242	23	36	46	0.774	47.386	0.318	20	47	80
6	− 0.717	39.568	0.234	25	39	50	0.587	47.733	0.302	23	48	80
7	− 0.478	40.739	0.225	26	41	53	0.421	48.181	0.284	25	48	80
8	− 0.239	41.934	0.217	28	43	55	0.266	48.837	0.265	28	49	80
9	0.000	43.072	0.208	28	44	56	0.106	49.868	0.244	30	50	80
10	0.239	43.953	0.199	28	44	56	-0.033	51.098	0.221	33	51	80
11	0.478	44.548	0.191	29	44	57	-0.071	52.283	0.197	35	52	78
12	0.717	45.080	0.182	29	45	58	0.029	53.388	0.175	38	53	76
13	0.956	45.636	0.173	30	45	59	0.262	54.329	0.157	39	54	73
14	1.195	46.118	0.165	30	46	60	0.595	55.205	0.147	40	55	72
15	1.434	46.070	0.156	30	47	60	0.991	55.815	0.145	40	56	72
16	1.673	45.343	0.148	30	46	59	1.419	56.153	0.148	38	56	72
17	1.912	44.258	0.139	29	44	57	1.852	56.470	0.155	36	56	72
18	2.151	43.116	0.130	28	42	55	2.276	57.000	0.164	31	57	73

LMS, L = Lambda (skewness of the distribution), M = Mu (median), S = Sigma (variance)

Standard deviation score (SDS) = [(X/M)^L^ – 1] / (L*S), where X is the measured atrial volume in ml/m^2^ and L, M and S are the values interpolated for the child’s age; lower and upper limits correspond to a score of -2 and 2 and to the 3rd and 97th percentile, respectively

^a^Age in years

## Cardiac chamber size in the athlete

### CMR analysis methods

Methodologic considerations for CMR analysis are the same as for the non-athletes heart as described in the sections above. In both studies included in this review, papillary muscles and trabeculations were included in the ventricular volumes and excluded from LV and RV mass.

### Demographic parameters

Following the Mitchell classification, sports can be characterized as being high or low in dynamic (endurance, isotonic) versus static (strength/resistance, isometric) training and performance components [48]. Athletic competition can therefore be primarily (a) endurance (e.g. long distance running, swimming), (b) combined (e.g. rowers, cyclists) or (c) strength (e.g. body building and weight training). There are insufficient numbers of study subjects available in the literature to establish normative values for the strength category of athletes [49].

Cardiac chamber sizes may vary depending on the extent of exercise and training. One approach to classification is 9–18 h of training per week (regular athletes) vs > 18 h training per week (elite athletes) [50]. Adaptive changes to exercise are greater with higher exercise/training level [49].

Luijkx found a balanced increase of LV and RV chamber volume in relationship in the athlete heart [51]; a large meta-analysis of the literature had a similar conclusion [49]. RV and LV systolic function is commonly characterized by ejection fraction, but this parameter is known to show the most variation between observers. Nevertheless, RVEF and LVEF are > 50% in reports of the athlete’s heart by CMR [48].

The RV chamber volumes are greater in the athletes heart than in normal individuals [51]. The athlete’s RV volumes may exceed CMR criteria for abnormality in arrhythmogenic right ventricular cardiomyopathy (ARVC). However, RVEF is in the normal range of nonathletes even in the athlete heart (i.e. > 50%) whereas RVEF is abnormally low (≤ 45%) in ARVC.

### Studies included in this review

After elimination of redundant publications using the same study population and publications with > 40 athletes, there is one publication with data on the athlete’s heart by Prakken et al. (Table 22) [50]. This study was performed at 1.5 T and has sufficient description of CMR analysis technique to enable comparison (Tables 23, 24). Papillary muscles and trabeculation were included in ventricular volumes and excluded from myocardial mass. The study by Prakken et al. [50] specified levels of training (regular athletes 9–18 h/week; elite athletes > 18 h per week), both endurance and combined types of athletic participation were included. In contrast, Tahir et al. [52] identified athletes as those competing in triathlons (classified as ‘combined’ sport activity and training for more than 10 h per week) without further subcategorization. Although a smaller size cohort, the study by Tahir et al. may also be useful for the interested reader [52].

**Table 22:** Reference, cardiac chamber size in the athlete

First author, year	CMR technique	n, gender, sports intensity	Age range (years)
Prakken, 2010 [50]	1.5 T, short axis bSSFP, papillary muscles included in LV volume	83, male, regular athletes (9–18 h/week)	18–39
		46, male, elite athletes (> 18 h/week)	18–39
		60, female, regular athletes (9–18 h/week)	18–39
		33, female, elite athletes (> 18 h/week)	18–39
		56, male, non-athletes	18–39
		58, female, non-athletes	18–39

*n* number of study subjects, *bSSFP* balanced steady-state free precession

**Table 23:** Left ventricular parameters for adult athletes (papillary muscles included in LV volume) according to reference [50]

Parameter	Non-athletes [mean ± SD (LL–UL)^c^]		Regular athletes^a^ [mean ± SD (LL–UL)^c^]		Elite athletes^b^ [mean ± SD (LL–UL)^c^]	
	Men (n = 56)	Women (n = 58)	Men (n = 83)	Women (n = 60)	Men (n = 46)	Women (n = 33)
LVEDV (ml)	201 ± 33 (135–267)	156 ± 22 (112–200)	250 ± 32 (186–314)	194 ± 27 (140–248)	261 ± 39 (183–339)	199 ± 31 (137–261)
LVEDV/BSA (ml/m^2^)	101 ± 15 (71–131)	90 ± 11 (68–112)	123 ± 13 (97–149)	107 ± 14 (79–135)	129 ± 17 (95–163)	107 ± 14 (79–135)
LVESV (ml)	87 ± 19 (49–125)	65 ± 13 (39–91)	108 ± 20 (68–148)	86 ± 15 (56–116)	117 ± 24 (69–165)	85 ± 20 (45–125)
LVESV/BSA (ml/m^2^)	43 ± 10 (23–63)	37 ± 7 (23–51)	53 ± 9 (35–71)	48 ± 8 (32–64)	58 ± 11 (36–80)	46 ± 11 (24–68)
LVM (g)	95 ± 20 (55–135)	60 ± 11 (38–82)	125 ± 22 (81–169)	84 ± 17 (50–118)	139 ± 28 (83–195)	92 ± 15 (62–122)
LVM/BSA (g/m^2^)	48 ± 9 (30–66)	34 ± 6 (22–46)	62 ± 11 (40–84)	46 ± 9 (28–64)	69 ± 13 (43–95)	50 ± 8 (34–66)
LVEF (%)	57 ± 6 (45–69)	58 ± 5 (48–68)	57 ± 5 (47–67)	55 ± 4 (47–63)	55 ± 5 (45–65)	58 ± 7 (44–72)
max. IVS (mm)	10 ± 1 (8–12)	5 ± 1 (3–7)	11 ± 1 (9–13)	9 ± 1 (7–11)	11 ± 1 (9–13)	9 ± 1 (7–11)

*SD* standard deviation, *LL* lower limit, *UL* upper limit, *n* number of study subjects, *LV* left ventricular, *EDV* end-diastolic volume, *ESV* end-systolic volume, *EF* ejection fraction, *LVM* left ventricular mass, *max. IVS* maximal thickness of the interventricular septum, *BSA* body surface area

^a^9–18 h sports activity/week

^b^ > 18 h sports activity/week

^c^Calculated as mean ± 2*SD

**Table 24:** Right ventricular parameters for adult athletes (papillary muscles included in right ventricular volume) according to reference [50]

Parameter	Non-athletes [mean ± SD (LL–UL)^c^]		Regular athletes^a^ [mean ± SD (LL–UL)^c^]		Elite athletes^b^ [mean ± SD (LL–UL)^c^]	
	Men (n = 56)	Women (n = 58)	Men (n = 83)	Women (n = 60)	Men (n = 46)	Women (n = 33)
RVEDV (ml)	223 ± 40 (143–303)	166 ± 23 (120–212)	277 ± 36 (205–349)	209 ± 29 (151–267)	291 ± 48 (195–387)	219 ± 35 (149–289)
RVEDV/BSA (ml/m^2^)	111 ± 18 (75–147)	96 ± 12 (72–120)	136 ± 16 (104–168)	115 ± 15 (85–145)	144 ± 20 (104–184)	118 ± 17 (84–152)
RVESV (ml)	108 ± 24 (60–156)	75 ± 13 (49–101)	135 ± 25 (85–185)	102 ± 17 (68–136)	148 ± 30 (88–208)	103 ± 24 (55–151)
RVESV/BSA (ml/m^2^)	54 ± 12 (30–78)	43 ± 7 (29–57)	66 ± 12 (42–90)	57 ± 9 (39–75)	73 ± 13 (47–99)	56 ± 13 (30–82)
RVM (g)	23 ± 5 (13–33)	18 ± 4 (10–26)	29 ± 6 (17–41)	23 ± 4 (15–31)	30 ± 6 (18–42)	25 ± 5 (15–35)
RVM/BSA (g/m^2^)	12 ± 2 (8–16)	10 ± 2 (6–14)	14 ± 3 (8–20)	13 ± 2 (9–17)	15 ± 2 (11–19)	14 ± 3 (8–20)
RVEF (%)	52 ± 5 (42–62)	55 ± 5 (45–65)	51 ± 4 (43–59)	51 ± 4 (43–59)	50 ± 4 (42–58)	53 ± 7 (39–67)

*SD* standard deviation, *LL* lower limit, *UL* upper limit, *n* number of study subjects, *RV* right ventricular, *EDV* end-diastolic volume, *ESV* end-systolic volume, *EF* ejection fraction, *RVM* right ventricular mass, *BSA* body surface area

^a^9–18 h sports activity/week

^b^ > 18 h sports activity/week

^c^Calculated as mean ± 2*SD

**Table 25:** References, normal thickness of the compact left ventricular myocardium in the adult

First author, year	CMR technique	n, male:female	Age range (years)
Dawson, 2011 [56]	1.5 T, short axis bSSFP, 16 segments (apex excluded)	60:60	20–80
Kawel, 2012 [55]	1.5 T, short (16 segments, apex excluded) and long axis (12 segments) bSSFP	131:169	54–91
Le Ven, 2015 [14]	1.5 T, short axis bSSFP; 16 segments (apex excluded)	196:238	18–36
Yeon, 2015 [25]	1.5 T, short axis bSSFP; 2 segments (basal inferolateral and anteroseptal)	340:512	(men: 61 ± 8; women: 62 ± 9)^a^
Aquaro, 2017 [18]	1.5 T, short axis bSSFP; 2 segments (basal anterior septum, basal inferolateral wall)	173:135	15–80

*n* number of study subjects, *bSSFP* balanced steady-state free precession

^a^Age range not provided in original publication

Finally, one publication [49] presents a meta-analysis of the literature in an attempt to provide reference ranges. For the purposes of this review, that meta-analysis included multiple publications with overlapping/redundant study populations, small sample size (< 40 subjects in most studies) and did not take into account marked differences in analysis methods noted above. While useful to display overall trends in the literature for the athletes heart, the aforementioned meta-analysis was therefore not included in this study.

## Normal thickness of the compact left ventricular myocardium in adults

### CMR acquisition parameters

Normal values of the thickness of the compact LV myocardium have been shown to vary by type of pulse sequence (FGRE versus bSSFP) [53, 54]. For the purposes of this review, only bSSFP normal values are shown.

### CMR analysis methods

**Fig. 7:**
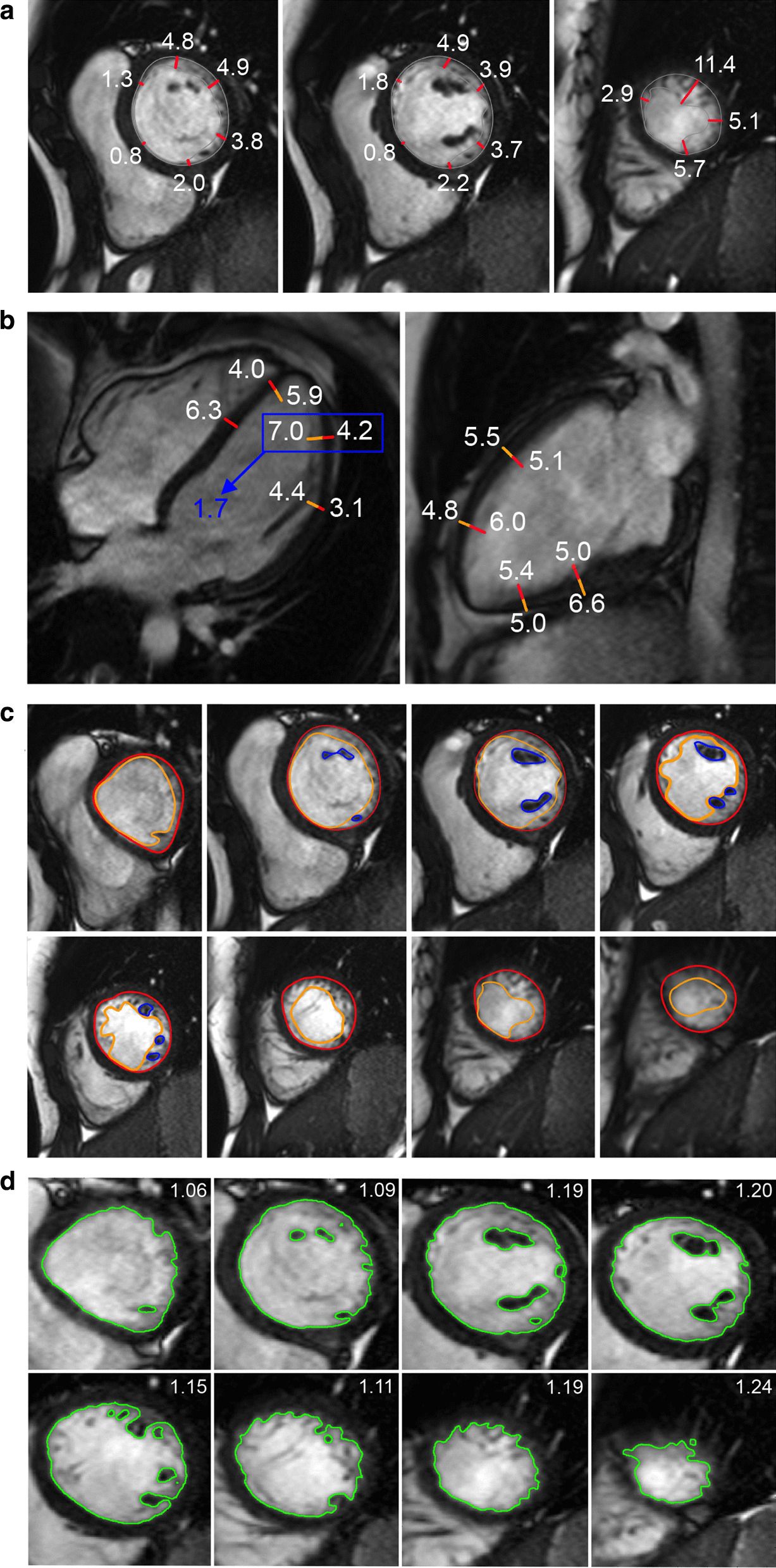
Example of measurement approaches for LV trabeculation. **a** End-diastolic thickness (in mm) of trabeculation according to the methodology in [56]: 3 slices representing base, mid and apex were selected from within the entire LV stack; trabeculated myocardial thickness was measured per slice; segment 17 excluded from analysis; authors do not clarify whether papillary muscles had been included or excluded from the trabecular measurement—in this reproduction we have excluded papillary muscles. **b** Maximal non-compacted (NC, red lines)/compacted (**c**, orange lines) wall thickness ratio according to the methodology in [61]: papillary muscles that were clearly observed as compact tubular structures were not included in the measurements; measurements in mm are shown in white and the maximal NC/C parameter highlighted in blue. **c** Trabeculation mass according to the methodology in [12]: the endocardial contour (red) was manually drawn; the trabecular contour (orange) was automatically segmented and papillary muscles (blue) that were included in the compact myocardial mass, were semi-automatically segmented; all slices of the LV short axis stack were analyzed. **d** Fractal dimension according to the methodology in [60]: using a semi-automatic level-set segmentation with bias field correction; all slices of the LV short axis stack are analyzed except for the apical slice; fractal dimensions per slice reported in the top right corner

In this review LV myocardial thickness refers to measurements of the thickness of the compact LV myocardium obtained at end-diastole (Fig. 7). Papillary muscles and trabeculations are excluded from measurement of the thickness of the compact LV myocardium.

Measures of LV myocardial thickness vary by the plane of acquisition (SAx versus long axis) [55]. Measurements obtained on long axis images at the basal and mid-cavity level have been shown to be significantly greater compared to measurements on corresponding SAx images, whereas measurements obtained at the apical level of long axis images are significantly lower compared to SAx images.

### Demographic parameters

LV myocardial thickness is greater in men than women [14, 18, 25, 55, 56]. There are also small differences in LV myocardial thickness in relationship to ethnicity and body size, but these variations are not likely to have clinical significance [55]. Regarding age, one study of 120 healthy subjects age 20–80 years reported an increase in myocardial thickness with age—starting after the fourth decade [56]. In the study by Kawel el al. of 300 normal individuals without hypertension, smoking history or diabetes, there was no statistically significant difference in LV myocardial thickness with age [55].

### Studies included in this review

There are five publications of a systematic analysis of LV myocardial thickness based on bSSFP imaging at 1.5 T with a sample size > 40 healthy subjects per gender and a detailed description of the measurement technique (Table 25). Dawson et al. and Le Ven et al. published measurements for all 16 segments (apex excluded) obtained on short axis images (Table 26) [14, 56]. Kawel et al. published normal values of LV myocardial thickness for long and SAx imaging for 12 and 16 segments, respectively (Tables 26, 27) [55]. Yeon et al. and Aquaro et al. obtained measurements for only two myocardial segments on SAx images (Table 26) [18, 25].

**Table 26:** Normal left ventricular myocardial thickness (in mm) in the adult measured on short axis images for men and women

Level	Segment	Men				Women			
		n	Mean_p_	SD_p_	LL–UL^c^	n	Mean_p_	SD_p_	LL–UL^c^
Basal	1^a^	387	7.8	1.3	5–10	467	6.4	1.1	4–9
	2^b^	900	9.0	1.4	6–12	1114	7.6	1.2	5–10
	3^a^	387	8.8	1.2	6–11	467	7.3	1.0	5–9
	4^a^	387	7.9	1.2	6–10	467	6.4	1.0	4–8
	5^b^	900	7.7	1.2	5–10	1114	6.3	1.1	4–9
	6^a^	387	7.5	1.2	5–10	467	6.1	1.0	4–8
Mid-cavity	7^a^	387	6.7	1.2	4–9	467	5.6	1.0	4–8
	8^a^	387	7.4	1.3	5–10	467	6.1	1.0	4–8
	9^a^	387	7.9	1.2	6–10	467	6.6	1.0	5–9
	10^a^	387	7.0	1.2	5–9	467	5.8	1.0	4–8
	11^a^	387	6.5	1.4	4–9	467	5.3	1.0	3–7
	12^a^	387	6.6	1.2	4–9	467	5.5	1.1	4–8
Apical	13^a^	387	6.5	1.2	4–9	467	5.9	1.3	3–9
	14^a^	387	6.8	1.3	4–9	467	5.8	1.1	4–8
	15^a^	387	6.1	1.1	4–8	467	5.2	1.0	3–7
	16^a^	387	6.2	1.1	4–8	467	5.6	1.0	4–8

Segments: 1: basal anterior, 2: basal anteroseptal, 3: basal inferoseptal, 4: basal inferior, 5: basal inferolateral, 6: basal anterolateral, 7: mid anterior, 8: mid anteroseptal, 9: mid inferoseptal, 10: mid inferior, 11: mid inferolateral, 12: mid anterolateral, 13: apical anterior, 14: apical septal, 15: apical inferior, 16: apical lateral

*n* number of study subjects included in the weighted mean values, *mean*_*p*_ pooled weighted mean, *SD*_*p*_ pooled standard deviation, *LL* lower limits, *UL* upper limits

^a^Pooled weighted values from references [14, 55, 56]

^b^Pooled weighted values from references [14, 18, 25, 55, 56]

^c^Calculated as mean_p_ ± 2*SD_p_

**Table 27:** Normal left ventricular myocardial thickness (in mm) in the adult measured on long axis images for men and women according to reference [55]

Level	Region	Men (n = 131)			Women (n = 169)		
		Mean	SD	LL–UL^a^	Mean	SD	LL–UL^a^
Basal	Anterior	8.2	1.3	6–11	7	1.1	5–9
	Inferior	8.2	1.3	6–10	6.7	1.1	5–9
	Septal	9.1	1.3	7–12	7.3	1.1	5–10
	Lateral	7.6	1.3	5–10	6	1.1	4–8
	Mean	8.3	1.0	6–10	6.8	0.9	5–9
Mid-cavity	Anterior	6	1.3	3–9	4.9	1.1	3–7
	Inferior	7.7	1.3	5–10	6.5	1.1	4–9
	Septal	8.3	1.3	6–11	6.8	1.1	5–9
	Lateral	6.6	1.3	4–9	5.3	1.1	3–8
	Mean	7.2	1.0	5–9	6	1	4–8
Apical	Anterior	5.1	1.3	3–8	4.2	1.1	2–6
	Inferior	5.8	1.3	3–8	5	1.1	3–7
	Septal	5.8	1.3	3–8	5	1.1	3–7
	Lateral	5.5	1.3	3–8	4.6	1.1	2–7
	Mean	5.6	1.0	4–8	4.7	0.9	3–7

*n* number of study subjects, *SD* standard deviation, *LL* lower limits, *UL* upper limits

^a^Calculated as mean ± 2*SD

## Normal values of left ventricular trabeculation

### CMR acquisition parameters

CMR methods used to assess LV trabeculation (Table 28) are based on the bSSFP technique to leverage on the blood-myocardial contrast it provides. The key methods are illustrated in Fig. 7.

**Table 28:** References, normal thickness, mass, ratios and fractal dimension of the left ventricular trabeculated (non-compacted) myocardium in the adult

First author, year	CMR technique	n, male:female	Age range (years)
**Trabeculation thickness (thickness of the trabeculated [non-compacted] LV myocardium)**			
Dawson, 2011 [56]	1.5 T, short axis bSSFP, maximal thickness per segment at diastole and systole	60:60	20–80
**NC/C thickness ratio (thickness of trabeculated [non-compacted] LV myocardium/ thickness of compact LV myocardium)**			
Dawson, 2011 [56]	1.5 T, short axis bSSFP, NC/C thickness ratio per segment measured manually at the “peak of the most prominent trabeculae in each segment” at diastole and systole	60:60	20–80
Kawel, 2012 [61]	1.5 T, long axis bSSFP at diastole, maximal NC/C thickness ratio of 12 segments	192:175	54–91
Captur, 2013 [59]	1.5 T, long axis bSSFP at diastole, maximal NC/C thickness ratio of 16 segments	40 (total)*	18–85
Tizón-Marcos, 2014 [65]	1.5 T, long- and short axis bSSFP, mean NC/C thickness ratio per segment measured semi-automatically by the centerline method (average of 20–30 chords/segment) at diastole and systole	45:55	18–35
Amzulescu, 2015 [58]	1.5 T and 3 T, long axis bSSFP at diastole, maximal NC/C thickness ratio of 16 segments	22:26	(60 ± 10)**
André, 2015 [64]	1.5T, long axis bSSFP at diastole, maximal NC/C thickness ratio of 16 segments	58:59	20– > 50
**Trabeculation mass (mass of the trabeculated [non-compacted] LV myocardium)**			
Bentatou, 2018 [12]	1.5 T, short axis bSSFP at diastole, papillary muscles and blood between trabeculae excluded	70:70	20–69
**Trabeculation volume (volume of the trabeculated [non-compacted] LV myocardium)**			
André, 2015 [64]	1.5 T, short axis bSSFP, blood between trabeculae included, papillary muscles excluded	58:59	20– > 50
**NC/C mass ratio (mass of trabeculated [non-compacted] LV myocardium/ mass of compact LV myocardium)**			
Amzulescu, 2015 [58]	1.5 T and 3 T, short axis bSSFP at diastole, mass of trabeculated myocardium includes trabeculae and blood between trabeculae, papillary muscles excluded from trabeculated and compact mass	22:26	(60 ± 10)^b^
Bentatou, 2018 [12]	1.5 T, short axis bSSFP at diastole, blood between trabeculae excluded from mass of trabeculated myocardium, papillary muscles included in mass of compact myocardium	70:70	20–69
**NC/TM (mass of trabeculated [non-compacted] LV myocardium/ total LV myocardial mass [trabeculated + compact LV myocardial mass])**			
Captur, 2013 [59]	1.5 T, short axis bSSFP at diastole, mass of trabeculated myocardium includes trabeculae and blood between trabeculae, papillary muscles included in mass of compact myocardium	40 (total)^a^	18–85
**Fractal dimension (fractal complexity of LV trabeculated [non-compacted] myocardium)**			
Captur, 2013 [59]	1.5 T, short axis bSSFP at diastole, papillary muscles included in the endocardial complexity	51:54 (75 white, 30 black)	18–85
Captur 2015 [62]	1.5 T, short axis bSSFP at diastole, papillary muscles included in the endocardial complexity	279:325	46–91
Cai, 2017 [66]	3 T, short axis bSSFP at diastole, papillary muscles included in the endocardial complexity	91:89	20–69

*n* number of study subjects, *LV* left ventricular, *bSSFP* balanced steady-state free precession

^a^Male:female ratio not provided in original publication

^b^Age range not provided in original publication

### CMR analysis methods

No uniformly accepted convention has been used for analyzing trabeculation. At least seven different measurement approaches have been described (Table 28). Principally these methods measure trabeculation in the LV either in terms of the trabeculated layer’s thickness, mass, volume, or fractal complexity, with or without adjusting for the thickness, mass or volume of the adjacent compacted myocardium. Tables of normal values for trabeculation should specify the phase of the cardiac cycle in which measurements were taken together with imaging planes used. When reporting trabeculation mass, volume or fractal complexity, tables should specify whether papillary muscles were included or excluded in the trabecular assessment. Where semi-automated segmentation of trabecular contours is undertaken, the type of algorithm used may impact subsequent results so the methods must specify the algorithm in detail [57].

Table 29 provides normal adult values for thickness of the trabeculated LV myocardium, on a segment-by-segment basis. Table 30 provides normal values for mass and volume of trabeculation. Trabeculation mass ratio has additionally been reported [12, 58, 59] but measurement heterogeneity across studies, with respect to handling of the blood pool between trabeculations and inclusion/exclusion of papillary muscles, has led to differing definitions and no consensus normal values.

**Table 29:** Normal thickness of the trabeculated (non-compacted) left ventricular myocardium on short axis at end-diastole (in mm) in the adult according to [56]

Level	Segment	Mean (median)	SD (IQR)
Basal	1	3.0	0, 4.6
	2	0	
	3	0	
	4	0	
	5	0	0, 3.9
	6	0	0, 4.1
Mid-cavity	7	5.6	2.8
	8	0	
	9	0	
	10	0	0, 2.1
	11	4.2	2.5
	12	4.4	2.7
Apical	13	5.6	2.7
	14	0	
	15	0	0, 4.5
	16	7.1	2.4

According to the original publication (n = 120), data are presented as mean ± SD for normally distributed variables and as median (first, third interquartile ranges) for nonparametric variables; Segments: 1 = basal anterior, 2 = basal anteroseptal, 3 = basal inferoseptal, 4 = basal inferior, 5 = basal inferolateral, 6 = basal anterolateral, 7 = mid anterior, 8 = mid anteroseptal, 9 = mid inferoseptal, 10 = mid inferior, 11 = mid inferolateral, 12 = mid anterolateral, 13 = apical anterior, 14 = apical septal, 15 = apical inferior, 16 = apical lateral.

**Table 30:** Normal values for mass and volume of trabeculated (non-compacted) left ventricular myocardium in the adult measured on short axis images

Parameter	Technique	Men			Women		
		n	Mean	SD	n	Mean	SD
Trabeculation mass (mass of the trabeculated [non-compacted] LV myocardium) per BSA (g/m2) from ref [12]	Papillary muscles and blood between trabeculae excluded	70	5.4	2.3	70	4.0	2.3
Trabeculation volume (volume of the trabeculated [non-compacted] LV myocardium) per BSA (ml/m2) from ref [64]	Blood between trabeculae included, papillary muscles excluded	58	43.1	8.7	59	36.1	5.2

*n* number of study subjects, *SD* standard deviation, *BSA* body surface area

Tables 31 and 32 provide normal values for LV trabeculation measured as a fractal dimension. Four fractal parameters for quantifying LV trabeculation [59] include global LV, maximal basal, maximal mid and maximal apical fractal dimension. To derive the global LV fractal dimension, the fractal dimensions from each slice in the LV stack (Fig. 7d) were averaged; to derive local fractal characteristics, the maximal fractal dimension in the basal, mid and apical thirds of the left ventricle were recorded [59].

**Table 31:** Normal values for the fractal dimension (FD) (unitless) of left ventricular trabeculation in the adult for different ethnicities

Parameter	Ethnicity	n	Mean	SD
Global FD from ref [59]	Black	30	1.246	0.005
Maximal apical FD^a^ from ref [59]	Black	30	1.235	0.03
Global FD from ref [59]	White	75	1.228	0.002
Maximal apical FD^a^ from ref [59]	White	75	1.253	0.025
Global FD from ref [66]	Singaporean Chinese	180	1.205	0.031
Maximal apical FD^b^ from ref [66]	Singaporean Chinese	180	0.278	0.045

*n* number of study subjects, *SD* standard deviation

^a^Measured for the apical third of the left ventricle

^b^Measured for the apical half of the left ventricle

**Table 32:** Normal values for the fractal dimension (FD) (unitless) of left ventricular trabeculation in the adult stratified by sex and body mass index (BMI) according to reference [62]

Parameter	BMI ≥ 30 kg/m^2^ (mean ± SD)			BMI ≥ 25 to < 30 kg/m^2^ (mean ± SD)			BMI < 25 kg/m^2^ (mean ± SD)		
	All (n = 163)	Men (n = 71)	Women (n = 92)	All (n = 206)	Men (n = 108)	Women (n = 98)	All (n = 235)	Men (n = 100)	Women (n = 135)
Max. apical FD^a^	1.203 ± 0.06	1.212 ± 0.07	1.196 ± 0.06	1.194 ± 0.06	1.197 ± 0.05	1.190 ± 0.07	1.169 ± 0.07	1.177 ± 0.06	1.162 ± 0.05

*n* number of study subjects, *BMI* body mass index, *SD* standard deviation, *Max*. maximal

^a^Measured for the apical half of the left ventricle

Normal values by this approach for global LV and maximal apical fractal dimension are presented in Table 31. Methodological developments for fractal analysis of the left ventricle are ongoing [60].

### Demographic parameters

In the largest published reference cohort (n = 323) [61], there was no relationship between maximal non-compacted (NC)/compacted (C) wall thickness ratio and age, gender, race/ethnicity, height or weight.

For segment-by-segment (whole-heart) NC/C ratio [56], there was also no significant difference between genders, but age-related differences were present: the thickness of the trabeculated myocardium generally increased until the 3rd decade and subsequently decreased. This trend was significant in the anterior (1, 7, 13) and apical inferior segments, but not in the remainder of segments [56].

Using the fractal dimension, ethnicity was shown to influence LV trabeculation parameters, with greater endocardial complexity (i.e. higher fractal dimension) demonstrated in healthy blacks as opposed to healthy whites, and greater complexity demonstrated in Whites, African American and Hispanics compared to Chinese Americans [62].

### Studies included in this review

For the purpose of this review, only cohorts of 40 or more normal subjects using bSSFP CMR technique have been included. Data from population-based studies where exclusions for comorbidities was undertaken have also been included [61, 62]. The majority of reported normal values were derived at 1.5 T although a few 3 T studies have also been undertaken (see Table 28). Inclusion criteria for reported tables included a full description of the subject cohort (including the analysis methods used), age and gender of subjects. One study evaluated elite male athletes which was not deemed to be representative of the average population and was therefore not included in this review [63].

The caliper-based linear measurement of thickness of trabeculation [61] has progressively evolved into more complex metrics: the *maximal* NC/C thickness ratio has been measured by at least four groups [58, 59, 61, 64] but reported normal values were too discordant for calculation of weighted means in this review (thus not shown in Table 29). The inter-study discordance of maximal NC/C parameters may stem from the subjective selection by readers of the visually most trabeculated segment/s for analysis (Fig. 7b). The largest of these studies, which also included reproducibility assessment, reported median values for normal adult maximal NC/C thickness of 2.2 [5th and 95th percentile: 1.0, 4.6] [61]. Other studies opted for a more systematic segment-by-segment analysis of thickness of trabeculation but still methodologies differed: Dawson et al. [56]. measured the maximal thickness of trabeculated myocardium per segment (Fig. 7a), whereas Tizon [65] measured the average of 20–30 measurements of the thickness of trabeculation per segment, with consequently different results.

## Cardiac valves and quantification of flow

### CMR acquisition parameters

Prospectively and retrospectively electrocardiogram (ECG)-gated phase contrast (PC) CMR sequences are widely available. Prospectively-gated sequences use arrhythmia rejection and may be performed in a breath hold. Retrospectively gated techniques are mainly performed during free-breathing, often with higher spatial and temporal resolution compared to the breath hold techniques [67]. Four-dimensional flow-sensitive (4D Flow) PC CMR techniques have shown promising initial results, but 2D PC flow techniques remains the most commonly used approach in daily clinical practice [68]. In addition to PC-CMR, valve planimetry—using ECG-gated bSSFP CMR—can also be used to estimate stenosis or insufficiencies with good correlation to echocardiographic measurements [69].

Measurements of flow are most precise when (a) the imaging plane is positioned perpendicular to the vessel of interest and (b) the velocity encoded gradient echo (V_enc_) is encoded in a through plane direction [70]. The slice thickness should be ≤ 7 mm to minimize partial volume effects. Compared to aortic or pulmonary artery flow evaluation, quantification of mitral or tricuspid valves is more challenging using PC-CMR due to through plane motion during the cardiac cycle [71].

The flow encoding velocity (V_enc_) should be chosen close to the maximum expected flow velocity of the examined vessel for precise measurements. Setting the V_enc_ below the peak velocity results in aliasing. For the normal aorta and main pulmonary artery, maximum velocities usually do not exceed 150 and 90 cm/s, respectively.

Adequate temporal resolution is necessary to avoid temporal flow averaging, especially for the evaluation of short, fast, and turbulent jets within a vessel (e.g. aortic stenosis). For clinical routine, 25–30 ms temporal resolution is sufficient. The minimum required spatial resolution is less than one third of the vessel diameter to avoid partial volume effects with the adjacent vessel wall and surrounding stationary tissues for small arteries [70].

### CMR analysis methods

For data analysis, dedicated flow software should be used. Most of the currently available flow software tools offer semi-automatic vessel contouring, which needs to be carefully checked by the examiner.

The modified Bernoulli equation (∆P = 4 × V_max_^2^) is commonly used for calculation of pressure gradients using PC-CMR across the pulmonary or aortic valve [72, 73].

Velocity measurements of valvular stenosis with high jet velocities may be inaccurate due to (A) partial volume effects in case of a small jet width and (B) limited temporal resolution compared to the high velocity of the jet. Measurements are further affected by signal loss due to the high velocity that may lead to phase shift errors and dephasing. Misalignment of the slice relative to the direction of the jet may also lead to an underestimation of the peak velocity [74].

Mitral valve flow velocities and deceleration times can be quantified for assessment of LV diastolic function, in a manner analogous to that used with transthoracic echocardiography (TTE). 2D PC derived trans-mitral flow velocities and deceleration times are strongly correlated with TTE derived parameters, but with a systematic underestimation [75].

### Demographic parameters

To our knowledge, no comprehensive studies have been performed to investigate the association between age, gender and ethnicity and valvular flow or valve planimetry in normal healthy subjects based on PC-CMR. Two recent studies using 4D Flow CMR investigated the relationship of aortic flow velocity with age and gender, respectively [76, 77]. Callaghan et al. [76] compared measurements of mean peak systolic velocity obtained in the ascending aorta between 3 age groups and found a significant decrease with age. Garcia et al. [77] showed the mean aortic valve peak velocity was higher with greater age. In the study by Garcia et al. the differences in peak systolic velocity with gender were small and likely not clinically relevant [77].

### Studies included in this review

There is good agreement between PC-CMR, bSSFP CMR planimetry, and echocardiography measurements. American Heart Association (AHA) criteria for grading valve stenosis or insufficiency is suggested [78, 79] (Table 33). To our knowledge, there is no publication from a large study of normal reference values of trans-valvular flow and valve planimetry based on PC-CMR measurements.

**Table 33:** Stages of valvular heart disease in the adult. adapted from echocardiography according to references [78, 79]

Valve disease	Parameter	Stage	
		Progressive	Severe
Aortic stenosis	Maximum velocity (m/s)	Mild: 2.0–2.9 Moderate: 3.0–3.9	Severe: ≥ 4 Very severe: ≥ 5 Low-flow/low-gradient: < 4 m/s (at rest)
	Orifice area (cm^2^)		≤ 1.0
	Orifice area /BSA (cm^2^/m^2^)		≤ 0.6
Aortic regurgitation	Regurgitant volume (ml/beat)	Mild: < 30 Moderate: 30–59	≥ 60
	Regurgitant fraction (%)	Mild: < 30 Moderate: 30–49	≥ 50
	Effective regurgitant orifice (cm^2^)	Mild: < 0.10 Moderate 0.10–0.29	≥ 0.30
Mitral stenosis	Transmitral flow velocity (m/s)	Increased	
	Orifice area (cm^2^)	> 1.5	Severe: ≤ 1.5 Very severe: ≤ 1.0
Primary mitral regurgitation	Regurgitant volume (ml/beat)	< 60	≥ 60
	Regurgitant fraction (%)	< 50	≥ 50
	Effective regurgitant orifice (cm^2^)	< 0.40	≥ 0.40
Secondary mitral regurgitation	Regurgitant volume (ml/beat)	< 60	≥ 60
	Regurgitant fraction (%)	< 50	≥ 50
	Effective regurgitant orifice (cm^2^)	< 0.40	≥ 0.40
Pulmonic stenosis	Peak velocity (m/s)		> 4
Tricuspid stenosis	Orifice area (cm^2^)		< 1.0

*BSA* body surface area

Mitral valve flow parameters for determination of diastolic LV function are shown in Table 34.

**Table 34:** Mitral valve flow for determination of diastolic left ventricular function according to reference [80]

Parameter	Normal	Type 1 (Impaired relaxation)	Type 2 (Pseudonormal)	Type 3 (Restrictive, partially reversible)	Type 3 (Restrictive, fixed)
MDT (ms)	150–220	Increased	Normal	Decreased	Decreased
E/A ratio	1–2	< 1	1–2	> 2	> 2

*MDT* mitral deceleration time, *E/A ratio* ratio of the mitral early (E) and atrial (A) components of the mitral inflow velocity profile

**Fig. 8:**
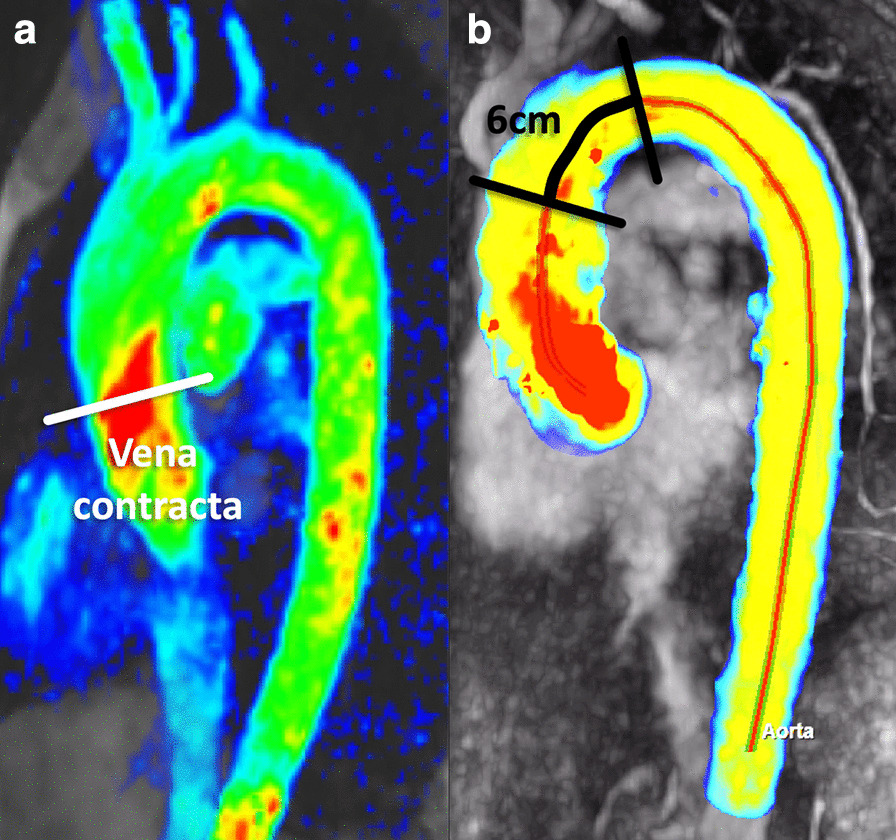
Images of a 4D flow sequence illustrating sites of measurement of peak systolic velocity. According to reference [77] measurements were obtained where the transvalvular velocity reaches its maximum during peak systole (vena contracta region) (**a**). In reference [76] peak systolic velocity was obtained in the ascending aorta 6 cm proximal from the most cranial point of the aortic arch centerline (**b**)

Garcia, et al. [77] and Callaghan, et al. [76] have reported normal thoracic aorta flow parameters using 4D Flow CMR. Amongst other parameters, Garcia obtained measurements of peak systolic velocity where the transvalvular velocity reaches its maximum during peak systole (vena contracta region) (Fig. 8a) while Callaghan acquired measurement 6 cm proximal from the most cranial point of the aortic arch centerline in the ascending aorta (Fig. 8b). Normal values of peak aortic velocity are given in Tables 35 and 36.

**Table 35:** Normal mean peak systolic velocity of the ascending aorta by 4D-flow for different age groups according to reference [76]

Parameter	18–33 years				34–60 years				> 60 years			
	n	Mean	SD	LL–UL^a^	n	Mean	SD	LL–UL^a^	n	Mean	SD	LL–UL^a^
Velocity (cm/s)	64	66	15	36–96	116	51	13	25–77	67	35	12	11–59

Measured 6 cm proximal from the most cranial point of the aortic arch centerline (Fig. 8b)

*n* number of study subjects, *SD* standard deviation, *LL* lower limit, *UL* upper limit

^a^Calculated as mean ± 2*SD

**Table 36:** Normal mean aortic valve peak velocity by 4D-flow CMR for men and women according to reference [77]

Parameter	Men				Women			
	n	Mean	SD	LL–UL^a^	n	mean	SD	LL–UL^a^
Velocity (m/s)	57	1.3	0.3	0.8–1.8	41	1.2	0.2	0.8–1.6

Measured where the transvalvular velocity reaches its maximum during peak systole (Fig. 8a)

*n* number of study subjects, *SD* standard deviation, *LL* lower limit, *UL* upper limit

^a^Calculated as mean ± 2*SD

## Normal aortic dimensions in the adult

### CMR acquisition parameters

Three-dimensional contrast enhanced CMR angiography (CMRA) has gained broad acceptance and is widely used for the assessment and follow-up of thoracic aortic diameters in the clinical setting. The multi-planar reformation of CMRA images leads to an accurate measurement perpendicular to the lumen of the vessel. However, motion caused by pulsation leads to substantial blurring of the vessel contour at the level of the aortic root, hampering accurate diameter measurements [81]. The need of a contrast injection is another limitation for the use of this technique, particularly in patients who need multiple follow up examinations and in population based study settings [82]. Alternatively non-contrast techniques such as an ECG- and respiratory-gated gadolinium-enhanced CMRA or 3D bSSFP sequence can be applied, enabling accurate measurements of aortic diameters including the aortic root [82]. However, due to the long acquisition times or lack of sequence availability, these methods may not be widely applied [81]. The magnitude image of PC CMR has also been used to measure diameters of the aorta [83]. Black blood techniques are used for a more detailed assessment of the aortic wall [84].

In 2D acquisitions, the imaging plane needs to be acquired correctly at the time of the scan; thus, any alterations in the imaging plane due to breath-holding or patient motion will result in variability of measurements. Through plane motion during the cardiac cycle can be minimized with ECG gating [82].

Potthast and colleagues compared the diameter of the ascending aorta obtained by different CMR sequences to ECG-triggered computed tomography angiography (CTA) as the standard of reference. They reported that ECG-gated navigator triggered 3D bSSFP sequence showed the best agreement with CTA [82].

### CMR analysis methods

**Fig. 9:**
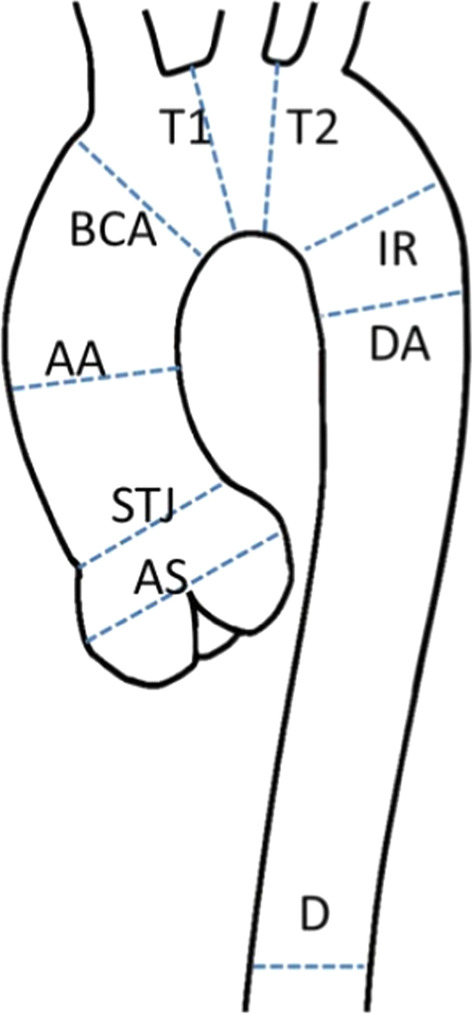
Sites of measurement of the thoracic aorta. *AS* aortic sinus, *STJ* sinotubular junction, *AA* ascending aorta, *BCA* proximal to the origin of the brachiocephalic artery, *T1* between the origin of the brachiocephalic artery and the left common carotid artery, *T2* between the origin of the left common carotid artery and the left subclavian artery, *IR* isthmic region, *DA* descending aorta, *D* thoracoabdominal aorta at the level of the diaphragm

Beside the sequence type, imaging plane and cardiac phase (systole versus diastole), it is important to identify the anatomic locations of diameter measurements of the thoracic aorta (Fig. 9).

**Fig. 10:**
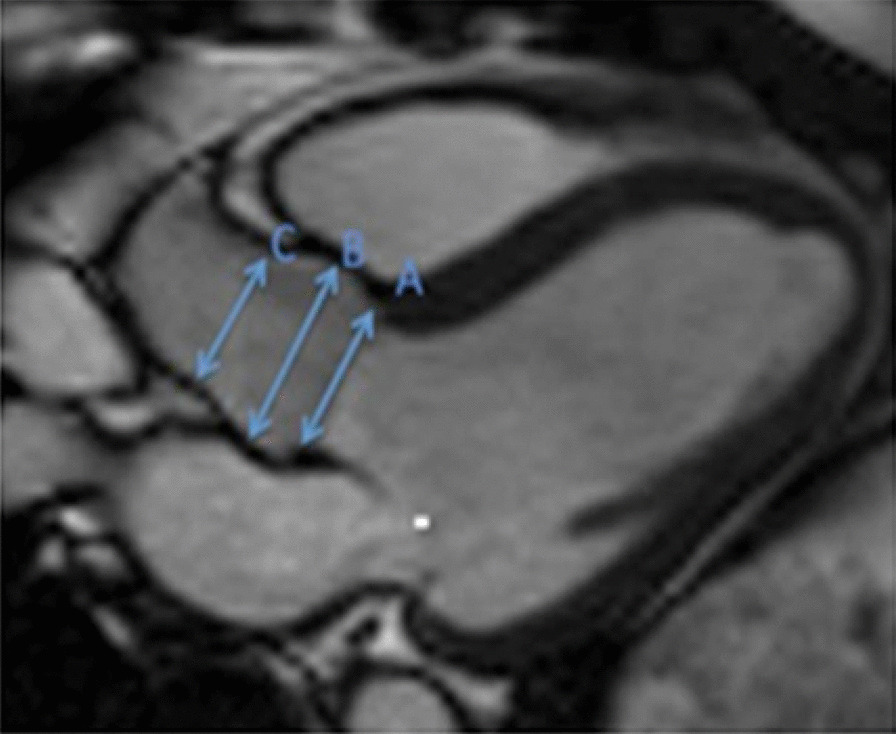
Measurements of luminal diameters of the aortic annulus (**a**), the aortic sinus (**b**) and the sinotubular junction (**c**) obtained on a steady-state free precession left ventricular outflow tract view at diastole according to reference [86]

**Fig. 11:**
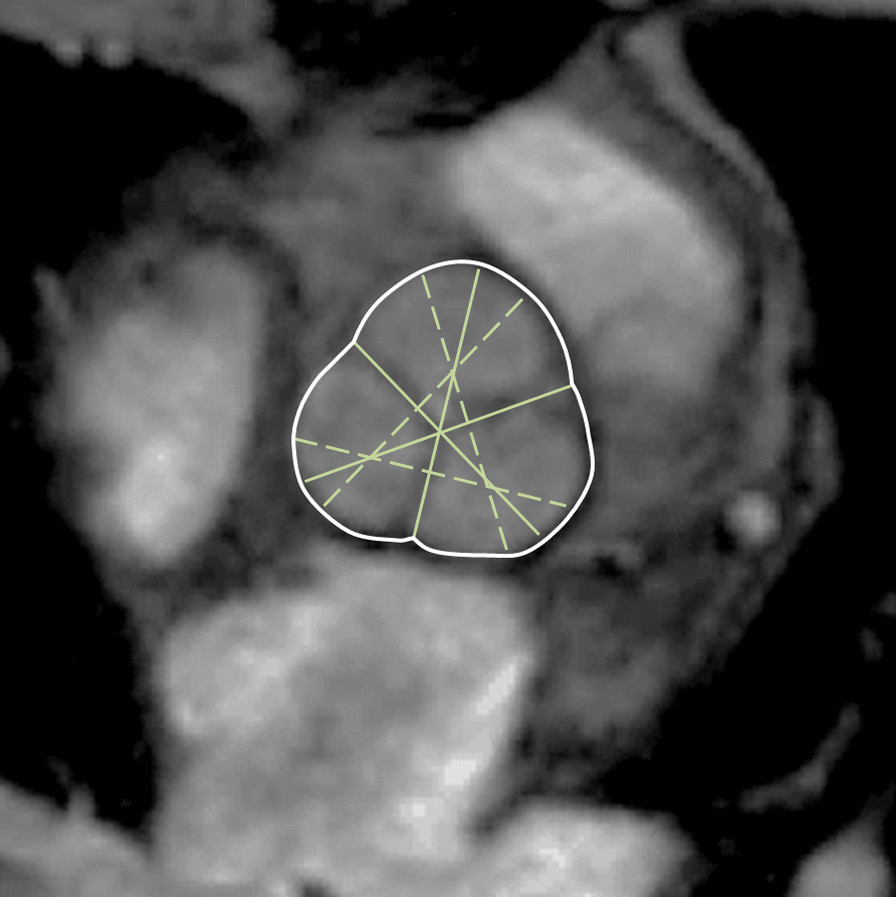
Cusp-commissure (continuous lines) and cusp-cusp (dashed-lines) measurements at the level of the aortic sinus according to reference [85]

The sagittal oblique view of the LV outflow tract was used for measuring diameter at the level of the aortic annulus, the aortic sinus, and the sinotubular junction (Fig. 10) [11, 85, 86]. Axial cross sectional images at predefined anatomic levels were used for measuring the ascending and descending aorta [86] as well as cusp-commissure and cusp-cusp diameters at the level of the aortic sinus [85] (Fig. 11).

Luminal or outer to outer diameter of the aorta may be measured. The current SCMR guidelines on image post-processing recommend measurement of the outer contour in dilatation while measurements of the inner contour should be obtained in the setting of stenosis [9]. In the tables below, the method is specified.

### Demographic parameters

In the MESA, a large population based study, the diameter of the ascending aorta has been shown to be larger in men compared to women even after adjustment for BSA [83]. In a publication by Le et al., however, the gender difference in diameters did not persist after normalization to BSA [11].

Several studies have shown an increase in aortic diameter with age [11, 83, 85, 86]. The association of age with aortic diameter was more marked in the ascending aorta compared to the descending thoracic and abdominal aorta, respectively [87, 88]. Further, age-related changes of the geometry of the thoracic aorta have been described. Age-related changes include increasing length of the ascending aorta and decreasing curvature of the aortic arch [89, 90].

In the MESA study, there were small differences in normal aortic diameter for Chinese and African American participants compared to Caucasians. These differences were small however relative to measurement error and reproducibility and therefore may not be clinically relevant [83].

### Studies included in this review

Studies with normal values of aortic diameters based on measurements obtained in studies with 40 or more healthy subjects per gender have been included in this review (Table 37). There are five major publications regarding CMR-based measurements of the thoracic aorta in adults [11, 83–86]. There is substantial difference between the studies with respect to CMR sequences (cine bSSFP, PC CMRA and 3D-T1-black blood volume isotropic turbo spin echo acquisition), acquisition/ measurement plane (cross sectional versus LV outflow tract view), measurement technique (luminal versus total diameter and area, respectively) and measurement sites of the aorta. Therefore, results of most studies are presented separately (Tables 38, 39, 40, 41). Details of image acquisition and measurement technique of each study can be found in Table 37 and are described in the footnote of each table.

**Table 37:** References, normal aortic diameters, area and wall thickness in adults

First author, year	CMR technique	n, male:female	Age range (years)
Burman, 2008 [85]	1.5 T, cine bSSFP, luminal diameter at systole and diastole, average of 3 cusp-commissure and 3 cusp-cusp diameters, respectively on cross-sectional images of the aortic sinus and diameter of the aortic sinus on the sagittal LVOT plane	60:60	20–80
Davis, 2014 [86]	1.5 T, cine bSSFP, maximal luminal diameter at diastole, diameters calculated based on measurements of the area at 3 levels (ascending aorta, proximal and distal descending aorta) of the aorta on cross-sectional images and diameters at 3 levels (annulus, sinus, sinotubular junction) of the aortic root measured on the sagittal LVOT plane	208:239	19–70
Turkbey, 2014 [83]	1.5 T, luminal diameter of the ascending aorta measured on the magnitude image of a phase contrast sequence	770:842	45–84
Eikendal, 2016 [84]	3 T, fat suppressed 3D-T1-black blood VISTA acquired sagittal of the descending aorta, luminal and total vessel diameter and area, calculated average diameter, luminal and total vessel area, vessel wall area and thickness of the proximal to distal descending aorta after manual tracing of the luminal and outer aortic wall on axial reformatted images	59:65	25–35
Le, 2016 [11]	3 T, cine bSSFP, luminal diameter of the aortic annulus, sinus and sinotubular junction at diastole measured on the sagittal LVOT plane	91:89	20–69

*n* number of study subjects, *bSSFP* balanced steady-state free precession, *LVOT* left ventricular outflow tract, *VISTA* volume isotropic turbo spin echo acquisition

**Table 38:** Absolute and indexed (to BSA) normal values of aortic sinus luminal diameters and area for men and women at systole and diastole according to [85]

Parameter	Men (n = 60) [mean ± SD (LL–UL) ^a^]		Women (n = 60) [mean ± SD (LL–UL)^a^]	
	Systolic	Diastolic	Systolic	Diastolic
Aortic sinus diameter (cusp-commissure) (mm)	34 ± 3 (27–40)	32 ± 4 (25–39)	30 ± 3 (25–35)	28 ± 3 (23–34)
Aortic sinus diameter (cusp-commissure)/BSA (mm/m^2^)	17 ± 2 (14–20)	16 ± 2 (13–20)	18 ± 2 (14–21)	17 ± 2 (13–20)
Aortic sinus diameter (cusp-cusp) (mm)	36 ± 4 (28–44)	35 ± 4 (27–43)	32 ± 3 (26–38)	31 ± 3 (24–37)
Aortic sinus diameter (cusp-cusp)/BSA (mm/m^2^)	18 ± 2 (14–22)	18 ± 2 (14–22)	19 ± 2 (15–23)	18 ± 2 (14–22)
Aortic sinus area (cm^2^)	9.2 ± 2.1 (5.0–13.4)	8.4 ± 2.0 (4.4–12.4)	7.1 ± 1.4 (4.3–9.9)	6.5 ± 1.3 (3.9–9.1)
Aortic sinus area/BSA (cm^2^/m^2^)	4.6 ± 1.0 (2.6–6.6)	4.2 ± 0.9 (2.4–6.0)	4.2 ± 0.8 (2.6–5.8)	3.8 ± 0.8 (2.2–5.4)

Values obtained as the average of 3 cusp-commissure and 3 cusp-cusp diameters, respectively measured on cross-sectional bSSFP images of the aortic sinus (Fig. 11)

*n* number of study subjects, *SD* standard deviation, *LL* lower limit, *UL* upper limit, *BSA* body surface area

^a^Calculated as mean ± 2*SD

**Table 39:** Normal values of the thoracic aortic luminal diameters for men and women measured at diastole on bSSFP images according to [86]

Level	Men (n = 208) Mean ± SD (LL–UL)^a^	Women (n = 239) Mean ± SD (LL–UL)^a^
Ascending aorta diameter (mm)	27 ± 4 (19–34)	26 ± 4 (18–33)
Proximal descending aorta diameter (mm)	21 ± 3 (15–26)	19 ± 2 (15–23)
Distal descending aorta diameter (mm)	18 ± 3 (13–23)	16 ± 2 (12–20)

Measurements obtained on cross-sectional bSSFP images of the aorta

* bSSFP* balanced steady-state free precession, *n* number of study subjects, *SD* standard deviation, *LL* lower limit, *UL* upper limit

^a^Calculated as mean ± 2*SD

**Table 40:** Absolute and BSA indexed normal values of ascending aortic luminal diameter for men and women of different age categories measured on phase contrast images according to [83]

Age (years)	Men (n = 770) Median (5th–95th percentile)	Women (n = 842) Median (5th–95th percentile)
Absolute values (mm)		
45–54	32 (27–37)	29 (25–34)
55–64	33 (28–41)	30 (26–36)
65–74	34 (29–41)	31 (26–36)
75–84	35 (29–41)	31 (27–37)
Values indexed to BSA (mm/m^2^)		
45–54	16 (13–20)	17 (14–21)
55–64	17 (14–21)	18 (15–22)
65–74	18 (14–22)	18 (15–22)
75–84	19 (15–23)	20 (15–28)

*n* number of study subjects, *BSA* body surface area

**Table 41:** Normal values of descending thoracic aortic diameter, area and wall thickness for young men and women (25–35 years) according to [84]

Parameter	Men (n = 59) Median (10th–90th percentile)	Women (n = 65) Median (10th–90th percentile)
Luminal diameter descending aorta (mm)	19 (17–21)	17 (16–19)
Total diameter descending aorta (mm)	22 (20–24)	20 (19–22)
Luminal area descending aorta (cm^2^)	2.9 (2.2–3.5)	2.3 (2.0–2.8)
Total area descending aorta (cm^2^)	3.9 (3.1–4.6)	3.3 (2.8–3.9)
Wall area descending aorta (cm^2^)	1.0 (0.8–1.2)	1.0 (0.8–1.1)
Wall thickness descending aorta (mm)	1.5 (1.4–1.8)	1.5 (1.4–1.9)

Measurements obtained on axial reformatted images of a fat suppressed 3-dimensional-T1-black blood VISTA (volume isotropic turbo spin echo acquisition) sequence. Calculated average vessel diameter and area as well as wall thickness and wall area of the descending aorta.

*n* number of study subjects

Weighted means were calculated based on the values of the diameter of the aortic root obtained on the 3D bSSFP sequence in LVOT view published by Burman, Davis and Le (Table 42) [11, 85, 86].

**Table 42:** Normal diameters of the aortic root for men and women measured on sagittal left ventricular outflow tract bSSFP

Parameter	Men				Women			
	n	Mean_p_	SD_p_	LL–UL^e^	n	Mean_p_	SD_p_	LL–UL^e^
Aortic annulus diameter (mm)^a^	299	23	5	14–33	328	20	3	14–27
Aortic annulus diameter/BSA (mm/m^2^)^b^	91	12	1	10–14	89	12	1	10–14
Aortic sinus diameter (mm)^c^	359	32	6	19–45	388	28	5	17–38
Aortic sinus diameter/BSA (ml/m^2^)^d^	151	17	2	13–21	149	17	2	13–21
Sinotubular junction diameter (mm)^a^	299	25	6	12–38	328	21	5	12–31
Sinotubular junction diameter/BSA (mm/m^2^)^b^	91	13	2	10–17	89	14	2	10–17

Measurements obtained as shown in Fig. 10.

*n* number of study subjects included in the weighted mean values, *bSSFP* balanced steady-state free precession, *mean*_*p*_ pooled weighted mean, *SD*_*p*_ pooled standard deviation, *LL* lower limit, *UL* upper limit, *BSA* body surface area

^a^Pooled weighted values from references [11, 86]

^b^Values from reference [11]

^c^Pooled weighted values from references [11, 85, 86]

^d^Pooled weighted values from references [11, 85]

^e^Calculated as mean_p_ ± 2*SD_p_

## Normal aortic dimensions in children

### CMR acquisition parameters

There is currently no consensus regarding the optimal CMR sequence to measure aortic diameters and areas in children. In three major publications documenting aortic dimensions in children (Table 43), measurements were obtained with three-dimensional contrast enhanced CMRA [91], gradient echo images [92] and phase contrast cine images [93].

**Table 43:** References, normal aortic dimensions in children

First author, year	CMR technique	n, male:female	Age range (years)
Kaiser, 2008 [91]	1.5 T; contrast enhanced CMRA; shortest diameter measured on cross-sectional reformatted images at 9 locations	30:23	2–20
Kutty, 2012 [93]	1.5 T; magnitude image of a through-plane free-breathing phase contrast sequence; cross-sectional area calculated based on measurement of the maximal external aortic diameter perpendicular to the vessel and perpendicular to the maximal diameter in systole 1 to 2 cm distal to the sinotubular junction	55:50	4–20
Voges, 2012 [92]	3 T; cross sectional cine gradient echo images acquired at 4 positions perpendicular to the aortic axis, measurements obtained at maximal distension of the aorta	30:41	2–28

*n* number of study subject

### CMR analysis methods

To minimize errors in measurement of aorta size, multiplanar reformation should be used to make double-oblique measurements perpendicular to the centerline of the course of the vessel. Kaiser et al. demonstrated that aortic diameter measurements vary slightly based on plane orientation, with a mean difference between measurements on cross-sectional and longitudinal images of 0.16 mm and a coefficient of variability of 2.1% [91].

Aorta measurements should also be made in a consistent manner with respect to the wall of the aorta—outer wall to outer wall, leading edge to leading edge, or luminal diameter. Kutty et al. indicated that in their study measurements were made from outer wall to outer wall [93]. Kaiser et al. and Voges et al. did not provide details on how measurements were made in this regard [91, 92].

### Demographic parameters

Aortic diameters vary by BSA [91, 93] but do not show sex differences in children [92, 93]. Aortic area has not been shown to be dependent upon sex differences either [92].

### Studies included in this review

Reference ranges for parameters measured in children are frequently presented in z-scores and reference curves using the LMS method as described under the LV/RV parameter section in children above.

There are three publications of systematic evaluation of aortic dimensions (diameter and/or area) in children that vary by CMR-technique, measurement technique and data presentation (Table 43).

In this review we present (a) LMS parameters to calculate z-scores for aortic cross-section area based on reference [92] (Tables 44, 45) (b) regression equations of normal aortic diameters measured at 9 different sites based on [91] (Table 46) and (c) normal areas of the ascending aorta from [93] (Table 47).

**Table 44:** LMS parameters to calculate z-scores for aortic cross-sectional area relative to age for boys according to reference [92]

Age^a^	Ascending aorta			Aortic arch			Aortic isthmus			Descending aorta^b^		
	L	M	S	L	M	S	L	M	S	L	M	S
< 1	0.3091	91.5360	0.1207	0.8668	80.1737	0.1898	0.1267	53.0050	0.1987	1.5823	44.6080	0.1100
1	0.3091	120.6960	0.1274	0.8668	101.7001	0.1897	0.1267	68.7198	0.1974	1.5823	57.0317	0.1115
2	0.3091	149.8560	0.1341	0.8668	123.2265	0.1895	0.1267	84.4347	0.1960	1.5823	69.4554	0.1129
3	0.3091	179.0160	0.1408	0.8668	144.7529	0.1894	0.1267	100.1495	0.1946	1.5823	81.8791	0.1143
4	0.3091	208.1812	0.1475	0.8668	166.2791	0.1893	0.1267	115.8653	0.1932	1.5823	94.3035	0.1158
5	0.3091	238.3791	0.1542	0.8668	187.7555	0.1891	0.1267	131.7743	0.1918	1.5823	106.8833	0.1172
6	0.3091	272.8715	0.1604	0.8668	208.8732	0.1890	0.1267	148.2790	0.1904	1.5823	119.9057	0.1186
7	0.3091	311.2493	0.1660	0.8668	229.2411	0.1888	0.1267	164.9648	0.1891	1.5823	133.0488	0.1201
8	0.3091	346.8686	0.1707	0.8668	248.8676	0.1887	0.1267	180.7624	0.1877	1.5823	145.5984	0.1215
9	0.3091	380.0230	0.1748	0.8668	268.0557	0.1886	0.1267	195.7825	0.1863	1.5823	157.5124	0.1229
10	0.3091	413.8181	0.1782	0.8668	287.2956	0.1884	0.1267	210.6578	0.1849	1.5823	169.3366	0.1244
11	0.3091	446.7220	0.1812	0.8668	306.7317	0.1883	0.1267	225.5414	0.1835	1.5823	181.3951	0.1258
12	0.3091	476.5703	0.1841	0.8668	326.2205	0.1881	0.1267	240.3324	0.1822	1.5823	193.8192	0.1272
13	0.3091	501.7973	0.1870	0.8668	345.4511	0.1880	0.1267	254.6975	0.1808	1.5823	206.4812	0.1287
14	0.3091	524.0769	0.1902	0.8668	364.2701	0.1879	0.1267	268.8289	0.1794	1.5823	219.2939	0.1301
15	0.3091	546.3695	0.1937	0.8668	382.7610	0.1877	0.1267	282.9653	0.1780	1.5823	232.0152	0.1316
16	0.3091	569.8955	0.1972	0.8668	400.9805	0.1876	0.1267	296.9424	0.1766	1.5823	244.3629	0.1330
17	0.3091	594.7536	0.2003	0.8668	418.9724	0.1875	0.1267	310.5833	0.1752	1.5823	256.2294	0.1344
18	0.3091	620.9611	0.2025	0.8668	436.7805	0.1873	0.1267	323.7094	0.1739	1.5823	267.5155	0.1359
19	0.3091	647.1204	0.2034	0.8668	454.4484	0.1872	0.1267	336.0814	0.1725	1.5823	278.0681	0.1373
20	0.3091	670.2706	0.2030	0.8668	472.0177	0.1871	0.1267	347.4348	0.1711	1.5823	287.6962	0.1387
21	0.3091	690.0681	0.2014	0.8668	489.5219	0.1869	0.1267	357.7775	0.1697	1.5823	296.3958	0.1402
22	0.3091	706.8583	0.1990	0.8668	506.9924	0.1868	0.1267	367.1860	0.1683	1.5823	304.2102	0.1416
23	0.3091	720.9831	0.1960	0.8668	524.4603	0.1866	0.1267	375.7366	0.1670	1.5823	311.1823	0.1430
24	0.3091	732.2902	0.1926	0.8668	541.9124	0.1865	0.1267	383.4824	0.1656	1.5823	317.3075	0.1445
25	0.3091	740.4053	0.1889	0.8668	559.3076	0.1864	0.1267	390.6086	0.1642	1.5823	322.5658	0.1459
26	0.3091	747.1815	0.1849	0.8668	576.7470	0.1862	0.1267	397.7409	0.1628	1.5823	327.1568	0.1473
27	0.3091	754.8518	0.1805	0.8668	594.3196	0.1861	0.1267	405.3735	0.1614	1.5823	331.4000	0.1488
28	0.3091	763.4054	0.1758	0.8668	611.9863	0.1860	0.1267	413.3799	0.1601	1.5823	335.4719	0.1502
29	0.3091	772.1960	0.1711	0.8668	629.6783	0.1858	0.1267	421.4867	0.1587	1.5823	339.4979	0.1516
30	0.3091	780.9891	0.1663	0.8668	647.3706	0.1857	0.1267	429.5945	0.1573	1.5823	343.5234	0.1531

Aortic area measured at maximum distension of the aorta on cross sectional cine gradient echo images acquired perpendicular to the aortic axis (n = 30)

LMS, L = Lambda (skewness of the distribution), M = Mu (median), S = Sigma (variance)

z-score = [(X/M)^L^ – 1] / (L*S), where X is the measured aortic area in mm^2^ and L, M and S are the values interpolated for the child’s age; lower and upper limits correspond to a z-score of -2 and 2

^a^Age in years

^b^Measured above the diaphragm

**Table 45:** LMS parameters to calculate z-scores for aortic cross-sectional area relative to age for girls according to reference [92]

Age^a^	Ascending aorta			Aortic arch			Aortic isthmus			Descending aorta^b^		
	L	M	S	L	M	S	L	M	S	L	M	S
< 1	− 0.7876	121.1903	0.2152	2.1750	73.6299	0.2114	0.1033	60.0696	0.1621	0.9371	41.0795	0.1398
1	− 0.7876	145.9923	0.2140	2.1750	92.7307	0.2089	0.1033	72.6142	0.1617	0.9371	52.4930	0.1398
2	− 0.7876	170.7944	0.2127	2.1750	111.8315	0.2064	0.1033	85.1587	0.1613	0.9371	63.9065	0.1398
3	− 0.7876	195.5999	0.2114	2.1750	130.9296	0.2039	0.1033	97.7032	0.1609	0.9371	75.3185	0.1398
4	− 0.7876	220.4539	0.2102	2.1750	149.9904	0.2013	0.1033	110.2465	0.1605	0.9371	86.7100	0.1398
5	− 0.7876	245.4281	0.2089	2.1750	168.9588	0.1988	0.1033	122.7870	0.1601	0.9371	98.0510	0.1398
6	− 0.7876	270.5738	0.2076	2.1750	187.8089	0.1963	0.1033	135.3263	0.1597	0.9371	109.3784	0.1398
7	− 0.7876	295.9027	0.2064	2.1750	206.5696	0.1938	0.1033	147.8724	0.1593	0.9371	120.8531	0.1398
8	− 0.7876	321.3290	0.2051	2.1750	225.2367	0.1913	0.1033	160.3915	0.1588	0.9371	132.5201	0.1398
9	− 0.7876	346.5367	0.2038	2.1750	243.7024	0.1887	0.1033	172.7395	0.1584	0.9371	144.0843	0.1398
10	− 0.7876	371.3379	0.2026	2.1750	261.8643	0.1862	0.1033	184.8049	0.1580	0.9371	155.3776	0.1398
11	− 0.7876	395.6874	0.2013	2.1750	279.6207	0.1837	0.1033	196.5286	0.1576	0.9371	166.4608	0.1398
12	− 0.7876	419.5583	0.2000	2.1750	296.8402	0.1812	0.1033	207.8452	0.1572	0.9371	177.3057	0.1398
13	− 0.7876	442.8024	0.1988	2.1750	313.4236	0.1787	0.1033	218.7232	0.1568	0.9371	187.8984	0.1398
14	− 0.7876	465.1326	0.1975	2.1750	329.2852	0.1761	0.1033	229.1136	0.1564	0.9371	198.1163	0.1398
15	− 0.7876	486.2071	0.1962	2.1750	344.3674	0.1736	0.1033	238.9630	0.1560	0.9371	207.7776	0.1398
16	− 0.7876	505.7398	0.1950	2.1750	358.6387	0.1711	0.1033	248.2461	0.1556	0.9371	216.7982	0.1398
17	− 0.7876	523.5836	0.1937	2.1750	372.0983	0.1686	0.1033	256.9723	0.1552	0.9371	225.1710	0.1398
18	− 0.7876	539.7165	0.1924	2.1750	384.7434	0.1661	0.1033	265.1479	0.1547	0.9371	232.7857	0.1398
19	− 0.7876	554.1764	0.1912	2.1750	396.5833	0.1635	0.1033	272.7929	0.1543	0.9371	239.5830	0.1398
20	− 0.7876	567.1207	0.1899	2.1750	407.6567	0.1610	0.1033	279.9469	0.1539	0.9371	245.6109	0.1398
21	− 0.7876	578.7817	0.1886	2.1750	418.0442	0.1585	0.1033	286.6730	0.1535	0.9371	251.0496	0.1398
22	− 0.7876	589.4770	0.1873	2.1750	427.8971	0.1560	0.1033	293.0630	0.1531	0.9371	256.1671	0.1398
23	− 0.7876	599.5300	0.1861	2.1750	437.3887	0.1534	0.1033	299.2101	0.1527	0.9371	261.1406	0.1398
24	− 0.7876	609.3164	0.1848	2.1750	446.7229	0.1509	0.1033	305.2232	0.1523	0.9371	266.1367	0.1398
25	− 0.7876	619.1593	0.1835	2.1750	456.0570	0.1484	0.1033	311.2003	0.1518	0.9371	271.2515	0.1398
26	− 0.7876	629.1747	0.1822	2.1750	465.4360	0.1459	0.1033	317.2057	0.1514	0.9371	276.5640	0.1398
27	− 0.7876	639.3019	0.1810	2.1750	474.8382	0.1433	0.1033	323.2314	0.1510	0.9371	281.9813	0.1398
28	− 0.7876	649.4860	0.1797	2.1750	484.2530	0.1408	0.1033	329.2650	0.1506	0.9371	287.4341	0.1398
29	− 0.7876	659.6776	0.1784	2.1750	493.6694	0.1383	0.1033	335.2995	0.1502	0.9371	292.8916	0.1398
30	− 0.7876	669.8691	0.1772	2.1750	503.0858	0.1358	0.1033	341.3341	0.1498	0.9371	298.3491	0.1398

Aortic area measured at maximum distension of the aorta on cross sectional cine gradient echo images acquired perpendicular to the aortic axis (n = 41)

LMS, L = Lambda (skewness of the distribution), M = Mu (median), S = Sigma (variance)

z-score = [(X/M)^L^ – 1] / (L*S), where X is the measured aortic area in mm^2^ and L, M and S are the values interpolated for the child’s age; lower and upper limits correspond to a z-score of -2 and 2

^a^Age in years

^b^Measured above the diaphragm

**Table 46:** Normal aortic diameters in children measured on a contrast enhanced 3D-CMRA according to reference [91]

Site	Predicted diameter (mm)	SD of residuals (mm)
Aortic sinus	0.57 + 19.37*BSA^0.5^	2.38
Sinotubular junction	− 0.03 + 16.91*BSA^0.5^	1.92
Ascending aorta	− 1.33 + 18.6*BSA^0.5^	1.99
Proximal to the origin of the brachiocephalic artery	− 3.38 + 20.07*BSA^0.5^	1.69
First transverse segment	− 3.52 + 18.66*BSA^0.5^	1.63
Second transverse segment	− 2.63 + 16.5*BSA^0.5^	1.31
Isthmic región	− 3.37 + 16.52*BSA^0.5^	1.46
Descending aorta	− 1.12 + 14.42*BSA^0.5^	1.64
Thoracoabdominal aorta at the level of the diaphragm	1.27 + 9.89*BSA^0.5^	1.34

Shortest diameter measured on cross-sectional reformatted images (n = 53). Sites of measurement are shown in Fig. 9

z-score = (measured diameter − predicted diameter)/SD of residuals; lower and upper limits correspond to a z-score of -2 and 2

*BSA* body surface area, *SD* standard deviation

**Table 47:** Normal aortic area on phase contrast cine images according to reference [93]

Site	Predicted area (cm^2^)
Ascending aorta	− 0.0386 + 2.913*BSA

Cross sectional area calculated based on measurement of the maximal external aortic diameter perpendicular to the vessel and perpendicular to the maximal diameter in systole 1 to 2 cm distal to the sinotubular junction on the magnitude image of a phase contrast cine sequence (n = 105)

*BSA* body surface area

Due to the differences in acquisition sequences, measurement techniques, and presentation of results, weighted mean values are not presented.

## Normal aortic distensibilityand pulse wave velocity (PWV) in adults

### CMR acquisition parameters

Pulse wave velocity (PWV) calculations using a velocity-encoded CMR with phase contrast sequences allow accurate assessment of aortic systolic flow wave and the blood flow velocity. The sequence should be acquired at the level of the bifurcation of the pulmonary trunk, perpendicular to both, the ascending and descending aorta. The distance between two aortic locations (aortic length) can be estimated from axial and coronal cine breath hold bSSFP sequences covering the whole aortic arch [94]. Alternatively, sagittal oblique views of the aortic arch can be acquired e.g. using a black blood spin echo sequence [88].

Another parameter of aortic stiffness is aortic distensibility. The cross sectional aortic area at different phases of the cardiac cycle is measured using ECG-gated bSSFP cine imaging to assess aortic distensibility by CMR. Modulus images of cine phase contrast CMR can be used as well [95].

### CMR analysis methods

**Fig. 12:**
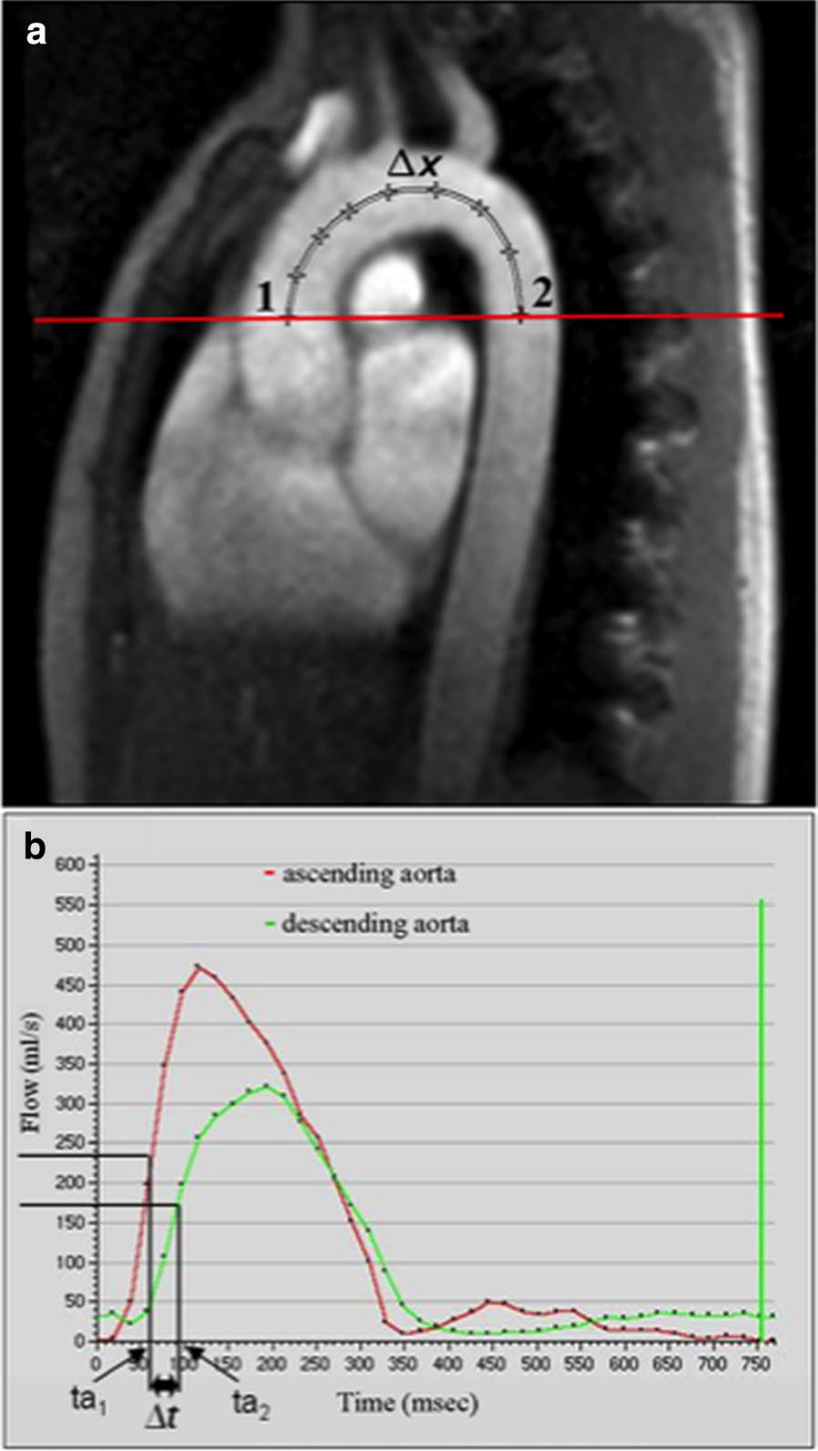
Measurement of pulse wave velocity according to reference [92]. Δx: length of the centerline between the sites of flow measurement in the ascending and descending aorta; Δt: time delay between the flow curves obtained in the descending aorta relative to the flow curve obtained in the ascending aorta calculated between the midpoint of the systolic up slope tails on the flow versus time curves of the ascending aorta (ta1) and the descending aorta (ta2)

PWV is the most validated method to quantify arterial stiffness using CMR. PWV is calculated by measuring the pulse transit time of the flow curves (Δt) and the distance (D) between the ascending and descending aortic locations of the phase contrast acquisition [88]: Aortic PWV = D/ Δt (Fig. 12).

PWV increases with stiffening of arteries since the stiffened artery conducts the pulse wave faster compared to more distensible arteries.

Aortic distensibility is calculated with the fallowing formula after measuring the minimum and maximum aortic cross sectional area [96]:

$$\begin{aligned} & {\text{Aortic distensibility}} = \left( {{\text{minimal area}} - {\text{maximal area}}} \right)/\left( {{\text{minimal area}} \times \Delta {\text{P}} \times {1}000} \right) \\ & {\text{where }}\Delta {\text{P is the pulse pressure in mmHg}}. \\ \end{aligned}$$

### Demographic parameters

Greater ascending aorta diameter and changes in aortic arch geometry with greater age was associated with increased regional stiffness of the aorta, especially of the ascending portion. The relationship of age with measures of aortic stiffness is non-linear and the decrease of aortic distensibility occurs particularly before the fifth decade of life [88]. Males have stiffer aortas compared to females [97].

### Studies included in this review

Two studies with a total sample size of more than 40 subjects reported reference ranges for PWV and/or distensibility in healthy subjects (Table 48): Kim et al. present reference ranges for PWV and distensibility for a cohort of 124 healthy Asian subjects [98]. Since both parameters have been shown to be highly age dependent, reference ranges are given per age decile according to the original publication [98]. However, sample size per decile was small (between 21 and 28 subjects) and standard deviations are relatively large (Tables 49, 50). In the study by Eikendal et al. reference values for PWV in young (25–35 years) healthy subjects are given (Table 51) [84].

**Table 48:** References, normal aortic pulse wave velocity (PWV) and distensibility

First author, year	CMR technique	n, male:female	Age range (years)
Kim, 2013 [98]	1.5 T, phase contrast CMR to calculate PWV for 3 distances of the aorta; transit time calculated from the midpoint of the systolic up-slope on the flow versus time curve; cross sectional cine bSSFP at 4 levels of the aorta to calculate distensibility	61:63	20–79
Eikendal, 2016 [84]	3 T, phase contrast CMR to calculate PWV for 2 distances of the aorta, time delay calculated from velocity–time curves	57:61	25–35

*n* number of study subjects, *bSSFP* balanced steady-state free precession, *PWV* pulse wave velocity

**Table 49:** Normal values of regional aortic distensibility for men and women according to [98]

Level	Age (years)	Men (n = 61) Mean ± SD (10^−3^ mm/Hg)	Women (n = 63) Mean ± SD (10^–3^ mm/Hg)
Ascending aorta^a^	20–29	5.6 ± 1.5	7.9 ± 3.4
	30–39	3.6 ± 1.4	6.5 ± 3.0
	40–49	3.5 ± 1.5	5.3 ± 1.2
	50–59	3.2 ± 1.6	3.6 ± 1.1
	60–69	2.1 ± 1.3	2.7 ± 1.0
Proximal descending aorta^a^	20–29	4.2 ± 0.9	6.0 ± 1.4
	30–39	3.8 ± 1.3	5.5 ± 1.9
	40–49	3.3 ± 0.6	4.2 ± 1.2
	50–59	2.9 ± 1.1	3.7 ± 1.3
	60–69	2.3 ± 0.9	3.1 ± 0.9

*n* number of study subjects

^a^Measurements obtained at the level of the bifurcation of the pulmonary artery

**Table 50:** Normal values for aortic pulse wave velocity according to [98]

Age (years)	n	Median (5th–95th percentile) (m/s)
20–29	26	3.7 (3.4–4.0)
30–39	28	3.8 (3.5–6.0)
40–49	24	4.3 (3.7–5.0)
50–59	25	5.6 (5.4–7.2)
60–69	21	9.0 (7.4–12.4)

Regional pulse wave velocity from the ascending to the upper descending thoracic aorta

*n* number of study subjects

**Table 51:** Normal values for aortic pulse wave velocity in young men and women (25–35 years) according to [84]

Men (n = 57) Median (10th–90th percentile) (m/s)	Women (n = 61) Median (10th–90th percentile) (m/s)
4.6 (3.9–5.6)	4.5 (3.6–6.0)

Regional pulse wave velocity from the ascending to the upper descending thoracic aorta

*n* number of study subjects

With respect to PWV, in this review we present reference ranges for the distance between the ascending and the proximal descending thoracic aorta. This range is frequently measured since measurements at both locations can be obtained simultaneously on a single 2D acquisition at the level of the bifurcation of the pulmonary artery. PWV for other distances (ascending to distal descending aorta and total PWV) can be found in the original publications [84, 98]. In addition to the ascending and proximal descending thoracic aorta, distensibility for the distal descending and the total aorta is presented in the original publication by Kim et al. [98].

## Normal aortic distensibility and pulse wave velocity (PWV) in children

### CMR acquisition parameters

In the only publication of aortic distensibility and PWV by CMR in children, distensibility was obtained on gradient echo cine images and pulse wave velocity was measured on phase-contrast cine CMR [92].

### CMR analysis methods

Distensibility was calculated as (A_max_ – A_min_)/A_min_ x (P_max_ – P_min_), where A_max_ and A_min_ represent the maximal and minimal cross-sectional area of the aorta, and P_max_ and P_min_ represent the systolic and diastolic blood pressure measured with a sphygmomanometer cuff around the right arm.

PWV was calculated as Δx/Δt, where Δx is defined as the length of the centerline between the sites of flow measurement in the ascending and descending aorta and Δt represents the time delay between the flow curve obtained in the descending aorta relative to the flow curve obtained in the ascending aorta (Fig. 12).

### Demographic parameters

Aortic distensibility and PWV did not vary by gender. Aortic distensibility decreases with age and correlates with height, body weight and BSA. PWV has been shown to increase with age [92].

### Studies included in this review

There is a single publication only of a systematic evaluation of normal aortic distensibility and PWV in children (Table 52). In this review we present LMS parameters to calculate z-scores for distensibility of the ascending aorta and PWV based on reference [92] (Tables 53, 54). In the original publication LMS parameters for distensibility at 3 other levels of the thoracic aorta (aortic arch, aortic isthmus and distal descending aorta) are presented in addition [92].

**Table 52:** References, normal distensibility and pulse wave velocity (PWV) in children

First author, year	CMR technique	n, male:female	Age range (years)
Voges, 2012 [92]	3 T; cross sectional cine GRE at 4 levels of the thoracic aorta to calculate distensibility; phase contrast CMR to calculate PWV for the distance between the sinotubular junction and the proximal descending aorta, transit time calculated from the midpoint of the systolic up-slope on the flow versus time curve	30:41	2–28

*n* number of study subjects, *GRE* gradient echo, *PWV* pulse wave velocity

**Table 53:** LMS parameters to calculate z-scores for distensibility of the ascending aorta relative to age in children according to reference [92]

Age (years)	Male (n = 30)			Female (n = 41)		
	L	M	S	L	M	S
< 1	− 0.1879	12.3602	0.3680	− 0.0721	12.7303	0.2388
1	− 0.1879	11.9220	0.3680	− 0.0721	12.5028	0.2396
2	− 0.1879	11.4838	0.3680	− 0.0721	12.2753	0.2403
3	− 0.1879	11.0456	0.3680	− 0.0721	12.0477	0.2411
4	− 0.1879	10.6075	0.3680	− 0.0721	11.8176	0.2419
5	− 0.1879	10.1700	0.3680	− 0.0721	11.5817	0.2427
6	− 0.1879	9.7343	0.3680	− 0.0721	11.3421	0.2435
7	− 0.1879	9.2990	0.3680	− 0.0721	11.1121	0.2443
8	− 0.1879	8.8602	0.3680	− 0.0721	10.9051	0.2451
9	− 0.1879	8.4151	0.3680	− 0.0721	10.7290	0.2459
10	− 0.1879	7.9776	0.3680	− 0.0721	10.5679	0.2467
11	− 0.1879	7.5683	0.3680	− 0.0721	10.3851	0.2474
12	− 0.1879	7.2051	0.3680	− 0.0721	10.1582	0.2482
13	− 0.1879	6.9030	0.3680	− 0.0721	9.8884	0.2490
14	− 0.1879	6.6697	0.3680	− 0.0721	9.5911	0.2498
15	− 0.1879	6.5089	0.3680	− 0.0721	9.2905	0.2506
16	− 0.1879	6.4138	0.3680	− 0.0721	9.0033	0.2514
17	− 0.1879	6.3729	0.3680	− 0.0721	8.7345	0.2522
18	− 0.1879	6.3745	0.3680	− 0.0721	8.4850	0.2529
19	− 0.1879	6.4062	0.3680	− 0.0721	8.2574	0.2537
20	− 0.1879	6.4551	0.3680	− 0.0721	8.0546	0.2545
21	− 0.1879	6.5111	0.3680	− 0.0721	7.8749	0.2553
22	− 0.1879	6.5646	0.3680	− 0.0721	7.7106	0.2561
23	− 0.1879	6.6062	0.3680	− 0.0721	7.5479	0.2569
24	− 0.1879	6.6277	0.3680	− 0.0721	7.3842	0.2577
25	− 0.1879	6.6242	0.3680	− 0.0721	7.2113	0.2584
26	− 0.1879	6.5975	0.3680	− 0.0721	7.0343	0.2592
27	− 0.1879	6.5577	0.3680	− 0.0721	6.8647	0.2600
28	− 0.1879	6.5116	0.3680	− 0.0721	6.6951	0.2608
29	− 0.1879	6.4643	0.3680	− 0.0721	6.5250	0.2616
30	− 0.1879	6.4170	0.3680	− 0.0721	6.3550	0.2624

Distensibility was calculated based on measurements of the aortic area at systole and diastole on cross sectional cine gradient echo images obtained perpendicular to the axis of the ascending thoracic aorta.

LMS, L = Lambda (skewness of the distribution), M = Mu (median), S = Sigma (variance).

z-score = [(X/M)^L^ – 1] / (L*S), where X is the measured aortic distensibility in 10^–3^ mm Hg^−1^ and L, M and S are the values interpolated for the child’s age; lower and upper limits correspond to a z-score of -2 and 2.

*n* number of study subjects.

**Table 54:** LMS parameters to calculate z-scores for pulse wave velocity (PWV) relative to age in children according to reference [92]

Age (years)	Male (n = 30)			Female (n = 41)		
	L	M	S	L	M	S
< 1	1.4844	3.4147	0.2122	− 1.5196	2.7808	0.1468
1	1.4844	3.4367	0.2122	− 1.5196	2.8144	0.1469
2	1.4844	3.4587	0.2122	− 1.5196	2.8481	0.1469
3	1.4844	3.4808	0.2122	− 1.5196	2.8817	0.1469
4	1.4844	3.5028	0.2122	− 1.5196	2.9154	0.1470
5	1.4844	3.5248	0.2122	− 1.5196	2.9490	0.1470
6	1.4844	3.5469	0.2122	− 1.5196	2.9827	0.1470
7	1.4844	3.5689	0.2122	− 1.5196	3.0163	0.1470
8	1.4844	3.5909	0.2122	− 1.5196	3.0499	0.1471
9	1.4844	3.6129	0.2122	− 1.5196	3.0836	0.1471
10	1.4844	3.6350	0.2122	− 1.5196	3.1172	0.1471
11	1.4844	3.6570	0.2122	− 1.5196	3.1509	0.1471
12	1.4844	3.6790	0.2122	− 1.5196	3.1845	0.1472
13	1.4844	3.7011	0.2122	− 1.5196	3.2182	0.1472
14	1.4844	3.7231	0.2122	− 1.5196	3.2518	0.1472
15	1.4844	3.7451	0.2122	− 1.5196	3.2855	0.1473
16	1.4844	3.7672	0.2122	− 1.5196	3.3192	0.1473
17	1.4844	3.7892	0.2122	− 1.5196	3.3528	0.1473
18	1.4844	3.8112	0.2122	− 1.5196	3.3865	0.1473
19	1.4844	3.8333	0.2122	− 1.5196	3.4201	0.1474
20	1.4844	3.8553	0.2122	− 1.5196	3.4538	0.1474
21	1.4844	3.8773	0.2122	− 1.5196	3.4875	0.1474
22	1.4844	3.8994	0.2122	− 1.5196	3.5211	0.1475
23	1.4844	3.9214	0.2122	− 1.5196	3.5548	0.1475
24	1.4844	3.9434	0.2122	− 1.5196	3.5885	0.1475
25	1.4844	3.9655	0.2122	− 1.5196	3.6221	0.1476
26	1.4844	3.9875	0.2122	− 1.5196	3.6558	0.1476
27	1.4844	4.0096	0.2122	− 1.5196	3.6895	0.1476
28	1.4844	4.0316	0.2122	− 1.5196	3.7231	0.1476
29	1.4844	4.0536	0.2122	− 1.5196	3.7568	0.1477
30	1.4844	4.0757	0.2122	− 1.5196	3.7905	0.1477

Pulse wave velocity calculated by phase contrast CMR for the distance between the sinotubular junction and the proximal descending aorta. Transit time calculated from the midpoint of the systolic up-slope on the flow versus time curve (Fig. 12).

LMS, L = Lambda (skewness of the distribution), M = Mu (median), S = Sigma (variance)

z-score = [(X/M)^L^ – 1] / (L*S), where X is the measured pulse wave velocity in m/s and L, M and S are the values interpolated for the child’s age; lower and upper limits correspond to a z-score of -2 and 2.

*n* number of study subjects

## Normal dimensions and distension of the pulmonary arteries in adults

### CMR acquisition parameters

**Fig. 13:**
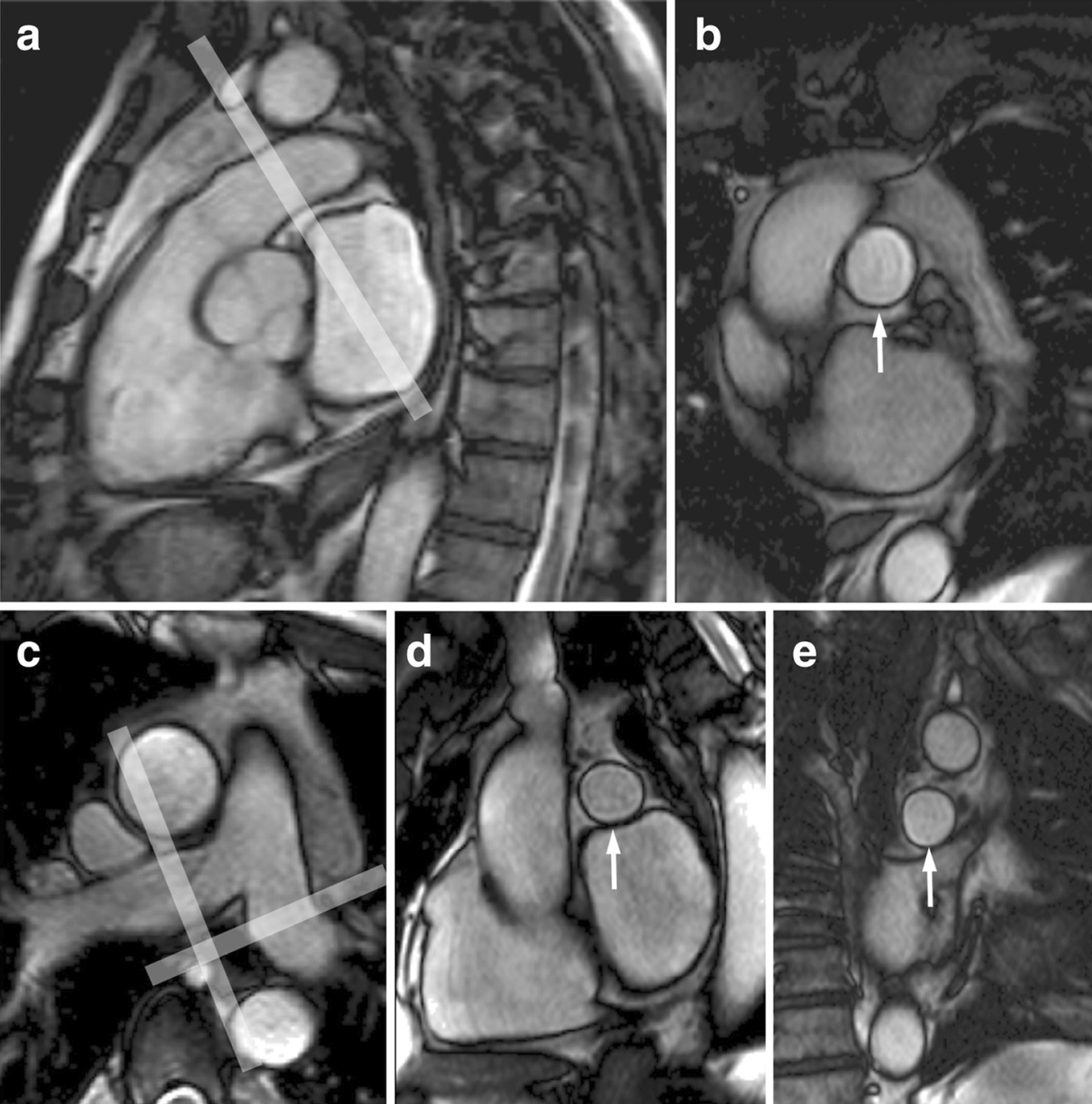
Measurement of the dimensions of the pulmonary arteries on bSSFP images according to [99]. Oblique sagittal image of the main pulmonary artery (**a**). The pale band in **a** shows the acquisition plane of the cross sectional image of the main pulmonary artery in **b**. Right and left pulmonary arteries on the scout image (**c**) with band indicating the location of cine acquisitions transecting the right (**d**) and left (**e**) pulmonary artery

In the study by Burman et al. listed in this review [99] dimensions of the pulmonary arteries were measured on bSSFP images (Table 55). Burman et al. acquired cross sectional images of the main and the right and left pulmonary artery based on an oblique sagittal image of the RV outflow tract and pulmonary trunk, respectively (for the main pulmonary artery) and an axial image acquired at the level of the bifurcation of the main pulmonary artery (for the left and right pulmonary artery) (Fig. 13). With three-dimensional acquisition, reconstruction of the imaging plane can be performed after image acquisition using multiplanar reformation.

**Table 55:** Reference, normal dimensions and distension of the pulmonary arteries in adults

First author, year	CMR technique	n, male:female	Age range (years)
Burman, 2016 [99]	1.5 T, cross sectional bSSFP, luminal area and mean diameters	60:60	20–79

*n* number of study subjects, *bSSFP* balanced steady-state free precession

Other sequences could also be used to obtain dimensions of the pulmonary arteries such as a three-dimensional contrast enhanced CMRA with contrast timing optimized to enhance the pulmonary arteries. Non-contrast techniques include respiratory and ECG-gated 3D bSSFP sequence and cine phase contrast imaging. However, similar to the aorta, measurements of the pulmonary artery are expected to vary by the sequence type and might not be comparable [82]. In contrast to static sequences, acquisition of dynamic sequences, e.g. cine bSSFP, enable measurements at systole and diastole and calculation of distension.

### CMR analysis methods

Luminal areas and diameters of the pulmonary arteries were measured on cross sectional images of the respective vessel at minimal diastolic and minimal systolic expansion. Since the cross section of the vessel is usually not perfectly circular, data presented in Table 56 shows the mean diameter of two diameters that were acquired per vessel and phase calculated from the greatest diameter and the lesser diameter orthogonal to the greater diameter. Percent systolic distension was calculated as [(maximum area – minimum area) * 100/minimum area].

**Table 56:** Normal dimensions and distension of the pulmonary arteries in adults according to [99]

Vessel	Parameter	Men (n = 60)			Women (n = 60)		
		Mean	SD	LL–UL^a^	Mean	SD	LL–UL^a^
MPA	Systolic diameter (mm)	27.4	2.6	21–33	25.3	2.6	19–31
	Diastolic diameter (mm)	22.9	2.4	19–27	21.2	2.1	17–25
	Systolic area (cm^2^)	5.9	1.1	3.7–8.1	5.0	1.0	3.0–7.0
	Diastolic area (cm^2^)	4.2	0.8	2.6–5.8	3.6	0.7	2.2–5.0
	Distension (%)	42.7	17.2	9–77	41.8	15.7	10–74
RPA	Systolic diameter (mm)	20.2	2.9	14–26	17.8	2.4	14–22
	Diastolic diameter (mm)	16.6	2.8	11–23	14.7	2.2	11–19
	Systolic area (cm^2^)	3.3	1.0	1.3–5.3	2.6	0.7	1.2–4.0
	Diastolic area (cm^2^)	2.2	0.8	0.6–3.8	1.8	0.6	0.6–3.0
	Distension (%)	50.6	16.9	17–85	48.2	14.5	18–78
LPA	Systolic diameter (mm)	20.1	2.4	16–24	18.4	2.1	14–22
	Diastolic diameter (mm)	17.3	2.5	11–23	15.9	2.0	12–20
	Systolic area (cm^2^)	3.3	0.8	1.7–4.9	2.8	0.6	1.6–4.0
	Diastolic area (cm^2^)	2.4	0.7	1.0–3.8	2.1	0.5	1.1–3.1
	Distension (%)	35.6	10.1	16–56	35.2	10.3	15–55

*n* number of study subjects, *SD* standard deviation, *LL* lower limit, *UL* upper limit, *MPA* main pulmonary artery, *RPA* right pulmonary artery, *LPA* left pulmonary artery

^a^Calculated as mean ± 2*SD

### Demographic parameters

Area and mean diameters of the pulmonary arteries are greater in men compared to women and greater in systole compared to diastole. Some measurements of the area and the mean diameter of the pulmonary arteries slightly increase with BSA and age, while systolic distension decrease with age. For a detailed description of the relationship of the area, mean diameters and systolic distension of the pulmonary arteries with age and BSA please see [99].

### Studies included in this review

One publication of reference ranges of the area, diameters and distension of the pulmonary arteries in adults was found using a current CMR technique, sufficient sample size (> 40 subjects per gender) and a clear description of image acquisition and measurements [99]. In the original publication, reference ranges were presented for age deciles for subjects between 20 and 79 years with 10 subjects per decile and gender. However, since the differences between age deciles were small and might not be clinically relevant and for sample size considerations, in the current review only values of the entire cohort separated by gender are presented.

## Normal dimensions of the pulmonary arteries in children

### CMR acquisition parameters

In analogy to dimensions of the pulmonary arteries in adults, different sequences might be used to obtain measurements. In the studies listed below, a contrast enhanced three-dimensional CMRA and a cross sectional through-plane free-breathing phase contrast sequence were acquired to obtain the measurements [93, 100].

### CMR analysis methods

**Fig. 14:**
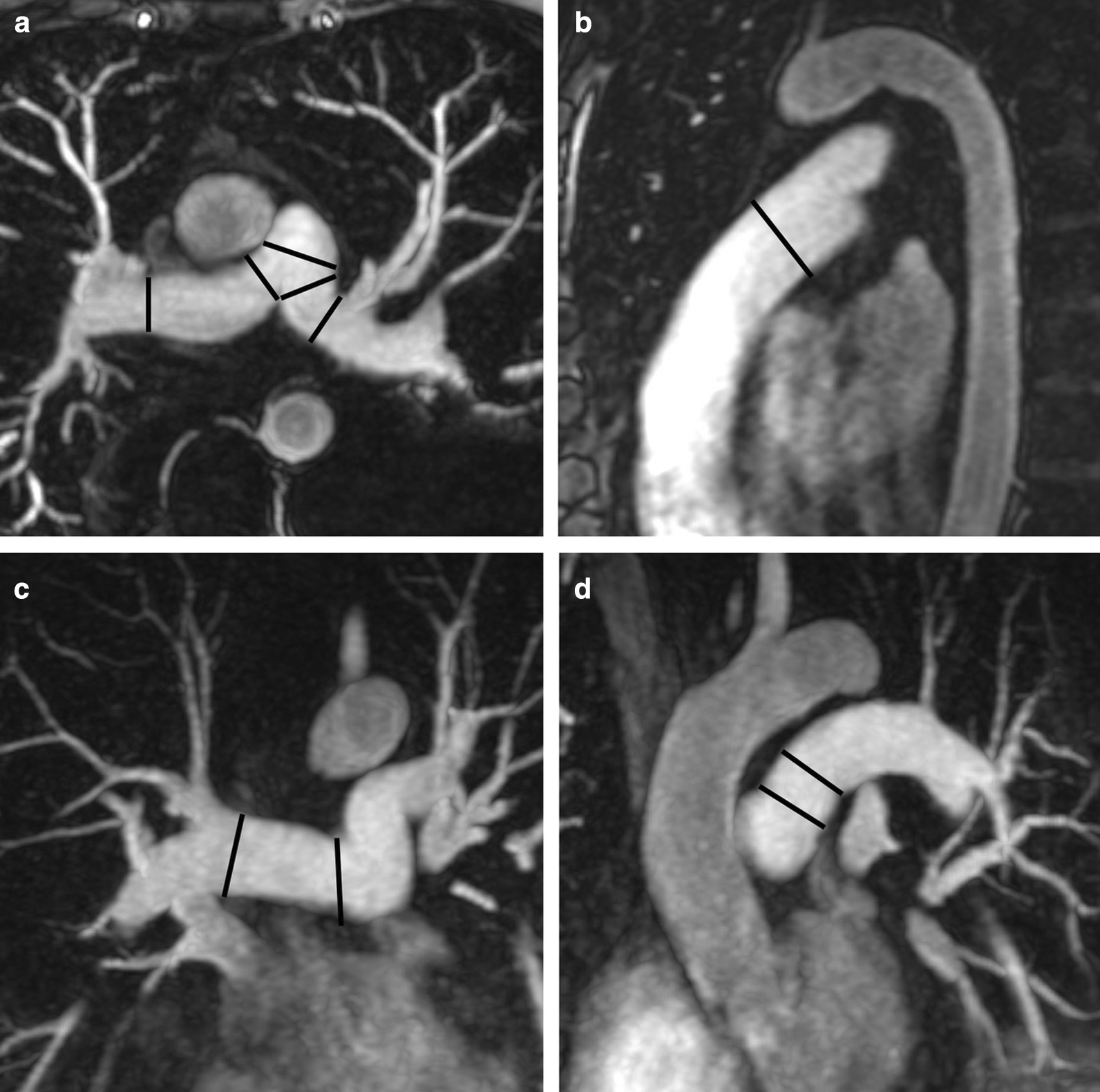
Measurement of the diameters of the pulmonary arteries according to reference [100]. Diameters were measured perpendicular to the vessel on maximum intensity projection images. The diameters of the main pulmonary artery were obtained on an axial (**a**) and sagittal oblique (**b**) view and the diameters of the proximal and distal right and left pulmonary artery were obtained on axial (**a**) and right and left anterior oblique (paracoronal) views (**c**, **d**), respectively

Knobel et al. obtained measurements of the pulmonary arteries on reconstructed maximum intensity projection (MIP) images (slice thickness is not mentioned) perpendicular to the respective vessel (Fig. 14) [100]. The diameter of the main pulmonary artery was obtained on an axial and a reformatted sagittal oblique view, the diameters of the proximal and distal right and left pulmonary artery were measured on an axial and reformatted right and left anterior oblique (coronal oblique) views.

In the study by Kutty et al. the maximal external diameter of the main pulmonary artery (*d*_1_) was measured on the cross sectional magnitude image of the PC sequence in systole and also the diameter (*d*_2_) perpendicular to *d*_1_ [93]. After derivation of the radii (*r*_1_ and *r*_2_), the area was calculated as π*r*_1_*r*_2_.

### Demographic parameters

In both studies a relationship between pulmonary artery diameter and BSA was described [93, 100]. Kutty et al. could not find a significant gender difference of the size of the main pulmonary artery.

### Studies included in this review

Two studies were identified presenting normal values of the size of the pulmonary arteries in children [93, 100] (Table 57). Knobel et al. included 69 children ranging from 2 to 20 years with a previous history of malignancy that were assessed for potential port-a-cath related complications but normal cardiovascular anatomy and no evidence of cardiovascular disease [100] (Table 58). In the study by Kutty et al. 105 normal healthy subjects between 4 and 20 years were included (data presented here; Table 59) and also subjects with repaired tetralogy of Fallot (not presented in this review) [93].

**Table 57:** References, normal dimensions of the pulmonary arteries in children

First author, year	CMR technique	n, male:female	Age range (years)
Knobel, 2011 [100]	1.5 T, contrast enhanced CMRA, diameters measured on images perpendicular to the vessel	41:28	2–20
Kutty, 2012 [93]	1.5 T; magnitude image of a through-plane free-breathing phase contrast sequence; cross sectional area calculated based on measurements of the maximal external aortic diameter perpendicular to the vessel and perpendicular to the maximal diameter obtained midway between the level of the pulmonary valve and the bifurcation of the branch pulmonary arteries	55:50	4–20

*n* number of study subjects

**Table 58:** Normal diameters of the pulmonary arteries in children measured on a contrast enhanced CMRA according to reference [100]

Site	Predicted diameter (mm)	SD of residuals (mm)
Main pulmonary artery (axial)	4.85 + 13.43*BSA^0.5^	2.72
Main pulmonary artery (sagittal)	1.04 + 17.07* BSA^0.5^	2.01
Proximal right pulmonary artery (axial)	2.63 + 9.19* BSA^0.5^	1.65
Distal right pulmonary artery (axial)	3.9 + 6.25* BSA^0.5^	1.49
Proximal right pulmonary artery (RAO)	− 0.69 + 14.3* BSA^0.5^	1.76
Distal right pulmonary artery (RAO)	− 1.08 + 14.62* BSA^0.5^	1.6
Proximal left pulmonary artery (axial)	1.7 + 11.27* BSA^0.5^	1.37
Distal left pulmonary artery (axial)	− 0.1 + 11.89* BSA^0.5^	1.51
Proximal left pulmonary artery (LAO)	− 2.13 + 16.82* BSA^0.5^	1.88
Distal left pulmonary artery (LAO)	− 2.08 + 13.64* BSA^0.5^	1.5

Diameters measured perpendicular to the vessel (Fig. 14).

Fitting model for regression: diameter = a + b*BSA^0.5^

z-score = (measured diameter – predicted diameter)/SD of residuals; lower and upper limits correspond to a z-score of -2 and 2.

*BSA* body surface area, *SD* standard deviation, *RAO* right anterior oblique view (paracoronal, parallel to right pulmonary artery; Fig. 14), *LAO* left anterior oblique view (paracoronal, parallel to left pulmonary artery; Fig. 14).

**Table 59:** Normal pulmonary artery area measured on phase contrast cine images according to reference [93]

Site	Predicted area (cm^2^)
Ascending aorta	− 0.2880 + 3.386*BSA

Cross sectional area calculated based on measurements of the maximal external aortic diameter perpendicular to the vessel and perpendicular to the maximal diameter obtained midway between the level of the pulmonary valve and the bifurcation of the branch pulmonary arteries.

*BSA* body surface area

Due to the differences in sequence type, measurement technique and data presentation the normal values of the two studies are presented separately.

## Normal values of myocardial T1 relaxation time and the extracellular volume (ECV)

### CMR acquisition parameters

The field of myocardial T1 mapping has matured significantly with several studies reporting T1 relaxation times for normal cohorts [101]. An Expert Consensus document on parametric mapping has been published providing recommendations for the practical clinical application of T1, T2, T2*, and ECV mapping [102]. Most of the published myocardial T1 values have been acquired using variants of the Modified Look-Locker Inversion Recovery (MOLLI) technique [103] including the shortened-MOLLI (ShMOLLI) [104] method. Saturation recovery based techniques such as saturation recovery single-shot acquisition (SASHA) are alternative techniques but have less clinical evidence to date [105]. Images are typically acquired in diastole to limit cardiac motion and respiratory motion correction.

Native T1 maps are acquired without a contrast agent. Post contrast T1 maps allow assessment of gadolinium contrast distribution, as these agents shorten the T1 relaxation time of water. T1 maps acquired 10–30 min following injection of an extra-cellular non-protein binding gadolinium contrast agent can be used to quantify the extracellular volume fraction (ECV) [102]. Post contrast T1 values have been performed following a bolus or primed infusion (Equilibrium-EQCMR) with good agreement of ECV values up to 40% [106]. While the hematocrit can be approximated from the T1 of the blood in the LV cavity (“synthetic T1”), assessment of hematocrit by blood sampling as close as possible in time to the CMR (less than 24 h) is preferred due to normal daily variation of hematocrit [102].

### Factors affecting T1 relaxation time and ECV

There are a number of CMR acquisition factors that can affect the measurement of normal T1 and ECV values. Field strength has a significant effect on T1 values; with 3 T scans producing 28% higher native T1 and 14% higher post contrast T1 values when compared with 1.5 T [107]. Post contrast T1 is also affected by the dose and relaxivity of the contrast agent used, contrast clearance, and the time between injection and measurement [107–109]. There is also greater heterogeneity for a T1 native normal range at 3 T [107, 110, 111]. Further, it has been shown that T1 varies by cardiac phase (diastole versus systole) and region of measurement (septal versus non-septal) [107]. ECV values are relatively unaffected by field strength (3 T versus 1.5 T). Both native T1 and ECV values have been shown to be less reliable in the infero-lateral wall likely secondary to off-resonance effects [107, 112].

A number of pulse sequence parameters can affect normal values. For MOLLI pulse sequences the number of inversions, number of images following each inversion, and number of recovery beats between inversion pulses, and the flip angle affect normal values [101]. Furthermore, the type of inversion pulses, which may be vendor specific can also affect T1 values.

The aforementioned factors contribute to the large heterogeneity of published reference ranges. Heterogeneity in published values are present even if the same manufacturer scanner was used at the same field strength with the same pulse sequence [101]. It is thus imperative to standardize local pulse sequences and sequence parameters, and to follow current consensus guidelines for establishing site specific reference ranges [102]. In contrast to other CMR parameters, the SCMR has indicated that literature normal values of T1 relaxation times should not serve as absolute reference values, but rather than site-specific reference ranges should be established [102].

### CMR analysis methods

T1 maps are based on pixel-wise quantification of longitudinal relaxation from the T1-weighted source images. The native T1 relaxation time, expressed in milliseconds (ms), is a composite measurement reflecting the signal from water within multiple compartments within the myocardium including myocytes, the blood pool, and the interstitial space [113]. Under assumptions of an equilibrium of gadolinium concentrations between the blood pool and interstitium, pre and post contrast blood and myocardial T1 values can be used to quantify the partition coefficient of gadolinium which when multiplied by (1-hematocrit) quantifies the fractional volume of the extracellular space. This ECV is expressed as a percentage [114].

**Fig. 15:**
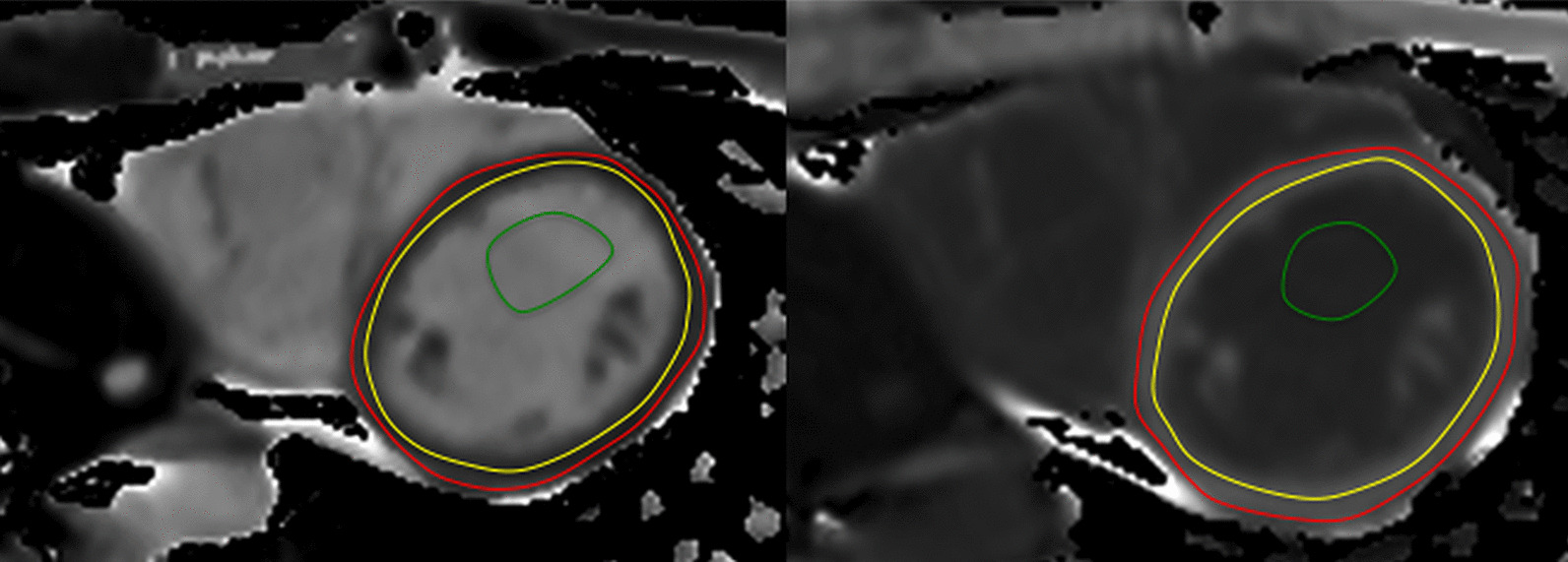
T1 maps with measurements. T1 map pre- (**a**) and post-contrast (**b**) with left ventricular endocardial and epicardial contours according to reference [119]

Offline post-processing involves manually tracing endocardial and epicardial contours [109, 115] (Fig. 15) or placing a region of interest within the septal myocardium. Inclusion of blood pool or adjacent tissue should be carefully avoided. Motion correction is generally used to correct undesired breathing motion. However, motion correction can only correct for in-plane motion and not through-plane motion. All methods, therefore, are vulnerable to partial volume effects.

### Demographic parameters

In one large study, there was no relationship of age to myocardial or blood native T1 in male subjects aged 11–69 years [116]. In female subjects there was a trend of lower native T1 with increased age (e.g. approximately 20 ms lower for females less than 45 years vs. those greater than 45 years) [116]. Female subjects < 45 years of age had a consistently higher native T1 then males, but after this age there was no difference in native T1 by gender [116]. However, other studies have failed to demonstrate a significant trend in native T1 with age or gender [117]. For ECV measurement, ECV is reported to be higher in females than males, but data are conflicting regarding the relationship of ECV with age [110, 118].

The above relationships were formally assessed in a recent meta-analysis [101]. Overall, there was no significant association between native T1 and age or percent of male participants at either 1.5 T or 3 T. However, there was a significant effect of gender with studies including more females on average having higher reported ECV values [101].

### Studies included in this review

SCMR guidelines indicate each site should establish their own site specific reference ranges for T1 mapping parameters. In the absence of such data however, the weighted mean values and reference ranges for native T1 time and ECV based on publications of at least 40 healthy subjects extracted from Table 60 are summarized in Table 61.

**Table 60:** References, native myocardial T1 relaxation time and extracellular volume fraction (ECV)

First author, year	CMR technique	n, male:female	Age range or mean ± SD (years)
Fontana, 2012 [120]	1.5 T, Siemens, ShMOLLI, ECV	27:23	47 ± 17
Kellman, 2012 [121]	1.5 T, Siemens, MOLLI, native T1 and ECV	30:32	47 ± 17
Piechnik, 2013 [116]	1.5 T, Siemens, ShMOLLI, native T1	169:173	11–69
Sado, 2013 [122]	1.5 T, Siemens, ShMOLLI, native T1	30:37	24–88
Ferreira, 2014 [123]	1.5 T, Siemens, ShMOLLI, native T1	37:13	41 ± 13
Fontana, 2014 [124]	1.5 T, Siemens, ShMOLLI, native T1	17:35	46 ± 15
Liu, 2014 [125]	3 T, Siemens, MOLLI, native T1	38:54	27–44
Puntmann, 2014 [126]	3 T, Philips, MOLLI, native T1	47 (total)	–^a^
Reiter, 2014 [118]	1.5 T, Siemens, MOLLI, native T1	20:20	20–35
aus dem Siepen, 2015 [127]	1.5 T, Philips, MOLLI, native T1 and ECV	37:19	52 ± 9
Banypersad, 2015 [128]	1.5 T, Siemens, ShMOLLI, native T1 and ECV	25:29	46 ± 15
Edwards, 2015 [129]	1.5 T, Siemens, MOLLI, native T1 and ECV	24:19	57 ± 10
Fontana, 2015 [130]	1.5 T, Siemens, ShMOLLI, native T1 and ECV	21:26	24–69
Treibel, 2015 [131]	1.5 T, Siemens, ShMOLLI, native T1 and ECV	26:24	28–69
Goebel, 2015 [132]	1.5 T, Siemens, MOLLI, native T1	31:23	18–63
Gormeli, 2016 [133]	3 T, Siemens, MOLLI, native T1	26:15	24 ± 4
Hinojar, 2016 [134]	3 T, Philips, MOLLI, native T1	9:37	42 ± 15
Ntusi, 2016 [135]	1.5 T, Siemens, ShMOLLI, native T1	53:39	44 ± 10
Rauhalammi, 2016 [136]	1.5 T and 3 T, Siemens, MOLLI, native T1	43: 41	45 ± 18
Costello, 2017 [137]	3 T, Siemens, ShMOLLI, native T1 and ECV	29:28	48 ± 15
Avitzur, 2018 [138]	3 T, Siemens, ShMOLLI, native T1	83:57	54 ± 9
Doerner, 2018 [139]	1.5 T, Philips, MOLLI, native T1 and ECV	30:20	39 ± 17
Guo, 2018 [140]	3 T, Philips, MOLLI, native T1 and ECV	18:32	36 ± 16
Ridouani, 2018 [141]	1.5 T, Siemens, MOLLI, native T1	20:20	40 ± 12
Rosmini, 2018 [142]	1.5 T, Siemens, MOLLI and ShMOLLI, native T1 and ECV	49:45	20–76
Shang, 2018 [143]	3 T, Siemens, MOLLI, ECV	45 (total)	–^a^
Yang, 2018 [144]	3 T, Siemens, MOLLI, native T1 and ECV	18:26	33 ± 16
Granitz, 2019 [145]	1.5 T and 3 T, Philips, MOLLI, native T1	26:32	42 ± 13 (male), 40 ± 14 (female)
Imran, 2019 [146]	1.5 T, Philips, MOLLI, native T1	26:25	46 ± 14
Lehmonen, 2019 [147]	1.5 T, Siemens, ShMOLLI, native T1	46 (total)	46 ± 9
Vijapurapu, 2019 [148]	1.5 T, Siemens, ShMOLLI, native T1	40:37	49 ± 14
Wan, 2019 [149]	3 T, Siemens, MOLLI, native T1 and ECV	20:20	56 ± 9

*n* number of study subjects, *MOLLI* modified look locker inversion-recovery, *ShMOLLI* short MOLLI, *T1* T1 relaxation time, *ECV* extracellular volume fraction

^a^Not provided in original publication

**Table 61:** Native myocardial T1 relaxation time and extracellular volume fraction (ECV)

Parameter	FS (T)	Vendor	Technique	n	Mean_p_	SD_p_	LL–UL^m^
Native T1 time (ms)	1.5	Siemens	MOLLI	417^a^	972	43	885–1059
	1.5	Siemens	ShMOLLI	971^b^	960	29	903–1017
	1.5	Philips	MOLLI	215^c^	989	42	905–1073
	3	Siemens	MOLLI	301^d^	1196	47	1103–1290
	3	Siemens	ShMOLLI	197^e^	1130	55	1021–1240
	3	Philips	MOLLI	201^f^	1097	66	964–1230
ECV (%)	1.5	Siemens	MOLLI	199^ g^	26	3	20–32
	1.5	Siemens	ShMOLLI	295^ h^	27	3	21–33
	1.5	Philips	MOLLI	56^i^	23	3	17–29
	3	Siemens	MOLLI	129^j^	26	3	20–32
	3	Siemens	ShMOLLI	57^ k^	25	2	20–29
	3	Philips	MOLLI	100^ l^	26	5	16–36

*ECV* extracellular volume fraction, *FS* field strength, *T* Tesla, *n* number of study subjects included in the weighted mean values, *mean*_*p*_ pooled weighted mean, *SD*_*p*_ pooled standard deviation, *LL* lower limit, *UL* upper limit, *MOLLI* modified look locker inversion-recovery, *ShMOLLI* short MOLLI, *Siemens* Siemens Medical Solutions, Erlangen, Germany, *Philips* Philips Healthcare, Best, The Netherlands

^a^Pooled weighted values from references [118, 121, 129, 132, 136, 141, 142]

^b^Pooled weighted values from references [116, 122–124, 128, 130, 131, 135, 142, 147, 148]

^c^Pooled weighted values from references [127, 139, 145, 146]

^d^Pooled weighted values from references [125, 133, 136, 144, 149]

^e^Pooled weighted values from references [137, 138]

^f^Pooled weighted values from references [126, 134, 140, 145]

^g^Pooled weighted values from references [121, 129, 142]

^h^Pooled weighted values from references [124, 128, 130, 131, 142]

^i^Values from reference [127]

^j^Pooled weighted values from references [143, 144, 149]

^k^Values from reference [137]

^l^Pooled weighted values from references [139, 140]

^m^Calculated as mean_p_ ± 2*SD_p_

## Normal values of myocardial T2 relaxation times

### CMR acquisition parameters

T2 relaxation time is the exponential time constant for the relaxation of transverse magnetization. To determine myocardial T2 time, a relaxation curve is constructed based on a CMR multi-echo pulse sequence. The most-commonly used technique utilizes a T2-preparation module followed by either a single-shot bSSFP or GRE readout [150, 151]. This technique typically acquires 3 source images with effective echo times of 0, 30 and 60 ms. 3–4 heart-beats are allowed for T1 relaxation between acquisition of source images, and data is acquired during a single breath-hold of 9–12 heart-beats. Inadequate time for complete T1-relaxation between source images can cause a T1-based bias in the T2 maps. The bSSFP technique has higher signal-to-noise but is more susceptible to off-resonance artifacts than the GRE technique. Other techniques are based on turbo-spin echo (TSE) or GRadient And Spin Echo (GRASE) acquisition modes. TSE sequences consist of a 90° excitation followed by a train of 180° refocusing pulses, with each focusing pulse producing a spin-echo with a different echo time (TE). By creating images corresponding to each echo time in the train, T2 maps can be produced by fitting the T2-signal decay equation. TSE sequences are robust to off-resonance, but they can suffer from inaccuracies due to imperfect 180° pulses which result in stimulated-echo contamination. GRASE sequences consist of a 90° excitation followed by a train of 180° pulses which produce a spin echo, and 2–4 gradient echoes. This technique is more efficient than TSE but is subject to similar biases as the TSE technique, and additionally is more sensitive to off-resonance effects due to the presence of gradient echoes and longer spacing between 180° pulses. Of note, performing multiple TSE sequences with different effective-TEs are inaccurate for determining T2 and are not recommended.

### Factors affecting T2 relaxation time

There are a number of factors which can affect the measurement of normal T2 values. Field strength has a small effect on T2 values, with 3 T scans typically having T2 values that are ~ 6 ms shorter than those obtained on 1.5 T scanners [152]. There are differences in measured T2 based on technical factors such as the type of pulse-sequence used and the vendor. The T2-preparation pulse may be sensitive to off-resonance and B1 inhomogeneity effects; these effects are more severe at 3 T. T2-preparation based on adiabatic radiofrequency (RF)-pulses have been shown to lessen these effects at 3 T. TSE and GRASE sequences are sensitive to specifics of the RF-pulses which are vendor and implementation dependent. Similar to T1 mapping, it is imperative to standardize local pulse sequence parameters. As for T1 mapping, site-specific reference ranges should be established.

### CMR analysis methods

T2 is the relaxation time (in milliseconds) of the transverse magnetization. Similar to T1 assessment, to generate parametric maps of T2, the source images typically need to be aligned using non-rigid registration. Again, these techniques can correct  for in-plane motion but not through-plane motion. Both off-line and on-line techniques have been used as for T1 mapping.

### Demographic parameters

Published data on T2 values have sample sizes smaller than those of T1 methods. Thus, effects of demographic parameters in relationship to T2 times are not well established. One paper using GRASE demonstrated a slightly higher native T1 in females as compared to males (56.7 ms vs 54.6 ms; p = 0.008) at 1.5 T but no difference at 3 T. No significant differences in T2 were seen as a function of age [145]. Another study showed no difference between male and female subjects when controlling for age, but did see a trend of lower T2 with increasing age [153]. Another study using T2-prepared bSSFP at 3 T demonstrated no significant differences in T2 by age or gender [154].

### Studies included in this review

SCMR guidelines indicate each site should establish their own site specific reference ranges for T2 mapping parameters. In the absence of such data however, the weighted mean values and reference ranges for T2 on publications of at least 40 healthy subjects (combined males and females) are shown in Table 62.

**Table 62:** Myocardial T2 relaxation time (ms)

First author, year	FS (T)	Vendor	Technique	n, male:female	Age range or mean ± SD (years)	Mean	SD	LL–UL^a^
Wassmuth, 2013 [155]	1.5	Siemens	T2P bSSFP	60:13	20–70	55	5	45–65
Wassmuth, 2013 [155]	1.5	Siemens	FLASH	60:13	20–70	52	5	42–62
Hinojar, 2016 [134]	3	Philips	GSE	9:37	42 ± 15	45	4	37–53
Ridouani, 2018 [141]	1.5	Siemens	T2P bSSFP	20:20	40 ± 12	51	3	45–57
Granitz, 2019 [145]	1.5	Philips	GSE	26:32	40 ± 14 (male), 42 ± 13 (female)	56	3	50–62
Granitz, 2019 [145]	3	Philips	GSE	26:32	40 ± 14 (male), 42 ± 13 (female)	52	3	46–58

*FS* field strength, *T* Tesla, *n* number of study subjects, *SD* standard deviation, *LL* lower limit, *UL* upper limit, *T2P* T2 preparation, *bSSFP* balanced steady state free precession, *FLASH* fast low angle shot, *GSE* gradient spin echo, *Siemens* Siemens Medical Solutions, Erlangen, Germany, *Philips* Philips Healthcare, Best, The Netherlands

^a^Calculated as mean ± 2*SD

## Normal values of myocardial T2* relaxation time

### CMR acquisition parameters

Quantification of the T2* relaxation time plays an important role for estimation of myocardial iron overload [156]. T2* time is also altered in myocardial necrosis and hemorrhage [102]. For quantification of the myocardial T2* time, the gradient-echo T2* technique with multiple increasing echo times is preferred over the spin-echo T2 technique due to a greater sensitivity to iron deposition [157–159]. According to the current consensus statement by the SCMR, a dark-blood multi-echo gradient echo sequence with 8 equally spaced echoes between 2 and 18 ms should be used for T2*-mapping at 1.5 T [102]. Usually a single-breath hold technique is used. Normal values and a grading system for myocardial iron overload are available for 1.5 T [158].

### CMR analysis methods

**Fig. 16:**
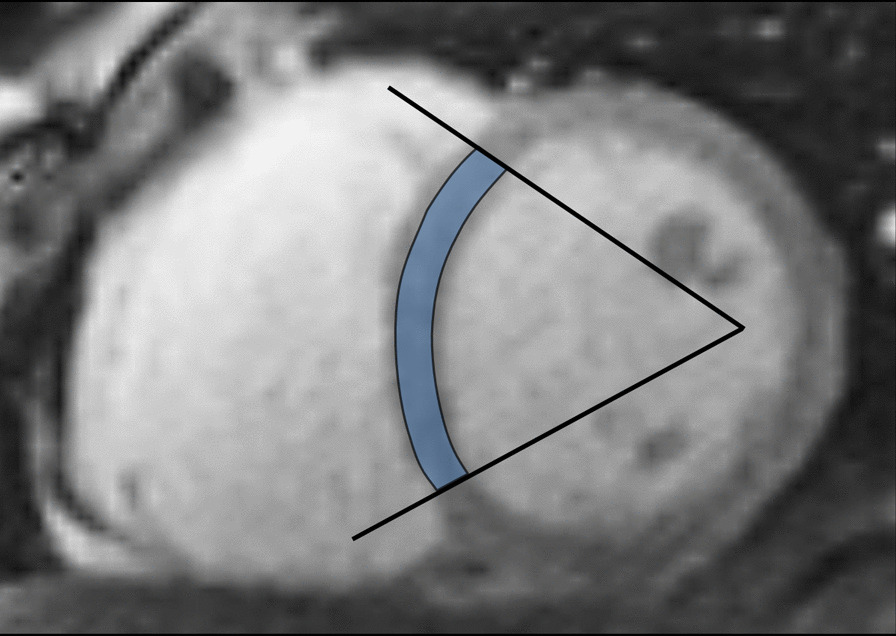
Measurements of myocardial T2* are obtained in the septum

Gradient-echo T2* images are vulnerable to distortions of the local magnetic field e.g. by air-tissue interfaces. The myocardial septum is surrounded by blood on both sides, so susceptibility differences are less than in the lateral wall with improved image quality on T2* images. Therefore, T2* measurements are obtained by placing a region of interest on the interventricular septum of a midventricular short axis slice [102, 159] (Fig. 16).

T2* times are frequently reported as relaxation rate, representing the reciprocal of the time constant and calculated as R2* = 1000/T2*. The units of R2* is s^−1^ [159]. Cardiac iron concentration can be calculated from T2* values by the following equation: [Fe] = 45 / (T2*)^1.22^, where [Fe] is the cardiac iron concentration in milligrams per gram dry weight and T2* in milliseconds [160].

### Demographic parameters

T2* of the myocardium is not related to age [161]. To our knowledge the relationship between other demographic parameters and T2* has not been assessed.

### Studies included in this review

The mean T2* of the myocardium (interventricular septum) is approximately 36 ms [161] at 1.5 T using a multi-echo GRE sequence. T2* > 20 ms is considered within the range of normal.

Depending on the risk to develop heart failure as a consequence of myocardial iron overload, a grading system for disease severity has been published (Table 63) [102, 156, 162].

**Table 63:** Grading of iron overload based on T2* measurements at 1.5T according to [102, 156, 162]

Iron overload	T2* (ms)
Normal	> 20
Iron overload	10–20
Severe iron overload	< 10

## Regional measurements and cardiac strain

### CMR acquisition parameters

A number of imaging methods have been developed to acquire cardiac strain information from cine CMR. These methods include tagged cine CMR, PC-CMR, velocity encoded CMR, displacement encoding with stimulated echoes (DENSE), and strain-encoding (SENC) [163, 164]. Tagged CMR is a widely validated reproducible tool for strain estimation. The method is used in clinical studies and is considered the reference standard for assessing regional function [165, 166]. Recently feature-tracking CMR (FT-CMR) has been increasingly reported due to compatibility with existing cine CMR images [167].

### CMR analysis methods

Cardiac strain is a dimensionless measurement of the deformation that occurs in the myocardium. Cardiac strain can be reported as three normal strains (circumferential, radial, and longitudinal) and six shear strains—the angular change between two originally mutually orthogonal line elements, with the more clinically investigated shear strain and the circumferential-longitudinal shear strain (also known as torsion). They can also be computed as fiber and cross-fiber strains which require anatomical knowledge of fiber architecture, or as principal strains along the principal stretching and shortening directions [168]. Here, we concentrate on the widely reported circumferential and longitudinal strains. Although frequently reported, radial strain is less reproducible because of the reduced resolution of imaging in the radial direction as opposed to the circumferential or longitudinal directions.

There are a number of different methods to quantify strain: registration methods, feature-based tracking methods, deformable models, Gabor Filter Banks, optic flow methods, harmonic phase analysis (HARP) [169], and local sine wave modeling (SinMod) [163]. Technical review papers for these methods can be found in the following literature [167, 170–172].

**Fig. 17:**
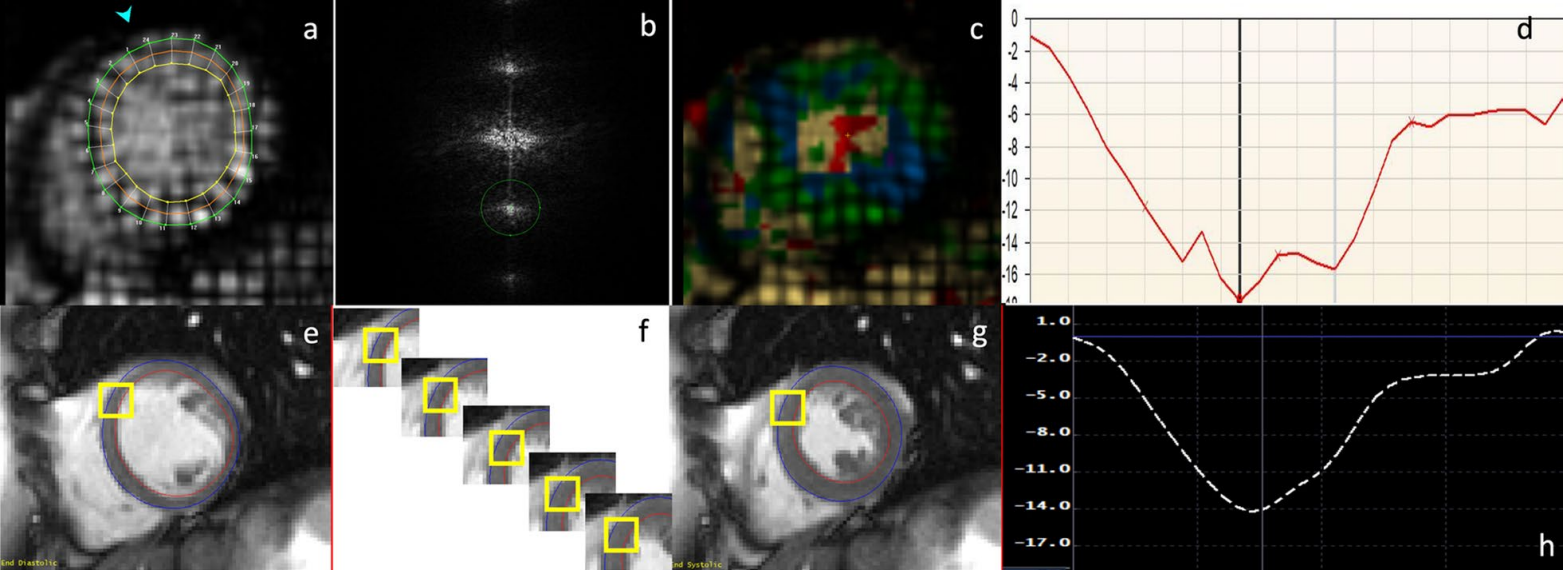
Illustration of strain computation using the Harmonic Phase (HARP) tool on tagged CMR images (**a**–**d**) and from feature tracking on cine CMR images (**e**–**h**). In HARP, first a semi-automated frequency analysis of the tagged CMR image (**a**) is performed to identify the harmonic peaks in each of the tag directions (**b**), filters are then applied to isolate the peaks and obtain the corresponding phase maps from which Eulerian strain maps (**c**) can be computed. Subplot (**d**) shows the strain curve at the mid-ventricular level for an asymptomatic volunteer obtained based on tracking of the user-defined mesh (**a**). In feature tracking of cine CMR images, endo- and epicardial contours are drawn at end-diastole (**e**) or end-systole (**g**). A characteristic pixel pattern in the order of a few millimeters squared is identified as a template. The software then tries to discern a similar pattern in the subsequent frame from which displacement of the pixels is computed (**f**). This is repeated through the entire cycle to obtain displacement from which strain is computed. Subplot (**h**) shows the strain curve at the mid-ventricular level computed from feature tracking. The tagged and cine CMR images and the strain curves were from the same participant

HARP is one of the most widely reported and validated methods for analyzing tagged CMR for cardiac strain, in part due to its large scale use in the MESA study [169, 173]. Strain patterns are reported according to the 16 or 17 segment AHA model. Consistent manual tracing of the endocardial and epicardial contours is necessary to reproducible strain results. With tagged CMR, midwall strain is preferred to epicardial and endocardial strain to maximize the amount of tagging data available for strain calculations [172, 174]. With HARP analysis such as that used in the MESA trial [169], careful selection of the first harmonic is necessary. Figure 17a shows an outline of tagged CMR analysis using HARP.

**Fig. 18:**
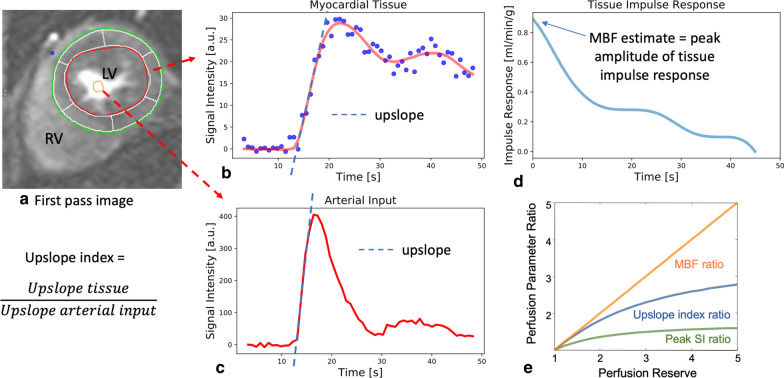
**a** The quantification of myocardial perfusion proceeds from the segmentation of images acquired during the first pass of contrast through the heart to delineate myocardial segments and a region in the center of the LV blood pool for the arterial input. This example shows one short-axis image for a mid-slice LV level. **b** For each myocardial segment one obtains a signal-intensity versus time curve. A useful semi-quantitative parameter for the assessment of the perfusion in a myocardial segment is the upslope, which is estimated from a fit to approximately 3–5 points during the initial myocardial contrast enhancement. **c** An analogous upslope parameter can be extracted from the first pass peak of the arterial input function. A perfusion index can be calculated from the ratio of the two upslopes as shown in the formula below (**a**), and accounts for some changes in the arterial input between rest and stress. **d** Absolute estimates of myocardial blood flow in ml/min/g can be obtained from the myocardial contrast enhancement curves and the arterial input function by fitting to a kinetic model for contrast enhancement, or, as done for this example, to estimate the myocardial impulse response by constrained deconvolution. Constraints are that the impulse response should be a monotonically decaying function of time, and requiring a relatively smooth, “regularized” impulse response. Myocardial blood flow (MBF) is estimated from the peak amplitude of the impulse response. **e** The ratio of myocardial blood flows during stress, divided by MBF at rest provides the most accurate estimate of the coronary flow reserve. In comparison, other ratios of perfusion indices (e.g. upslope index) for stress and rest systematically underestimate the flow reserve but may still prove useful for the detection of disease, assuming that one has established the normal range of the index.

FT-CMR has shown diagnostic and prognostic utility across a variety of pathologies. Currently, FT-CMR software from TomTec (TomTec Imaging Systems, Unterschleissheim, Germany), QStrain (Medis Medical Imaging Systems, Leiden, The Netherlands) and CVI42 (Circle Cardiovascular Imaging Inc., Calgary, Canada) are widely used in clinical research for calculation of LV strains. Similar to tagged CMR from HARP, strains are reported in the 16 or 17 segment models.

Segmentation of the myocardium (either semi-automated or completely automated) at the start of the cardiac cycle is an essential first step across all software. The software records a characteristic pixel pattern (an area of pixels typically in the order of 10–15 mm^2^) in the reference frame; an area with an identical pixel pattern is recognized in the next frame that maximizes certain similarity metrics [167, 175]. This procedure is repeated for all pixels in each image and for each frame to track the borders throughout the whole cardiac cycle with constraints for smoothness of the deformation field applied. FT-CMR tracking quality is largely governed by the quality and resolution of the pixels at the endocardial and epicardial borders which are feature-rich as compared to the mid-myocardium. This method has been extended to volume data by tracking voxels to obtain 3D FT-CMR [176]. Figure 17b shows an outline of the concept underlying strain analysis by FT-CMR.

### Demographic parameters

Using both tagged CMR and FT-CMR, several studies report greater age is associated with decrease in peak circumferential or longitudinal shortening [176–178]. In tagged CMR and a few FT-CMR reports, gender also affects normal values. Cardiac strain values for women are higher than those of men [66, 176, 179–181]. However, some FT-CMR reports showed no association of circumferential or longitudinal strains with age or gender [26, 177].

### Studies included in this review

Several studies have presented cohorts for determining normal LV strain. For the purpose of this review, only cohorts of 40 or more normal subjects using SPAMM (spatial modulation of magnetization tagging) or FT-CMR have been included. Inclusion criteria include a full description of the subject cohort (including the analysis methods used), age and gender of subjects. Table 64 represents a summary of publications reporting normal values for strain that fit the criteria. We have only included reference values for global values of strain as the inter-reader and inter-study reproducibility of regional strain values vary widely between published reports.

**Table 64:** References, myocardial strain

First author, year	CMR technique	n, male:female	Age range (years)
Neizel, 2009 [182]	1.5 T, 3 short axis images, tagged CMR (SPAMM), tagged resolution 7 mm; HARP method^b^	40:35	22–69
Augustine, 2013 [177]	1.5 T, short axis stack bSSFP, 2, 3, and 4 chamber bSSFP; feature tracking (TomTec software^c^)	54:62	(30 ± 8)^a^
Venkatesh, 2015 [183]	1.5 T, 3 short axis images, tagged CMR (SPAMM), tagged resolution 7 mm, HARP method^b^	46:83	45–84
Andre, 2015 [179]	1.5 T, short axis stack, 2, 3, and 4 chamber bSSFP; feature tracking (TomTec software^c^)	75:75	21–71
Cai, 2017 [66]	3 T, short axis stack, 2, 3, and 4 chamber bSSFP; feature tracking (CVI42 software^d^, 2D)	91:89	20–69
Liu, 2018 [26]	1.5 T, short axis stack bSSFP, 2, 3, and 4 chamber bSSFP; feature tracking (CVIR42 software^d^, 3D)	50:50	20–70
Peng, 2018 [176]	1.5 T and 3 T, short axis stack, 2, 3, and 4 chamber bSSFP; feature tracking (QStrain software^e^)	75:75	18–82

*n* number of study subjects, *SPAMM* spatial modulation of magnetization, *HARP* harmonic phase, *bSSFP* balanced steady state free precession

^a^Mean ± SD (age-range not provided in original publication)

^b^HARP commercial, Diagnosoft, Palo Alto, CA, USA

^c^TomTec Imaging Systems, Unterschleissheim, Germany

^d^CMI42, Circle Cardiovascular Imaging Inc., Calgary, Canada

^e^QStrain, Medis Medical Imaging Systems, Leiden, The Netherlands

With tagged CMR, normal midwall circumferential strain values are relatively comparable between studies [182, 183] (Table 65). With 2D FT-CMR, small differences between published results exist for reference values, probably due to inter-vendor differences [26, 66, 177, 179]. The reference ranges of normal circumferential strains from FT-CMR (Table 66) are comparable to those obtained from tagged CMR (Table 65). Strain values are traditionally reported as more negative values meaning greater contractility. For both global circumferential and global longitudinal strain, a strain value of approximately -14% is the limit of normal; values more positive than this are considered to be abnormal.

**Table 65:** Left ventricular global peak circumferential strain using tagging

Parameter	n	Mean_p_	SD_p_	LL–UL^a^
Circumferential strain (%)	204	− 20.1	3.0	− 26.0 to − 14.2

Pooled weighted values from references [182, 183]

*n* number of study subjects included in the weighted mean values, *mean*_*p*_ pooled weighted mean, *SD*_*p*_ pooled standard deviation, *LL* lower limit, *UL* upper limit

^a^Calculated as mean_p_ ± 2*SD_p_

**Table 66:** Left ventricular global peak circumferential and longitudinal strain for men and women using 2D feature tracking

Parameter	Men				Women			
	n	Mean_p_	SD_p_	LL–UL^a^	n	Mean_p_	SD_p_	LL–UL^a^
Circumferential strain (%)	295	− 20.9	3.2	− 27.2 to − 14.6	301	− 22.7	3.3	− 29.2 to − 16.2
Longitudinal strain (%)	295	− 19.4	3.3	− 26.1 to − 12.7	301	− 21.4	3.6	− 28.7 to − 14.2

Pooled weighted values from references [66, 176, 177, 179]

*n* number of study subjects included in the weighted mean values, *mean*_*p*_ pooled weighted mean, *SD*_*p*_ pooled standard deviation, *LL* lower limit, *UL* upper limit

^a^Calculated as mean_p_ ± 2*SD_p_

Given the inclusion criteria noted above, one publication [176] used 3D FT-CMR (Table 67). The mean values and reference ranges were lower compared to 2D FT-CMR and tagged-CMR.

**Table 67:** Left ventricular global peak circumferential and longitudinal strain using 3D feature tracking according to reference [26]

Parameter	n	Mean	SD	LL–UL^a^
Circumferential strain (%)	100	− 17.6	2.6	− 22.8 to − 12.4
Longitudinal strain (%)	100	− 14.6	2.7	− 20.0 to − 9.2

*n* number of study subjects, *SD* standard deviation, *LL* lower limit, *UL* upper limit

^a^Calculated as mean ± 2*SD

## Myocardial perfusion

Myocardial perfusion has been quantified with T1-weighted dynamic imaging during the first pass of a contrast bolus by semi-quantitative methods that derive dimensionless indices (e.g. the upslope of myocardial signal intensity changes during initial contrast enhancement). Alternatively, absolute estimates of MBF may be determined (in units of ml per g of tissue and per minute (ml/g/min)). To derive absolute measures of blood flow, the CMR signal intensity changes must be converted to be linearly proportional to contrast concentration. This assumption may not hold true at high contrast concentrations (e.g. in the blood pool). Instead, low-dose bolus injections of contrast agent (e.g. < ~ 0.04 mmol/kg of Gd-DTPA) with saturation correction [184] or special pulse sequences are used for CMR perfusion (e.g. “dual-sequence” [185], dual-bolus techniques [186, 187]) to provide linear measures of gadolinium concentration.

### CMR acquisition parameters

Normal values for quantitative myocardial perfusion measures have been obtained by ECG-gated, T1-weighted dynamic imaging during the first pass of an injected contrast bolus using gradient-echo image readouts without or with bSSFP. Echo-times are kept as short as possible for any of these image acquisition methods to minimize T2*-related signal loss. T1-weighting is generally maximized by using saturation-recovery magnetization preparations. Semi-quantitative parameters depend on contrast dosage and injection protocol, sequence technique and acquisition parameters. The normal ranges for semi-quantitative parameters should therefore only be used as reference when the same protocol settings are employed. MBF (in units of mL/min/g) should be independent of the specific perfusion imaging protocol settings. However, a specific technique may still introduce a bias to under or over-estimate MBF.

In clinical use, myocardial perfusion imaging is generally performed at rest and during vasodilator stress. Adenosine and regadenoson are currently the most frequently used pharmacological stress agents for myocardial perfusion imaging and have supplanted dipyridamole in this role. Adenosine and regadenoson have similar hemodynamic effects on coronary artery blood flow [188]. The choice of pharmacologic stress agent is mostly determined by considerations of patient comfort, safety and cost. Regadenoson is more expensive, but better tolerated than adenosine. A unique application of myocardial perfusion imaging is its use in combination with the cold-pressor test to assess coronary endothelial function [189, 190].

### CMR analysis methods

All quantitative approaches for CMR myocardial perfusion are based on signal-intensity versus time curves that depict the contrast enhancement during the first pass and recirculation of an injected contrast bolus. The myocardial perfusion images are segmented along the endo- and epicardial borders, and the ventricular wall is divided into segments following a standardized segmentation model for cardiac perfusion studies (Fig. 18).

The most widely used *semi-quantitative* parameter has been the up-slope parameter for initial myocardial contrast enhancement. Because the upslope derives from signal-intensity curves with arbitrary units, the value of the up-slope depends on the image acquisition settings and on the characteristics of the contrast bolus. For this reason, the myocardial up-slope parameter is generally normalized by the up-slope of the arterial blood pool of the LV to obtain a dimensionless perfusion index. This index is quantified during resting conditions and “stress” at maximal vasodilation (i.e. hyperemia) after infusion of a pharmacological agent (e.g. adenosine). The coronary flow reserve is the ratio of the “stress” index, divided by “rest” index. We refer below to this parameter as the “up-slope” perfusion reserve.

Absolute quantification of the myocardial perfusion reserve entails estimating MBF in ml/min/g. MBF quantification can be based on tracer-kinetic modeling, or by using a deconvolution technique that is based on Zierler’s central volume theorem [191]. In either case it is important to have an accurate depiction of the arterial input of contrast to a myocardial region of interest, which in practice is approximated by the arterial contrast enhancement observed in the LV cavity. The myocardial perfusion reserve is estimated as “stress” MBF, divided by “rest” MBF. Nevertheless, hyperemic MBF by itself is also a useful measure of the maximal vasodilator response and its normal range is also provided by some studies in the literature. Rest MBF increases in proportion to the cardiac workload, and the rate-pressure product (RPP) is used as measure of cardiac workload to provide an RPP-normalized MBF value (rest MBF/RPP), whose normal range is narrower in healthy persons than for the rest MBF without any adjustment for RPP.

### Demographic parameters

In the CMR study by Wang et al. [192], rest MBF was higher in women than in men; this agrees with previous studies in healthy subjects using positron emission tomography [193]. Men have a lower hyperemic MBF compared to women, with adjustment for coronary heart disease risk factors [192]. Although male sex carries a higher risk for coronary heart disease, few studies of myocardial perfusion in healthy subjects have considered gender-related differences in MBF. The coronary flow response to the cold-pressor test is also higher in women compared to men [194].

### Studies included in this review

There are two publications reporting reference values of absolute MBF at rest and under pharmacological stress with a sufficient sample size (> 40 healthy subjects) (Table 68). The original study published by Wang et al. included subjects from the MESA population with comorbidities such as hypertension and diabetes [192]. However, for the purpose of this review, a re-analysis of [192] was performed for a subset of 99 healthy subjects of the cohort by one of the authors (MJH). Values are given for the entire cohort and for men and women separately. In the other study by Brown et al. reference ranges are presented for the entire cohort of 42 healthy subjects [195]. Although in both studies images were acquired by means of a T1 weighted saturation recovery prepared single-shot GRE sequences, normal reference ranges differ substantially. Therefore, in this review we abstained from calculation of weighted mean values and present reference ranges given in the two publications separately (Table 69).

**Table 68:** References, normal absolute myocardial blood flow at rest and stress and perfusion reserve in adults and children

First author, year	CMR technique	n, male:female	Age mean ± SD (years)
Wang, 2006 [192] ^a^	1.5 T, T1weighted saturation recovery single-shot GRE, at rest and under adenosine stress	49:50	59 ± 11
Madriago, 2015 [196]	3 T, T1weighted saturation recovery single-shot GRE, at rest and under adenosine stress	11:9	8 ± 5
Brown, 2018 [195]	3 T, T1weighted saturation recovery single-shot GRE, at rest and under adenosine stress	19:23	23 (22–29)^b^

*n* number of study participants, *GRE* gradient echo

^a^Analysis of a subset of healthy subjects (without hypertension, no use of antihypertensive or other medication for a cardiovascular condition, no diabetes, normal glucose tolerance, no smoking history and normal total cholesterol (< 240 mg/dl)) of the original cohort

^b^Median (interquartile range)

**Table 69:** Reference ranges of normal absolute myocardial blood flow (MBF) at rest and during adenosine stress and perfusion reserve in adults and children

References	n	MBF at rest (ml/min/g)	MBF during Adenosine stress (ml/min/g)	Perfusion reserve^a^
		Mean ± SD (LL–UL)^b^	Mean ± SD (LL–UL)^b^	Mean ± SD (LL–UL)^b^
Wang, 2006 [192]	99 49 50	All: 1.02 ± 0.24 (0.54–1.5) Men: 0.96 ± 0.23 (0.5–1.96) Women: 1.08 ± 0.23 (0.62–2.32)	All: 3.13 ± 0.80 (1.53–6.19) Men: 2.79 ± 0.72 (1.35–5.49) Women: 3.46 ± 0.73 (2.02–7.50)	All: 3.17 ± 0.87 (1.43–4.91)
Madriago, 2015 [196] ^c^	20	0.94 ± 0.17 (0.6–1.28)	2.34 ± 0.82 (0.7–3.98)	2.63 ± 0.96 (0.71–4.55)
Brown, 2018 [195]	42	0.65 ± 0.13 (0.39–0.91)	2.71 ± 0.61 (1.49–3.93)	4.24 ± 0.69 (2.86–5.62)

*n* number of study participants, *MBF* myocardial blood flow

^a^Ratio of MBF during stress divided by MBF at rest

^b^Calculated as mean ± 2*SD

^c^Data table was made available by senior author to calculate mean and SD

There is a single study presenting reference ranges of myocardial perfusion in children [196] (Table 68). Although the sample size is small (n = 20) and children have cardiovascular pathologies (e.g. atrial and ventricular septal defects), data is presented here since a study of myocardial stress perfusion imaging in a larger subset of entirely healthy children seems not feasible (Table 68).

## Artificial Intelligence (AI)-based segmentation methods for analysis of cine MRI

Currently no AI-based normal values have been published in the literature. In recent years however, major improvements have been made in the development of automated CMR segmentation methods based on AI technology using so called Convolutional Neural Networks (CNN). Most published work report methods for automated LV and RV segmentation in Cine CMR [3, 197–203]. CNN based methods have also been presented for automated quantification of atrial dimensions [2, 204], myocardial scar tissue from LGE [205, 206], T1 mapping [207], aortic flow [208] and disease classification [209]. Given the potential importance of this topic to the field of CMR, this section summarizes relevant literature in this area and provides a summary of the publicly available CMR data sets relevant to AI segmentation of CMR data.

CNN-based automated image segmentation methods rely on training data, i.e. images with known segmentation result, to derive a neural network with multiple nodes, layers and weighting parameters that can be applied to unseen images to label every pixel in the image. The structure of the neural network varies among publications, but many are based on or are similar to the UNET structure introduced by Ronnenberger et al. in 2015 [210]. The applicability of a CNN implementation is highly dependent on the data that was used to train the weights in the CNN. Ideally, the training data is representative for the data for which the CNN is to be used. Important considerations include the mix of pathologies, mix of CMR scanner vendors and variation in CMR acquisition parameters in the training set. Many published CCN methods for CMR image segmentation are based on data sets that have been made publicly available in the setting of so called challenges, i.e. competitions in which participants are invited to develop the best segmentation algorithm for a given type of data [211]. Table 70 lists the most relevant public CMR Cine CMR data sets that have been used for this purpose.

**Table 70:** Publicly available data sets that have been used for training and testing for automated segmentation algorithms of the left and right ventricle

Data set	Conference/source	n	Segmented structure	Data description
MICCAI-2009 [213]	MICCAI 2009	45	Left ventricle	Single center, single vendor 5 sub-groups: healthy, hypertrophy, heart failure with infarction and heart failure without infarction Data hosted on: https://www.cardiacatlas.org/studies/sunnybrook-cardiac-data/
LVSC-2011 [214]	STACOM-2011	200	Left ventricle	Multi center, multi-vendor Myocardial infarction
RVSC-2012 [215]	MICCAI 2012	48	Right ventricle	Single center Randomly selected clinical cases
ACDC-2017 [198]	MICCAI 2017	150	Left ventricle	Single center, 2 scanners, 1 vendor 5 sub-groups (Normal, post-myocardial infarction, dilated cardiomyopathy, hypertrophic cardiomyopathy, abnormal right ventricle)
KAGGLE-2015 [216]	KAGGLE 2015s annual data science bow	1100	Left ventricle	Multi center, multi scanner Mix of patient and volunteer scans Only end-diastolic and end-systolic ground truth results provided. No gold standard segmentations available
Multiple sources	Cardiac Atlas Project [217]	> 6500	Left ventricle	Multi center, multi-vendor Asymptomatic subjects Data acquired with gradient echo cine acquisition Data hosted on: https://www.cardiacatlas.org/studies/mesa/

*n* number of subjects, *MICCAI* Medical Image Computing and Computer Assisted Intervention, *LVSC* Left Ventricle Segmentation Challenge, *STACOM* Statistical Atlases and Computational Modelling of the Heart, *RVSC* Right Ventricle Segmentation Challenge, *ACDC* Automatic Cardiac Diagnosis Challenge

Validation of CNN based segmentation methods is based on comparing the results of automated segmentation with manual results from a trained observer. Commonly used geometrical validation metrics include the Dice overlap, Haussdorf distance and average distance between contours [198]. Additionally, derived quantitative parameters from either automated or manual contours can be compared. As manual analysis is subject to observer bias and variability it can only serve as surrogate gold standard. Some papers report the observer variability of manual analysis in order to assess how the limits of agreement of an automated method compare with the limits of agreement within or between manual observers.

CNN-based image segmentation methods are being introduced in commercially available image analysis software packages. The question arises whether results from such automated methods can be used interchangeably with results from manual image analysis. Although CNN’s are designed to replicate the image segmentation performed by an expert observer, it is conceivable that relevant differences may occur, especially in myocardial pathologies which were not well represented in the cohort that was used to train the CNN.

### Studies included in this review

Table 71 lists 11 studies presenting CNN based image segmentation methods for automated analysis of CMR imaging data. Studies are included based on having either used a well described public data set for training and testing, or a dataset of > 300 subjects selected according to a properly described inclusion protocol. Most published work have used publicly available datasets for training and testing of algorithms for LV [198–202, 204] or RV [198, 200, 202–204] segmentation in short-axis cine CMR. The use of public data sets for algorithm training and validation enables objective comparison of the performance of the methods. Due to the relatively small size of training sets used and the limitation in variation in patient pathology, scanner manufactures, field strength and scanning protocol, it is uncertain how these methods perform on routine clinical CMR data. However, the above studies do convincingly demonstrate the high potential of CNN based image segmentation.

**Table 71:** Recent studies describing fully automated LV or RV segmentation algorithm based on Convolutional Neural Networks (CNN)

Author, year	Segmented structure	Data used for training/validation	Validation methods/remarks
Tran, 2017 [203]	LV + RV	LV: MICCAI-2009 (n = 45) RV: RVSC-2012 (n = 45)	Validation: auto vs manual Metrics: DICE, HD, ACD
Bai, 2018 [2]	LV + RV Including LV, RV, LA, RA from long-axis cine	4875 subjects UK Biobank cohort Multi-center, single vendor	Validation: auto vs manual Metrics: DICE, HD, APD LV: EDV, ESV, EF, SV, CO, LV mass RV: EDV, ESV, EF, CO
Bernard, 2018 [198]	LV + RV	ACDC-2017 (n = 150)	Nine methods compared Validation: auto vs manual Metrics: DICE, HD, CLBR LV: EDV, ESV, EF, LV mass RV: EDV, ESV, EF
Khened, 2018 [200]	LV + RV	KAGGLE-2015 (n = 1140) ACDC-2017 (n = 150) LVSC-2011 (n = 200)	Validation: auto vs manual Metrics: DICE, CLBR Patient diagnosis
Tan, 2018 [201]	LV	LVSC-2011 (n = 200) KAGGLE-2015 (n = 1140)	Validation: auto vs manual Metrics: DICE, JI, HD EDV, ESV
Tao, 2018 [3]	LV	Training: 400 subjects Testing: 150 subjects Multi-center, multi-vendor Multiple patient categories: MI (n = 322), DCM (n = 168), HCM (n = 23), DCM (n = 23), PH (n = 10) other (n = 27), normal (n = 23)	Validation: auto vs manual Metrics: DICE EDV, ESV, EF, LV mass
Vigneault, 2018 [204]	LV + RV from multiple views	53 subjects HCM (n = 42), healthy (n = 21) ACDC-2017 (n = 150)	Validation: auto vs manual Metrics: DICE
Backhaus, 2019 [212]	LV + RV	Evaluation of SuiteHEART software (Neosoft) 300 randomly selected patients used for validation Single center 1.5 T and 3 T data	Validation: auto vs manual LV: EDV, ESV, SV, EF, LV mass RV: EDV, ESV, EF
Bhuva, 2019 [197]	LV	Training data: 599 subjects Test data 110 patients, 5 disease categories: myocardial infarction (n = 32), LVH (n = 17), cardiomyopathy (n = 17), other pathology (n = 14), healthy volunteers (n = 30) Multi-center, multi-vendor, 1.5 T + 3 T Scan-rescan data Data availability: https://www.thevolumesresource.com	Validation based on comparing scan-rescan reproducibility of automated vs manual analysis EDV, ESV, SV, EF, LV mass Detectable change in EF
Curiale, 2019 [199]	LV	MICCAI-2009 (n = 45) Cardiac Atlas Project (n = 95)	Validation: auto vs manual Metrics: DICE EDV, ESV, EF, LV mass
Tong, 2019 [202]	LV + RV	ACDC-2017 (n = 150)	Validation: auto vs manual Metrics: Dice, HD LV: EDV, ESV, EF, LV mass RV: EDV, ESV, EF

Recent studies (> 2017) describing fully automated LV or RV segmentation algorithm based on Convolutional Neural Networks (CNN), which were validated either on publicly available data sets, or using lager (> 300 subjects) single-center or multi-center clinical patient cohorts. Segmentation is performed from short-axis cine MR, except stated otherwise.

*LV* left ventricle, *RV* right ventricle, *LA* left atrium, *RA* right atrium, *MI* myocardial infarction, *DCM* dilated cardiomyopathy, *HCM* hypertrophic cardiomyopathy, *PH* pulmonary hypertension, *DICE* dice overlap metric, *HD* Hausdorff distance, *JI* Jaccard index, *CLBR* challenge leader board ranking, *ACD* average contour distance, *MICCAI* Medical Image Computing and Computer Assisted Intervention, *LVSC* Left Ventricle Segmentation Challenge, *ACDC* Automatic Cardiac Diagnosis Challenge.

There are several studies for which AI methods were developed and applied to larger cohorts of subjects. Bai et al. presented a CNN method that was trained on a large dataset of 4875 subject scans of the UK BioBank cohort [2]. This method provides automated segmentation and quantification of short-axis and long-axis cine CMR for all four heart chambers. It was shown that the method provides excellent segmentation results when applied to cases from the UK Biobank cohort. However, for application in clinical patients, the method demonstrated sub-optimal performance. Retraining the network by including additional cases of a clinical cohort did result in better results in patient data.

In the study of Tao et al. multi-center, multi-vendor, multi-pathology data was used to train and test vendor specific CNNs and a mixed-vendor CNN [3]. The authors showed that the CNN trained using a mix of data from all centers, vendors and pathologies had the highest overall performance. This indicates that it is feasible to use the same optimally trained CNN across multiple centers, vendors and patient pathologies.

A retrospective clinical validation of a commercial image analysis software tool was presented by Backhaus et al. [212]. In a randomly selected cohort of 300 CMR examination LV and RV parameters were automatically derived using a commercial software tool (SuiteHEART, NeoSoft, Pewaukee, Wisconsin, USA) incorporating CNN based image segmentation. The agreement between manual and automated LV parameter assessment was good (Bias in LV-EF: − 2.5% ± 5.9%), while for RV assessment the agreement was lower (Bias in RV EF: 5.8% ± 9.6%). As expected, the agreement between manual and automated analysis was lowest in cases of poor image quality and in patients with abnormal cardiac anatomy.

Bhuva et al. used another approach to assess the performance of CNN based image segmentation as compared to manual analysis [197]. In their study a CNN LV segmentation method was trained on 599 subjects and tested on scan-rescan data of 110 patients with multiple pathologies. It was shown that automated image segmentation yielded similar scan-rescan reproducibility as manual image analysis, which suggests that automated segmentation is a viable alternative to manual analysis in a clinical setting.

## Conclusions

CMR enables quantification of various functional and morphological parameters of the cardiovascular system. Advantages of a quantitative evaluation are a better differentiation between pathology and normal conditions, grading of pathologies, monitoring changes under therapy, and evaluating prognosis and the possibility of comparing different groups of patients and normal subjects.

Hence, here we present an updated and expanded version of the “normal value CMR review”. This review has provided reference values and factors affecting these parameters on current CMR techniques and sequences. Due to continuing publications in the field and new techniques transferred from research tools into clinical practice existing reference ranges need to be updated and values for new techniques integrated.

## Data Availability

Not applicable.

## Abbreviations

AA: Ascending aorta
ACDC: Automatic Cardiac Diagnosis Challenge
AHA: American Heart Association
AI: Artificial intelligence
ARVC: Arrhythmogenic right ventricular cardiomyopathy
AVPD: Atrioventricular plane descent
BMI: Body mass index
BSA: Body surface area
bSSFP: Balanced steady state free precession
CE: Contrast enhanced
Ch: Chamber
CI: Cardiac index
CLBR: Challenge leader board ranking
CO: Cardiac output
CMR: Cardiovascular magnetic resonance
CMRA: Cardiovascular magnetic resonance angiography
CNN: Convolutional neural network
DENSE: Displacement encoding with stimulated echoes
DCM: Dilated cardiomyopathy
DICE: Dice overlap metric
ECG: Electrocardiogram
ECV: Extracellular volume
EDV: End-diastolic volume
EF: Ejection fraction
ESV: End-systolic volume
FD: Fractal dimension
FT: Feature-tracking
FGRE: Fast gradient echo
GRASE: Gradient and spin echo
GRE: Gradient echo
HARP: Harmonic phase analysis
HD: Hausdorff distance
HCM: Hypertrophic cardiomyopathy
IVS: Interventricular septum
JI: Jaccard index
LA: Left atrial/left atrial
LL: Lower limit (of normal)
LV: Left ventricle/left ventricular
LVEF: Left ventricular ejection fraction
LVM: Left ventricular mass
LVOT: Left ventricular outflow tract
LVSC: Left Ventricle Segmentation Challenge
LMS: Lambda Mu Sigma
LPA: Left pulmonary artery
MBF: Myocardial blood flow
Mean_p_: Pooled weighted mean
MESA: Multi-Ethnic Study of Atherosclerosis
MICCAI: Medical Image Computing and Computer Assisted Intervention
MOLLI: Modified Look-Locker inversion recovery
MPA: Main pulmonary artery
MRI: Magnetic resonance imaging
MRA: MR angiography
NC/C: Non-compacted/compacted (left ventricular myocardium)
PFR: Peak filling rate
PC: Phase contrast
RA: Right atrium/right atrial
RF: Radiofrequency
RPA: Right pulmonary artery
RPP: Rate-pressure product
RV: Right ventricle/right ventricular
RVSC: Right Ventricle Segmentation Challenge
SASHA: Saturation Recovery Single-sHot Acquisition
SENC: Strain-encoding
SD: Standard deviation
SD_p_: Pooled weighted standard deviation
ShMOLLI: Shortened modified Look-Locker inversion recovery
SinMod: Sine wave modeling
SPAMM: Spatial modulation of magnetization
STACOM: Statistical Atlases and Computational Modelling of the Heart
SV: Stroke volume
T: Tesla
T1: T1-relaxation time
T2: T2-relaxation time
TM: Total (left ventricular myocardial) mass
TSE: Turbo-spin echo
TTE: Transthoracic echocardiography
UL: Upper limit (of normal)
V_enc_: Flow encoding velocity
VISTA: Volume isotropic surbo spin echo
yrs: Years

## References

[1] Kawel-Boehm N, Maceira A, Valsangiacomo-Buechel ER, Vogel-Claussen J, Turkbey EB, Williams R, Plein S, Tee M, Eng J, Bluemke DA (2015). Normal values for cardiovascular magnetic resonance in adults and children. J Cardiovasc Magn Reson.

[2] Bai W, Sinclair M, Tarroni G, Oktay O, Rajchl M, Vaillant G, Lee AM, Aung N, Lukaschuk E, Sanghvi MM (2018). Automated cardiovascular magnetic resonance image analysis with fully convolutional networks. J Cardiovasc Magn Reson.

[3] Tao Q, Yan W, Wang Y, Paiman EHM, Shamonin DP, Garg P, Plein S, Huang L, Xia L, Sramko M (2019). Deep Learning-based Method for Fully Automatic Quantification of Left Ventricle Function from Cine MR Images: A Multivendor Multicenter Study. Radiology.

[4] Horowitz GL (2010). Estimating reference intervals. Am J Clin Pathol.

[5] SCMR: Consensus/Position statements, [https://scmr.org/general/custom.asp?page=guidelines], Accessed 15 Dec 2019

[6] Chuang ML, Gona P, Hautvast GL, Salton CJ, Blease SJ, Yeon SB, Breeuwer M, O'Donnell CJ, Manning WJ (2012). Correlation of trabeculae and papillary muscles with clinical and cardiac characteristics and impact on CMR measures of LV anatomy and function. JACC Cardiovasc Imaging.

[7] Riffel JH, Schmucker K, Andre F, Ochs M, Hirschberg K, Schaub E, Fritz T, Mueller-Hennessen M, Giannitsis E, Katus HA, Friedrich MG (2019). Cardiovascular magnetic resonance of cardiac morphology and function: impact of different strategies of contour drawing and indexing. Clin Res Cardiol.

[8] Vogel-Claussen J, Finn JP, Gomes AS, Hundley GW, Jerosch-Herold M, Pearson G, Sinha S, Lima JA, Bluemke DA (2006). Left ventricular papillary muscle mass: relationship to left ventricular mass and volumes by magnetic resonance imaging. J Comput Assist Tomogr.

[9] Schulz-Menger J, Bluemke DA, Bremerich J, Flamm SD, Fogel MA, Friedrich MG, Kim RJ, von Knobelsdorff-Brenkenhoff F, Kramer CM, Pennell DJ (2020). Standardized image interpretation and post-processing in cardiovascular magnetic resonance–2020 update: Society for Cardiovascular Magnetic Resonance (SCMR): Board of Trustees Task Force on Standardized Post-Processing. J Cardiovasc Magn Reson.

[10] Maceira AM, Prasad SK, Khan M, Pennell DJ (2006). Normalized left ventricular systolic and diastolic function by steady state free precession cardiovascular magnetic resonance. J Cardiovasc Magn Reson.

[11] Le TT, Tan RS, De Deyn M, Goh EP, Han Y, Leong BR, Cook SA, Chin CW (2016). Cardiovascular magnetic resonance reference ranges for the heart and aorta in Chinese at 3T. J Cardiovasc Magn Reson.

[12] Bentatou Z, Finas M, Habert P, Kober F, Guye M, Bricq S, Lalande A, Frandon J, Dacher JN, Dubourg B (2018). Distribution of left ventricular trabeculation across age and gender in 140 healthy Caucasian subjects on MR imaging. Diagn Interv Imaging.

[13] Bulow R, Ittermann T, Dorr M, Poesch A, Langner S, Volzke H, Hosten N, Dewey M (2018). Reference ranges of left ventricular structure and function assessed by contrast-enhanced cardiac MR and changes related to ageing and hypertension in a population-based study. Eur Radiol.

[14] Le Ven F, Bibeau K, De Larochelliere E, Tizon-Marcos H, Deneault-Bissonnette S, Pibarot P, Deschepper CF, Larose E (2016). Cardiac morphology and function reference values derived from a large subset of healthy young Caucasian adults by magnetic resonance imaging. Eur Heart J Cardiovasc Imaging.

[15] Lei X, Liu H, Han Y, Cheng W, Sun J, Luo Y, Yang D, Dong Y, Chung Y, Chen Y (2017). Reference values of cardiac ventricular structure and function by steady-state free-procession MRI at 3.0T in healthy adult chinese volunteers. J Magn Reson Imaging.

[16] Petersen SE, Aung N, Sanghvi MM, Zemrak F, Fung K, Paiva JM, Francis JM, Khanji MY, Lukaschuk E, Lee AM (2017). Reference ranges for cardiac structure and function using cardiovascular magnetic resonance (CMR) in Caucasians from the UK Biobank population cohort. J Cardiovasc Magn Reson.

[17] Reiter G, Reiter U, Rienmuller R, Gagarina N, Ryabikin A (2004). On the value of geometry-based models for left ventricular volumetry in magnetic resonance imaging and electron beam tomography: a Bland-Altman analysis. Eur J Radiol.

[18] Aquaro GD, Camastra G, Monti L, Lombardi M, Pepe A, Castelletti S, Maestrini V, Todiere G, Masci P, di Giovine G (2017). Reference values of cardiac volumes, dimensions, and new functional parameters by MR: A multicenter, multivendor study. J Magn Reson Imaging.

[19] Gandy SJ, Lambert M, Belch J, Cavin I, Crowe E, Littleford R, MacFarlane JA, Matthew SZ, Martin P, Nicholas RS (2016). 3T MRI investigation of cardiac left ventricular structure and function in a UK population: The tayside screening for the prevention of cardiac events (TASCFORCE) study. J Magn Reson Imaging.

[20] Maroules CD, McColl R, Khera A, Peshock RM (2008). Interstudy reproducibility of SSFP cine magnetic resonance: impact of magnetic field strength and parallel imaging. J Magn Reson Imaging.

[21] Natori S, Lai S, Finn JP, Gomes AS, Hundley WG, Jerosch-Herold M, Pearson G, Sinha S, Arai A, Lima JA, Bluemke DA (2006). Cardiovascular function in multi-ethnic study of atherosclerosis: normal values by age, sex, and ethnicity. AJR Am J Roentgenol.

[22] Hudsmith LE, Petersen SE, Francis JM, Robson MD, Neubauer S (2005). Normal human left and right ventricular and left atrial dimensions using steady state free precession magnetic resonance imaging. J Cardiovasc Magn Reson.

[23] Chang SA, Choe YH, Jang SY, Kim SM, Lee SC, Oh JK (2012). Assessment of left and right ventricular parameters in healthy Korean volunteers using cardiac magnetic resonance imaging: change in ventricular volume and function based on age, gender and body surface area. Int J Cardiovasc Imaging.

[24] Macedo R, Fernandes JL, Andrade SS, Rochitte CE, Lima KC, Maciel AC, Maciel FC, Alves GS, Coelho OR, Diniz RV (2013). Morphological and functional measurements of the heart obtained by magnetic resonance imaging in Brazilians. Arq Bras Cardiol.

[25] Yeon SB, Salton CJ, Gona P, Chuang ML, Blease SJ, Han Y, Tsao CW, Danias PG, Levy D, O'Donnell CJ, Manning WJ (2015). Impact of age, sex, and indexation method on MR left ventricular reference values in the Framingham Heart Study offspring cohort. J Magn Reson Imaging.

[26] Liu B, Dardeer AM, Moody WE, Hayer MK, Baig S, Price AM, Leyva F, Edwards NC, Steeds RP (2018). Reference ranges for three-dimensional feature tracking cardiac magnetic resonance: comparison with two-dimensional methodology and relevance of age and gender. Int J Cardiovasc Imaging.

[27] Sievers B, Kirchberg S, Bakan A, Franken U, Trappe HJ (2004). Impact of papillary muscles in ventricular volume and ejection fraction assessment by cardiovascular magnetic resonance. J Cardiovasc Magn Reson.

[28] Winter MM, Bernink FJ, Groenink M, Bouma BJ, van Dijk AP, Helbing WA, Tijssen JG, Mulder BJ (2008). Evaluating the systemic right ventricle by CMR: the importance of consistent and reproducible delineation of the cavity. J Cardiovasc Magn Reson.

[29] Maceira AM, Prasad SK, Khan M, Pennell DJ (2006). Reference right ventricular systolic and diastolic function normalized to age, gender and body surface area from steady-state free precession cardiovascular magnetic resonance. Eur Heart J.

[30] Nacif MS, Barranhas AD, Turkbey E, Marchiori E, Kawel N, Mello RA, Falcao RO, Oliveira AC, Rochitte CE (2013). Left atrial volume quantification using cardiac MRI in atrial fibrillation: comparison of the Simpson's method with biplane area-length, ellipse, and three-dimensional methods. Diagn Interv Radiol.

[31] Maceira AM, Cosin-Sales J, Roughton M, Prasad SK, Pennell DJ (2010). Reference left atrial dimensions and volumes by steady state free precession cardiovascular magnetic resonance. J Cardiovasc Magn Reson.

[32] Funk S, Kermer J, Doganguezel S, Schwenke C, von Knobelsdorff-Brenkenhoff F, Schulz-Menger J (2018). Quantification of the left atrium applying cardiovascular magnetic resonance in clinical routine. Scand Cardiovasc J.

[33] Li W, Wan K, Han Y, Liu H, Cheng W, Sun J, Luo Y, Yang D, Chung YC, Chen Y (2017). Reference value of left and right atrial size and phasic function by SSFP CMR at 3.0 T in healthy Chinese adults. Sci Rep.

[34] Zemrak F, Ambale-Venkatesh B, Captur G, Chrispin J, Chamera E, Habibi M, Nazarian S, Mohiddin SA, Moon JC, Petersen SE (2017). Left Atrial Structure in Relationship to Age, Sex, Ethnicity, and Cardiovascular Risk Factors: MESA (Multi-Ethnic Study of Atherosclerosis). Circ Cardiovasc Imaging.

[35] Sievers B, Kirchberg S, Franken U, Bakan A, Addo M, John-Puthenveettil B, Trappe HJ (2005). Determination of normal gender-specific left atrial dimensions by cardiovascular magnetic resonance imaging. J Cardiovasc Magn Reson.

[36] Rohner A, Brinkert M, Kawel N, Buechel RR, Leibundgut G, Grize L, Kuhne M, Bremerich J, Kaufmann BA, Zellweger MJ (2011). Functional assessment of the left atrium by real-time three-dimensional echocardiography using a novel dedicated analysis tool: initial validation studies in comparison with computed tomography. Eur J Echocardiogr.

[37] Maceira AM, Cosin-Sales J, Roughton M, Prasad SK, Pennell DJ (2013). Reference right atrial dimensions and volume estimation by steady state free precession cardiovascular magnetic resonance. J Cardiovasc Magn Reson.

[38] Maceira AM, Cosin-Sales J, Prasad SK, Pennell DJ (2016). Characterization of left and right atrial function in healthy volunteers by cardiovascular magnetic resonance. J Cardiovasc Magn Reson.

[39] Buechel EV, Kaiser T, Jackson C, Schmitz A, Kellenberger CJ (2009). Normal right- and left ventricular volumes and myocardial mass in children measured by steady state free precession cardiovascular magnetic resonance. J Cardiovasc Magn Reson.

[40] Robbers-Visser D, Boersma E, Helbing WA (2009). Normal biventricular function, volumes, and mass in children aged 8 to 17 years. J Magn Reson Imaging.

[41] Sarikouch S, Peters B, Gutberlet M, Leismann B, Kelter-Kloepping A, Koerperich H, Kuehne T, Beerbaum P (2010). Sex-specific pediatric percentiles for ventricular size and mass as reference values for cardiac MRI: assessment by steady-state free-precession and phase-contrast MRI flow. Circ Cardiovasc Imaging.

[42] Dewey FE, Rosenthal D, Murphy DJ, Froelicher VF, Ashley EA (2008). Does size matter? Clinical applications of scaling cardiac size and function for body size. Circulation.

[43] Sluysmans T, Colan SD (1985). Theoretical and empirical derivation of cardiovascular allometric relationships in children. J Appl Physiol.

[44] Cole TJ (1990). The LMS method for constructing normalized growth standards. Eur J Clin Nutr.

[45] van der Ven JPG, Sadighy Z, Valsangiacomo Buechel ER, Sarikouch S, Robbers-Visser D, Kellenberger CJ, Kaiser T, Beerbaum P, Boersma E, Helbing WA (2019). Multicentre reference values for cardiac magnetic resonance imaging derived ventricular size and function for children aged 0–18 years. Eur Heart J Cardiovasc Imaging.

[46] Schulz-Menger J, Bluemke DA, Bremerich J, Flamm SD, Fogel MA, Friedrich MG, Kim RJ, von Knobelsdorff-Brenkenhoff F, Kramer CM, Pennell DJ (2013). Standardized image interpretation and post processing in cardiovascular magnetic resonance: Society for Cardiovascular Magnetic Resonance (SCMR) board of trustees task force on standardized post processing. J Cardiovasc Magn Reson.

[47] Sarikouch S, Koerperich H, Boethig D, Peters B, Lotz J, Gutberlet M, Beerbaum P, Kuehne T (2011). Reference values for atrial size and function in children and young adults by cardiac MR: a study of the German competence network congenital heart defects. J Magn Reson Imaging.

[48] Mitchell JH, Haskell W, Snell P, Van Camp SP (2005). Task Force 8: classification of sports. J Am Coll Cardiol.

[49] D'Ascenzi F, Anselmi F, Piu P, Fiorentini C, Carbone SF, Volterrani L, Focardi M, Bonifazi M, Mondillo S (2019). Cardiac Magnetic Resonance Normal Reference Values of Biventricular Size and Function in Male Athlete's Heart. JACC Cardiovasc Imaging.

[50] Prakken NH, Velthuis BK, Teske AJ, Mosterd A, Mali WP, Cramer MJ (2010). Cardiac MRI reference values for athletes and nonathletes corrected for body surface area, training hours/week and sex. Eur J Cardiovasc Prev Rehabil.

[51] Luijkx T, Velthuis BK, Prakken NH, Cox MG, Bots ML, Mali WP, Hauer RN, Cramer MJ (2012). Impact of revised Task Force Criteria: distinguishing the athlete's heart from ARVC/D using cardiac magnetic resonance imaging. Eur J Prev Cardiol.

[52] Tahir E, Starekova J, Muellerleile K, von Stritzky A, Munch J, Avanesov M, Weinrich JM, Stehning C, Bohnen S, Radunski UK (2018). Myocardial Fibrosis in Competitive Triathletes Detected by Contrast-Enhanced CMR Correlates With Exercise-Induced Hypertension and Competition History. JACC Cardiovasc Imaging.

[53] Alfakih K, Plein S, Thiele H, Jones T, Ridgway JP, Sivananthan MU (2003). Normal human left and right ventricular dimensions for MRI as assessed by turbo gradient echo and steady-state free precession imaging sequences. J Magn Reson Imaging.

[54] Malayeri AA, Johnson WC, Macedo R, Bathon J, Lima JA, Bluemke DA (2008). Cardiac cine MRI: Quantification of the relationship between fast gradient echo and steady-state free precession for determination of myocardial mass and volumes. J Magn Reson Imaging.

[55] Kawel N, Turkbey EB, Carr JJ, Eng J, Gomes AS, Hundley WG, Johnson C, Masri SC, Prince MR, van der Geest RJ (2012). Normal left ventricular myocardial thickness for middle-aged and older subjects with steady-state free precession cardiac magnetic resonance: the multi-ethnic study of atherosclerosis. Circ Cardiovasc Imaging.

[56] Dawson DK, Maceira AM, Raj VJ, Graham C, Pennell DJ, Kilner PJ (2011). Regional thicknesses and thickening of compacted and trabeculated myocardial layers of the normal left ventricle studied by cardiovascular magnetic resonance. Circ Cardiovasc Imaging.

[57] Captur G, Karperien AL, Li C, Zemrak F, Tobon-Gomez C, Gao X, Bluemke DA, Elliott PM, Petersen SE, Moon JC (2015). Fractal frontiers in cardiovascular magnetic resonance: towards clinical implementation. J Cardiovasc Magn Reson.

[58] Amzulescu MS, Rousseau MF, Ahn SA, Boileau L (2015). Prognostic impact of hypertrabeculation and noncompaction phenotype in dilated cardiomyopathy: a CMR study. JACC Cardiovasc Imaging.

[59] Captur G, Muthurangu V, Cook C, Flett AS, Wilson R, Barison A, Sado DM, Anderson S, McKenna WJ, Mohun TJ (2013). Quantification of left ventricular trabeculae using fractal analysis. J Cardiovasc Magn Reson.

[60] Captur G, Lopes LR, Patel V, Li C, Bassett P, Syrris P, Sado DM, Maestrini V, Mohun TJ, McKenna WJ (2014). Abnormal cardiac formation in hypertrophic cardiomyopathy: fractal analysis of trabeculae and preclinical gene expression. Circ Cardiovasc Genet.

[61] Kawel N, Nacif M, Arai AE, Gomes AS, Hundley WG, Johnson WC, Prince MR, Stacey RB, Lima JA, Bluemke DA (2012). Trabeculated (noncompacted) and compact myocardium in adults: the multi-ethnic study of atherosclerosis. Circ Cardiovasc Imaging.

[62] Captur G, Zemrak F, Muthurangu V, Petersen SE, Li C, Bassett P, Kawel-Boehm N, McKenna WJ, Elliott PM, Lima JA (2015). Fractal analysis of myocardial trabeculations in 2547 study participants: multi-ethnic study of atherosclerosis. Radiology.

[63] Luijkx T, Cramer MJ, Zaidi A, Rienks R, Senden PJ, Sharma S, van Hellemondt FJ, Buckens CF, Mali WP, Velthuis BK (2012). Ethnic differences in ventricular hypertrabeculation on cardiac MRI in elite football players. Neth Heart J.

[64] Andre F, Burger A, Lossnitzer D, Buss SJ, Abdel-Aty H, Gianntisis E, Steen H, Katus HA (2015). Reference values for left and right ventricular trabeculation and non-compacted myocardium. Int J Cardiol.

[65] Tizon-Marcos H, de la Paz RM, Pibarot P, Bertrand O, Bibeau K, Le Ven F, Sinha S, Engert J, Bedard E, Pasian S (2014). Characteristics of trabeculated myocardium burden in young and apparently healthy adults. Am J Cardiol.

[66] Cai J, Bryant JA, Le TT, Su B, de Marvao A, O'Regan DP, Cook SA, Chin CW (2017). Fractal analysis of left ventricular trabeculations is associated with impaired myocardial deformation in healthy Chinese. J Cardiovasc Magn Reson.

[67] Sondergaard L, Stahlberg F, Thomsen C, Spraggins TA, Gymoese E, Malmgren L, Muller E, Henriksen O (1993). Comparison between retrospective gating and ECG triggering in magnetic resonance velocity mapping. Magn Reson Imaging.

[68] Allen BD, Barker AJ, Carr JC, Silverberg RA, Markl M (2013). Time-resolved three-dimensional phase contrast MRI evaluation of bicuspid aortic valve and coarctation of the aorta. Eur Heart J Cardiovasc Imaging.

[69] Kupfahl C, Honold M, Meinhardt G, Vogelsberg H, Wagner A, Mahrholdt H, Sechtem U (2004). Evaluation of aortic stenosis by cardiovascular magnetic resonance imaging: comparison with established routine clinical techniques. Heart.

[70] Lotz J, Meier C, Leppert A, Galanski M (2002). Cardiovascular flow measurement with phase-contrast MR imaging: basic facts and implementation. Radiographics.

[71] Srichai MB, Lim RP, Wong S, Lee VS (2009). Cardiovascular applications of phase-contrast MRI. AJR Am J Roentgenol.

[72] Caruthers SD, Lin SJ, Brown P, Watkins MP, Williams TA, Lehr KA, Wickline SA (2003). Practical value of cardiac magnetic resonance imaging for clinical quantification of aortic valve stenosis: comparison with echocardiography. Circulation.

[73] Kilner PJ, Manzara CC, Mohiaddin RH, Pennell DJ, Sutton MG, Firmin DN, Underwood SR, Longmore DB (1993). Magnetic resonance jet velocity mapping in mitral and aortic valve stenosis. Circulation.

[74] Myerson SG (2012). Heart valve disease: investigation by cardiovascular magnetic resonance. J Cardiovasc Magn Reson.

[75] Rathi VK, Doyle M, Yamrozik J, Williams RB, Caruppannan K, Truman C, Vido D, Biederman RW (2008). Routine evaluation of left ventricular diastolic function by cardiovascular magnetic resonance: a practical approach. J Cardiovasc Magn Reson.

[76] Callaghan FM, Bannon P, Barin E, Celemajer D, Jeremy R, Figtree G, Grieve SM (2019). Age-related changes of shape and flow dynamics in healthy adult aortas: a 4D flow MRI study. J Magn Reson Imaging.

[77] Garcia J, van der Palen RLF, Bollache E, Jarvis K, Rose MJ, Barker AJ, Collins JD, Carr JC, Robinson J, Rigsby CK, Markl M (2018). Distribution of blood flow velocity in the normal aorta: effect of age and gender. J Magn Reson Imaging.

[78] Nishimura RA, Otto CM, Bonow RO, Carabello BA, Erwin JP, Fleisher LA, Jneid H, Mack MJ, McLeod CJ, O'Gara PT (2017). 2017 AHA/ACC Focused Update of the 2014 AHA/ACC Guideline for the Management of Patients With Valvular Heart Disease: A Report of the American College of Cardiology/American Heart Association Task Force on Clinical Practice Guidelines. J Am Coll Cardiol.

[79] Nishimura RA, Otto CM, Bonow RO, Carabello BA, Erwin JP, Guyton RA, O'Gara PT, Ruiz CE, Skubas NJ, Sorajja P (2014). 2014 AHA/ACC guideline for the management of patients with valvular heart disease: executive summary: a report of the American College of Cardiology/American Heart Association Task Force on Practice Guidelines. J Am Coll Cardiol.

[80] Caudron J, Fares J, Bauer F, Dacher JN (2011). Evaluation of left ventricular diastolic function with cardiac MR imaging. Radiographics.

[81] Kawel N, Jhooti P, Dashti D, Haas T, Winter L, Zellweger MJ, Buser PT, Keegan J, Scheffler K, Bremerich J (2012). MR-imaging of the thoracic aorta: 3D-ECG- and respiratory-gated bSSFP imaging using the CLAWS algorithm versus contrast-enhanced 3D-MRA. Eur J Radiol.

[82] Potthast S, Mitsumori L, Stanescu LA, Richardson ML, Branch K, Dubinsky TJ, Maki JH (2010). Measuring aortic diameter with different MR techniques: comparison of three-dimensional (3D) navigated steady-state free-precession (SSFP), 3D contrast-enhanced magnetic resonance angiography (CE-MRA), 2D T2 black blood, and 2D cine SSFP. J Magn Reson Imaging.

[83] Turkbey EB, Jain A, Johnson C, Redheuil A, Arai AE, Gomes AS, Carr J, Hundley WG, Teixido-Tura G, Eng J (2014). Determinants and normal values of ascending aortic diameter by age, gender, and race/ethnicity in the Multi-Ethnic Study of Atherosclerosis (MESA). J Magn Reson Imaging.

[84] Eikendal AL, Bots ML, Haaring C, Saam T, van der Geest RJ, Westenberg JJ, den Ruijter HM, Hoefer IE, Leiner T (2016). Reference values for cardiac and aortic magnetic resonance imaging in healthy young caucasian adults. PLoS ONE.

[85] Burman ED, Keegan J, Kilner PJ (2008). Aortic root measurement by cardiovascular magnetic resonance: specification of planes and lines of measurement and corresponding normal values. Circ Cardiovasc Imaging.

[86] Davis AE, Lewandowski AJ, Holloway CJ, Ntusi NA, Banerjee R, Nethononda R, Pitcher A, Francis JM, Myerson SG, Leeson P (2014). Observational study of regional aortic size referenced to body size: production of a cardiovascular magnetic resonance nomogram. J Cardiovasc Magn Reson.

[87] Lederle FA, Johnson GR, Wilson SE, Chute EP, Littooy FN, Bandyk D, Krupski WC, Barone GW, Acher CW, Ballard DJ (1997). Prevalence and associations of abdominal aortic aneurysm detected through screening. Aneurysm Detection and Management (ADAM) Veterans Affairs Cooperative Study Group. Ann Intern Med.

[88] Redheuil A, Yu WC, Wu CO, Mousseaux E, de Cesare A, Yan R, Kachenoura N, Bluemke D, Lima JA (2010). Reduced ascending aortic strain and distensibility: earliest manifestations of vascular aging in humans. Hypertension.

[89] Redheuil A, Yu WC, Mousseaux E, Harouni AA, Kachenoura N, Wu CO, Bluemke D, Lima JA (2011). Age-related changes in aortic arch geometry: relationship with proximal aortic function and left ventricular mass and remodeling. J Am Coll Cardiol.

[90] Sugawara J, Hayashi K, Yokoi T, Tanaka H (2008). Age-associated elongation of the ascending aorta in adults. JACC Cardiovasc Imaging.

[91] Kaiser T, Kellenberger CJ, Albisetti M, Bergstrasser E, Valsangiacomo Buechel ER (2008). Normal values for aortic diameters in children and adolescents–assessment in vivo by contrast-enhanced CMR-angiography. J Cardiovasc Magn Reson.

[92] Voges I, Jerosch-Herold M, Hedderich J, Pardun E, Hart C, Gabbert DD, Hansen JH, Petko C, Kramer HH, Rickers C (2012). Normal values of aortic dimensions, distensibility, and pulse wave velocity in children and young adults: a cross-sectional study. J Cardiovasc Magn Reson.

[93] Kutty S, Kuehne T, Gribben P, Reed E, Li L, Danford DA, Beerbaum PB, Sarikouch S (2012). Ascending aortic and main pulmonary artery areas derived from cardiovascular magnetic resonance as reference values for normal subjects and repaired tetralogy of Fallot. Circ Cardiovasc Imaging.

[94] Dogui A, Redheuil A, Lefort M, DeCesare A, Kachenoura N, Herment A, Mousseaux E (2011). Measurement of aortic arch pulse wave velocity in cardiovascular MR: comparison of transit time estimators and description of a new approach. J Magn Reson Imaging.

[95] Turkbey EB, Redheuil A, Backlund JY, Small AC, Cleary PA, Lachin JM, Lima JA, Bluemke DA, Diabetes C (2013). Complications Trial/Epidemiology of Diabetes I, Complications Research G: Aortic distensibility in type 1 diabetes. Diabetes Care.

[96] Cavalcante JL, Lima JA, Redheuil A, Al-Mallah MH (2011). Aortic stiffness: current understanding and future directions. J Am Coll Cardiol.

[97] Rose JL, Lalande A, Bouchot O, el Bourennane B, Walker PM, Ugolini P, Revol-Muller C, Cartier R, Brunotte F (2010). Influence of age and sex on aortic distensibility assessed by MRI in healthy subjects. Magn Reson Imaging.

[98] Kim EK, Chang SA, Jang SY, Kim Y, Kim SM, Oh JK, Choe YH, Kim DK (2013). Assessment of regional aortic stiffness with cardiac magnetic resonance imaging in a healthy Asian population. Int J Cardiovasc Imaging.

[99] Burman ED, Keegan J, Kilner PJ (2016). Pulmonary artery diameters, cross sectional areas and area changes measured by cine cardiovascular magnetic resonance in healthy volunteers. J Cardiovasc Magn Reson.

[100] Knobel Z, Kellenberger CJ, Kaiser T, Albisetti M, Bergstrasser E, Buechel ER (2011). Geometry and dimensions of the pulmonary artery bifurcation in children and adolescents: assessment in vivo by contrast-enhanced MR-angiography. Int J Cardiovasc Imaging.

[101] Gottbrecht M, Kramer CM, Salerno M (2019). Native T1 and extracellular volume measurements by cardiac MRI in healthy adults: a meta-analysis. Radiology.

[102] Messroghli DR, Moon JC, Ferreira VM, Grosse-Wortmann L, He T, Kellman P, Mascherbauer J, Nezafat R, Salerno M, Schelbert EB (2017). Clinical recommendations for cardiovascular magnetic resonance mapping of T1, T2, T2* and extracellular volume: A consensus statement by the Society for Cardiovascular Magnetic Resonance (SCMR) endorsed by the European Association for Cardiovascular Imaging (EACVI). J Cardiovasc Magn Reson.

[103] Messroghli DR, Radjenovic A, Kozerke S, Higgins DM, Sivananthan MU, Ridgway JP (2004). Modified Look-Locker inversion recovery (MOLLI) for high-resolution T1 mapping of the heart. Magn Reson Med.

[104] Piechnik SK, Ferreira VM, Dall'Armellina E, Cochlin LE, Greiser A, Neubauer S, Robson MD (2010). Shortened Modified Look-Locker Inversion recovery (ShMOLLI) for clinical myocardial T1-mapping at 1.5 and 3 T within a 9 heartbeat breathhold. J Cardiovasc Magn Reson.

[105] Chow K, Flewitt JA, Green JD, Pagano JJ, Friedrich MG, Thompson RB (2014). Saturation recovery single-shot acquisition (SASHA) for myocardial T(1) mapping. Magn Reson Med.

[106] Schelbert EB, Testa SM, Meier CG, Ceyrolles WJ, Levenson JE, Blair AJ, Kellman P, Jones BL, Ludwig DR, Schwartzman D (2011). Myocardial extravascular extracellular volume fraction measurement by gadolinium cardiovascular magnetic resonance in humans: slow infusion versus bolus. J Cardiovasc Magn Reson.

[107] Kawel N, Nacif M, Zavodni A, Jones J, Liu S, Sibley CT, Bluemke DA (2012). T1 mapping of the myocardium: intra-individual assessment of the effect of field strength, cardiac cycle and variation by myocardial region. J Cardiovasc Magn Reson.

[108] Gai N, Turkbey EB, Nazarian S, van der Geest RJ, Liu CY, Lima JA, Bluemke DA (2011). T1 mapping of the gadolinium-enhanced myocardium: adjustment for factors affecting interpatient comparison. Magn Reson Med.

[109] Kawel N, Nacif M, Zavodni A, Jones J, Liu S, Sibley CT, Bluemke DA (2012). T1 mapping of the myocardium: intra-individual assessment of post-contrast T1 time evolution and extracellular volume fraction at 3T for Gd-DTPA and Gd-BOPTA. J Cardiovasc Magn Reson.

[110] Lee JJ, Liu S, Nacif MS, Ugander M, Han J, Kawel N, Sibley CT, Kellman P, Arai AE, Bluemke DA (2011). Myocardial T1 and extracellular volume fraction mapping at 3 tesla. J Cardiovasc Magn Reson.

[111] Puntmann VO, D'Cruz D, Smith Z, Pastor A, Choong P, Voigt T, Carr-White G, Sangle S, Schaeffter T, Nagel E (2013). Native myocardial T1 mapping by cardiovascular magnetic resonance imaging in subclinical cardiomyopathy in patients with systemic lupus erythematosus. Circ Cardiovasc Imaging.

[112] Ugander M, Oki AJ, Hsu LY, Kellman P, Greiser A, Aletras AH, Sibley CT, Chen MY, Bandettini WP, Arai AE (2012). Extracellular volume imaging by magnetic resonance imaging provides insights into overt and sub-clinical myocardial pathology. Eur Heart J.

[113] White SK, Sado DM, Flett AS, Moon JC (2012). Characterising the myocardial interstitial space: the clinical relevance of non-invasive imaging. Heart.

[114] Arheden H, Saeed M, Higgins CB, Gao DW, Ursell PC, Bremerich J, Wyttenbach R, Dae MW, Wendland MF (2000). Reperfused rat myocardium subjected to various durations of ischemia: estimation of the distribution volume of contrast material with echo-planar MR imaging. Radiology.

[115] Kellman P, Wilson JR, Xue H, Ugander M, Arai AE (2012). Extracellular volume fraction mapping in the myocardium, part 1: evaluation of an automated method. J Cardiovasc Magn Reson.

[116] Piechnik SK, Ferreira VM, Lewandowski AJ, Ntusi NA, Banerjee R, Holloway C, Hofman MB, Sado DM, Maestrini V, White SK (2013). Normal variation of magnetic resonance T1 relaxation times in the human population at 1.5 T using ShMOLLI. J Cardiovasc Magn Reson.

[117] Dabir D, Child N, Kalra A, Rogers T, Gebker R, Jabbour A, Plein S, Yu CY, Otton J, Kidambi A (2014). Reference values for healthy human myocardium using a T1 mapping methodology: results from the International T1 Multicenter cardiovascular magnetic resonance study. J Cardiovasc Magn Reson.

[118] Reiter U, Reiter G, Dorr K, Greiser A, Maderthaner R, Fuchsjager M (2014). Normal diastolic and systolic myocardial T1 values at 1.5-T MR imaging: correlations and blood normalization. Radiology.

[119] Kawel N, Nacif M, Santini F, Liu S, Bremerich J, Arai AE, Bluemke DA (2012). Partition coefficients for gadolinium chelates in the normal myocardium: comparison of gadopentetate dimeglumine and gadobenate dimeglumine. J Magn Reson Imaging.

[120] Fontana M, White SK, Banypersad SM, Sado DM, Maestrini V, Flett AS, Piechnik SK, Neubauer S, Roberts N, Moon JC (2012). Comparison of T1 mapping techniques for ECV quantification. Histological validation and reproducibility of ShMOLLI versus multibreath-hold T1 quantification equilibrium contrast CMR. J Cardiovasc Magn Reson.

[121] Kellman P, Wilson JR, Xue H, Bandettini WP, Shanbhag SM, Druey KM, Ugander M, Arai AE (2012). Extracellular volume fraction mapping in the myocardium, part 2: initial clinical experience. J Cardiovasc Magn Reson.

[122] Sado DM, White SK, Piechnik SK, Banypersad SM, Treibel T, Captur G, Fontana M, Maestrini V, Flett AS, Robson MD (2013). Identification and assessment of Anderson-Fabry disease by cardiovascular magnetic resonance noncontrast myocardial T1 mapping. Circ Cardiovasc Imaging.

[123] Ferreira VM, Piechnik SK, Dall'Armellina E, Karamitsos TD, Francis JM, Ntusi N, Holloway C, Choudhury RP, Kardos A, Robson MD (2014). Native T1-mapping detects the location, extent and patterns of acute myocarditis without the need for gadolinium contrast agents. J Cardiovasc Magn Reson.

[124] Fontana M, Banypersad SM, Treibel TA, Maestrini V, Sado DM, White SK, Pica S, Castelletti S, Piechnik SK, Robson MD (2014). Native T1 mapping in transthyretin amyloidosis. JACC Cardiovasc Imaging.

[125] Liu CY, Bluemke DA, Gerstenblith G, Zimmerman SL, Li J, Zhu H, Lai S, Lai H (2014). Reference values of myocardial structure, function, and tissue composition by cardiac magnetic resonance in healthy African-Americans at 3T and their relations to serologic and cardiovascular risk factors. Am J Cardiol.

[126] Puntmann VO, Arroyo Ucar E, Hinojar Baydes R, Ngah NB, Kuo YS, Dabir D, Macmillan A, Cummins C, Higgins DM, Gaddum N (2014). Aortic stiffness and interstitial myocardial fibrosis by native T1 are independently associated with left ventricular remodeling in patients with dilated cardiomyopathy. Hypertension.

[127] Siepen F, Buss SJ, Messroghli D, Andre F, Lossnitzer D, Seitz S, Keller M, Schnabel PA, Giannitsis E, Korosoglou G (2015). T1 mapping in dilated cardiomyopathy with cardiac magnetic resonance: quantification of diffuse myocardial fibrosis and comparison with endomyocardial biopsy. Eur Heart J Cardiovasc Imaging.

[128] Banypersad SM, Fontana M, Maestrini V, Sado DM, Captur G, Petrie A, Piechnik SK, Whelan CJ, Herrey AS, Gillmore JD (2015). T1 mapping and survival in systemic light-chain amloidosis. Eur Heart J.

[129] Edwards NC, Moody WE, Yuan M, Hayer MK, Ferro CJ, Townend JN, Steeds RP (2015). Diffuse interstitial fibrosis and myocardial dysfunction in early chronic kidney disease. Am J Cardiol.

[130] Fontana M, Banypersad SM, Treibel TA, Abdel-Gadir A, Maestrini V, Lane T, Gilbertson JA, Hutt DF, Lachmann HJ, Whelan CJ (2015). Differential myocyte responses in patients with cardiac transthyretin amyloidosis and light-chain amyloidosis: a cardiac MR Imaging Study. Radiology.

[131] Treibel TA, Zemrak F, Sado DM, Banypersad SM, White SK, Maestrini V, Barison A, Patel V, Herrey AS, Davies C (2015). Extracellular volume quantification in isolated hypertension–changes at the detectable limits?. J Cardiovasc Magn Reson.

[132] Goebel J, Seifert I, Nensa F, Schemuth HP, Maderwald S, Quick HH, Schlosser T, Jensen C, Bruder O, Nassenstein K (2016). Can native t1 mapping differentiate between healthy and diffuse diseased myocardium in clinical routine cardiac MR imaging?. PLoS ONE.

[133] Gormeli CA, Gormeli G, Yagmur J, Ozdemir ZM, Kahraman AS, Colak C, Ozdemir R (2016). Assessment of myocardial changes in athletes with native T1 mapping and cardiac functional evaluation using 3 T MRI. Int J Cardiovasc Imaging.

[134] Hinojar R, Foote L, Sangle S, Marber M, Mayr M, Carr-White G, D'Cruz D, Nagel E, Puntmann VO (2016). Native T1 and T2 mapping by CMR in lupus myocarditis: Disease recognition and response to treatment. Int J Cardiol.

[135] Ntusi N, O'Dwyer E, Dorrell L, Wainwright E, Piechnik S, Clutton G, Hancock G, Ferreira V, Cox P, Badri M (2016). HIV-1-related cardiovascular disease is associated with chronic inflammation, frequent pericardial effusions, and probable myocardial edema. Circ Cardiovasc Imaging.

[136] Rauhalammi SM, Mangion K, Barrientos PH, Carrick DJ, Clerfond G, McClure J, McComb C, Radjenovic A, Berry C (2016). Native myocardial longitudinal (T1) relaxation time: Regional, age, and sex associations in the healthy adult heart. J Magn Reson Imaging.

[137] Costello BT, Springer F, Hare JL, Gerche A, Iles L, Ellims AH, Schmitt B, Taylor AJ (2017). SASHA versus ShMOLLI: a comparison of T1 mapping methods in health and dilated cardiomyopathy at 3 T. Int J Cardiovasc Imaging.

[138] Avitzur N, Satriano A, Afzal M, Narous M, Mikami Y, Hansen R, Dobko G, Flewitt J, Lydell CP, Howarth AG (2018). 3D myocardial deformation analysis from cine MRI as a marker of amyloid protein burden in cardiac amyloidosis: validation versus T1 mapping. Int J Cardiovasc Imaging.

[139] Doerner J, Eichhorn L, Luetkens JA, Lunkenheimer JN, Albers J, Nadal J, Schild HH, Naehle CP (2018). Effects of repetitive prolonged breath-hold in elite divers on myocardial fibrosis and cerebral morphology. Eur J Radiol.

[140] Guo Q, Wu LM, Wang Z, Shen JY, Su X, Wang CQ, Gong XR, Yan QR, He Q, Zhang W (2018). Early detection of silent myocardial impairment in drug-naive patients with new-onset systemic lupus erythematosus: a three-center prospective study. Arthritis Rheumatol.

[141] Ridouani F, Damy T, Tacher V, Derbel H, Legou F, Sifaoui I, Audureau E, Bodez D, Rahmouni A, Deux JF (2018). Myocardial native T2 measurement to differentiate light-chain and transthyretin cardiac amyloidosis and assess prognosis. J Cardiovasc Magn Reson.

[142] Rosmini S, Bulluck H, Captur G, Treibel TA, Abdel-Gadir A, Bhuva AN, Culotta V, Merghani A, Fontana M, Maestrini V (2018). Myocardial native T1 and extracellular volume with healthy ageing and gender. Eur Heart J Cardiovasc Imaging.

[143] Shang Y, Zhang X, Zhou X, Wang J (2018). Extracellular volume fraction measurements derived from the longitudinal relaxation of blood-based synthetic hematocrit may lead to clinical errors in 3 T cardiovascular magnetic resonance. J Cardiovasc Magn Reson.

[144] Yang D, Li X, Sun JY, Cheng W, Greiser A, Zhang TJ, Liu H, Wan K, Luo Y, An Q (2018). Cardiovascular magnetic resonance evidence of myocardial fibrosis and its clinical significance in adolescent and adult patients with Ebstein's anomaly. J Cardiovasc Magn Reson.

[145] Granitz M, Motloch LJ, Granitz C, Meissnitzer M, Hitzl W, Hergan K, Schlattau A (2019). Comparison of native myocardial T1 and T2 mapping at 1.5T and 3T in healthy volunteers: Reference values and clinical implications. Wien Klin Wochenschr.

[146] Imran M, Wang L, McCrohon J, Yu C, Holloway C, Otton J, Huang J, Stehning C, Moffat KJ, Ross J (2019). Native T1 mapping in the diagnosis of cardiac allograft rejection: a prospective histologically validated study. JACC Cardiovasc Imaging.

[147] Lehmonen L, Kaasalainen T, Atula S, Mustonen T, Holmstrom M (2019). Myocardial tissue characterization in patients with hereditary gelsolin (AGel) amyloidosis using novel cardiovascular magnetic resonance techniques. Int J Cardiovasc Imaging.

[148] Vijapurapu R, Nordin S, Baig S, Liu B, Rosmini S, Augusto J, Tchan M, Hughes DA, Geberhiwot T, Moon JC (2019). Global longitudinal strain, myocardial storage and hypertrophy in Fabry disease. Heart.

[149] Wan K, Li W, Sun J, Xu Y, Wang J, Liu H, Dong Y, Cheng W, Zhang Q, Zeng Z (2019). Regional amyloid distribution and impact on mortality in light-chain amyloidosis: a T1 mapping cardiac magnetic resonance study. Amyloid.

[150] Brittain JH, Hu BS, Wright GA, Meyer CH, Macovski A, Nishimura DG (1995). Coronary angiography with magnetization-prepared T2 contrast. Magn Reson Med.

[151] Giri S, Chung YC, Merchant A, Mihai G, Rajagopalan S, Raman SV, Simonetti OP (2009). T2 quantification for improved detection of myocardial edema. J Cardiovasc Magn Reson.

[152] Kamath R, Gottbrecht M, Salerno M. T2 relatxation times in healthy adults: a meta-analysis. Abstract submitted to SCMR 23rd Annual Scientific Session; 2019.

[153] Roy C, Slimani A, de Meester C, Amzulescu M, Pasquet A, Vancraeynest D, Vanoverschelde JL, Pouleur AC, Gerber BL (2017). Age and sex corrected normal reference values of T1, T2 T2* and ECV in healthy subjects at 3T CMR. J Cardiovasc Magn Reson.

[154] von Knobelsdorff-Brenkenhoff F, Prothmann M, Dieringer MA, Wassmuth R, Greiser A, Schwenke C, Niendorf T, Schulz-Menger J (2013). Myocardial T1 and T2 mapping at 3 T: reference values, influencing factors and implications. J Cardiovasc Magn Reson.

[155] Wassmuth R, Prothmann M, Utz W, Dieringer M, von Knobelsdorff-Brenkenhoff F, Greiser A, Schulz-Menger J (2013). Variability and homogeneity of cardiovascular magnetic resonance myocardial T2-mapping in volunteers compared to patients with edema. J Cardiovasc Magn Reson.

[156] Pennell DJ (2008). T2* magnetic resonance: iron and gold. JACC Cardiovasc Imaging.

[157] Anderson LJ, Holden S, Davis B, Prescott E, Charrier CC, Bunce NH, Firmin DN, Wonke B, Porter J, Walker JM, Pennell DJ (2001). Cardiovascular T2-star (T2*) magnetic resonance for the early diagnosis of myocardial iron overload. Eur Heart J.

[158] Pennell DJ, Udelson JE, Arai AE, Bozkurt B, Cohen AR, Galanello R, Hoffman TM, Kiernan MS, Lerakis S, Piga A (2013). Cardiovascular function and treatment in beta-thalassemia major: a consensus statement from the American Heart Association. Circulation.

[159] Wood JC, Ghugre N (2008). Magnetic resonance imaging assessment of excess iron in thalassemia, sickle cell disease and other iron overload diseases. Hemoglobin.

[160] Carpenter JP, He T, Kirk P, Roughton M, Anderson LJ, de Noronha SV, Sheppard MN, Porter JB, Walker JM, Wood JC (2011). On T2* magnetic resonance and cardiac iron. Circulation.

[161] Kirk P, Smith GC, Roughton M, He T, Pennell DJ (2010). Myocardial T2* is not affected by ageing, myocardial fibrosis, or impaired left ventricular function. J Magn Reson Imaging.

[162] Kirk P, Roughton M, Porter JB, Walker JM, Tanner MA, Patel J, Wu D, Taylor J, Westwood MA, Anderson LJ, Pennell DJ (2009). Cardiac T2* magnetic resonance for prediction of cardiac complications in thalassemia major. Circulation.

[163] Arts T, Prinzen FW, Delhaas T, Milles JR, Rossi AC, Clarysse P (2010). Mapping displacement and deformation of the heart with local sine-wave modeling. IEEE Trans Med Imaging.

[164] Cupps BP, Taggar AK, Reynolds LM, Lawton JS, Pasque MK (2010). Regional myocardial contractile function: multiparametric strain mapping. Interact Cardiovasc Thorac Surg.

[165] Del-Canto I, Lopez-Lereu MP, Monmeneu JV, Croisille P, Clarysse P, Chorro FJ, Bodi V, Moratal D (2015). Characterization of normal regional myocardial function by MRI cardiac tagging. J Magn Reson Imaging.

[166] el Ibrahim SH (2011). Myocardial tagging by cardiovascular magnetic resonance: evolution of techniques–pulse sequences, analysis algorithms, and applications. J Cardiovasc Magn Reson.

[167] Schuster A, Hor KN, Kowallick JT, Beerbaum P, Kutty S (2016). Cardiovascular magnetic resonance myocardial feature tracking: concepts and clinical applications. Circ Cardiovasc Imaging.

[168] Petitjean C, Rougon N, Cluzel P (2005). Assessment of myocardial function: a review of quantification methods and results using tagged MRI. J Cardiovasc Magn Reson.

[169] Miller CA, Borg A, Clark D, Steadman CD, McCann GP, Clarysse P, Croisille P, Schmitt M (2013). Comparison of local sine wave modeling with harmonic phase analysis for the assessment of myocardial strain. J Magn Reson Imaging.

[170] Bogaert J, Rademakers FE (2001). Regional nonuniformity of normal adult human left ventricle. Am J Physiol Heart Circ Physiol.

[171] Jeung MY, Germain P, Croisille P (2012). Myocardial tagging with MR imaging: overview of normal and pathologic findings. Radiographics.

[172] Piella G, De Craene M, Bijnens BH, Tobon-Gomez C, Huguet M, Avegliano G, Frangi AF (2010). Characterizing myocardial deformation in patients with left ventricular hypertrophy of different etiologies using the strain distribution obtained by magnetic resonance imaging. Rev Esp Cardiol.

[173] Castillo E, Osman NF, Rosen BD, El-Shehaby I, Pan L, Jerosch-Herold M, Lai S, Bluemke DA, Lima JA (2005). Quantitative assessment of regional myocardial function with MR-tagging in a multi-center study: interobserver and intraobserver agreement of fast strain analysis with Harmonic Phase (HARP) MRI. J Cardiovasc Magn Reson.

[174] Moore CC, Lugo-Olivieri CH, McVeigh ER, Zerhouni EA (2000). Three-dimensional systolic strain patterns in the normal human left ventricle: characterization with tagged MR imaging. Radiology.

[175] Claus P, Omar AMS, Pedrizzetti G, Sengupta PP, Nagel E (2015). Tissue tracking technology for assessing cardiac mechanics: principles, normal values, and clinical applications. JACC Cardiovasc Imaging.

[176] Peng J, Zhao X, Zhao L, Fan Z, Wang Z, Chen H, Leng S, Allen J, Tan RS, Koh AS (2018). Normal values of myocardial deformation assessed by cardiovascular magnetic resonance feature tracking in a healthy Chinese population: a multicenter study. Front Physiol.

[177] Augustine D, Lewandowski AJ, Lazdam M, Rai A, Francis J, Myerson S, Noble A, Becher H, Neubauer S, Petersen SE, Leeson P (2013). Global and regional left ventricular myocardial deformation measures by magnetic resonance feature tracking in healthy volunteers: comparison with tagging and relevance of gender. J Cardiovasc Magn Reson.

[178] Oxenham HC, Young AA, Cowan BR, Gentles TL, Occleshaw CJ, Fonseca CG, Doughty RN, Sharpe N (2003). Age-related changes in myocardial relaxation using three-dimensional tagged magnetic resonance imaging. J Cardiovasc Magn Reson.

[179] Andre F, Steen H, Matheis P, Westkott M, Breuninger K, Sander Y, Kammerer R, Galuschky C, Giannitsis E, Korosoglou G (2015). Age- and gender-related normal left ventricular deformation assessed by cardiovascular magnetic resonance feature tracking. J Cardiovasc Magn Reson.

[180] Lawton JS, Cupps BP, Knutsen AK, Ma N, Brady BD, Reynolds LM, Pasque MK (2011). Magnetic resonance imaging detects significant sex differences in human myocardial strain. Biomed Eng Online.

[181] Shehata ML, Cheng S, Osman NF, Bluemke DA, Lima JA (2009). Myocardial tissue tagging with cardiovascular magnetic resonance. J Cardiovasc Magn Reson.

[182] Neizel M, Lossnitzer D, Korosoglou G, Schaufele T, Lewien A, Steen H, Katus HA, Osman NF, Giannitsis E (2009). Strain-encoded (SENC) magnetic resonance imaging to evaluate regional heterogeneity of myocardial strain in healthy volunteers: Comparison with conventional tagging. J Magn Reson Imaging.

[183] Venkatesh BA, Donekal S, Yoneyama K, Wu C, Fernandes VR, Rosen BD, Shehata ML, McClelland R, Bluemke DA, Lima JA (2015). Regional myocardial functional patterns: quantitative tagged magnetic resonance imaging in an adult population free of cardiovascular risk factors: the multi-ethnic study of atherosclerosis (MESA). J Magn Reson Imaging.

[184] Cernicanu A, Axel L (2006). Theory-based signal calibration with single-point T1 measurements for first-pass quantitative perfusion MRI studies. Acad Radiol.

[185] Gatehouse PD, Elkington AG, Ablitt NA, Yang GZ, Pennell DJ, Firmin DN (2004). Accurate assessment of the arterial input function during high-dose myocardial perfusion cardiovascular magnetic resonance. J Magn Reson Imaging.

[186] Christian TF, Rettmann DW, Aletras AH, Liao SL, Taylor JL, Balaban RS, Arai AE (2004). Absolute myocardial perfusion in canines measured by using dual-bolus first-pass MR imaging. Radiology.

[187] Ishida M, Schuster A, Morton G, Chiribiri A, Hussain S, Paul M, Merkle N, Steen H, Lossnitzer D, Schnackenburg B (2011). Development of a universal dual-bolus injection scheme for the quantitative assessment of myocardial perfusion cardiovascular magnetic resonance. J Cardiovasc Magn Reson.

[188] Vasu S, Bandettini WP, Hsu LY, Kellman P, Leung S, Mancini C, Shanbhag SM, Wilson J, Booker OJ, Arai AE (2013). Regadenoson and adenosine are equivalent vasodilators and are superior than dipyridamole—a study of first pass quantitative perfusion cardiovascular magnetic resonance. J Cardiovasc Magn Reson.

[189] Fairbairn TA, Motwani M, Mather AN, Biglands JD, Larghat AM, Radjenovic A, Greenwood JP, Plein S (2014). Cardiac MR imaging to measure myocardial blood flow response to the cold pressor test in healthy smokers and nonsmokers. Radiology.

[190] Weng AM, Wilimsky S, Bender G, Hahner S, Kostler H, Ritter CO (2018). Magnetic resonance cold pressor test to investigate potential endothelial dysfunction in patients suffering from type 1 diabetes. J Magn Reson Imaging.

[191] Jerosch-Herold M, Seethamraju RT, Swingen CM, Wilke NM, Stillman AE (2004). Analysis of myocardial perfusion MRI. J Magn Reson Imaging.

[192] Wang L, Jerosch-Herold M, Jacobs DR, Shahar E, Folsom AR (2006). Coronary risk factors and myocardial perfusion in asymptomatic adults: the Multi-Ethnic Study of Atherosclerosis (MESA). J Am Coll Cardiol.

[193] Chareonthaitawee P, Kaufmann PA, Rimoldi O, Camici PG (2001). Heterogeneity of resting and hyperemic myocardial blood flow in healthy humans. Cardiovasc Res.

[194] Moro PJ, Flavian A, Jacquier A, Kober F, Quilici J, Gaborit B, Bonnet JL, Moulin G, Cozzone PJ, Bernard M (2011). Gender differences in response to cold pressor test assessed with velocity-encoded cardiovascular magnetic resonance of the coronary sinus. J Cardiovasc Magn Reson.

[195] Brown LAE, Onciul SC, Broadbent DA, Johnson K, Fent GJ, Foley JRJ, Garg P, Chew PG, Knott K, Dall'Armellina E (2018). Fully automated, inline quantification of myocardial blood flow with cardiovascular magnetic resonance: repeatability of measurements in healthy subjects. J Cardiovasc Magn Reson.

[196] Madriago E, Wells R, Sahn DJ, Diggs BS, Langley SM, Woodward DJ, Jerosch-Herold M, Silberbach M (2015). Abnormal myocardial blood flow in children with mild/moderate aortic stenosis. Cardiol Young.

[197] Bhuva AN, Bai W, Lau C, Ye Y, Bulluck H, McAlindon E, Culotta V, Swoboda PP, Captur G (2019). A multicenter, scan-rescan, human and machine learning CMR study to test generalizability and precision in imaging biomarker analysis. Circ Cardiovasc Imaging.

[198] Bernard O, Lalande A, Zotti C, Cervenansky F, Yang X, Heng PA, Cetin I, Lekadir K, Camara O, Gonzalez Ballester MA (2018). Deep learning techniques for automatic MRI cardiac multi-structures segmentation and diagnosis: is the problem solved?. IEEE Trans Med Imaging.

[199] Curiale AH, Colavecchia FD, Mato G (2019). Automatic quantification of the LV function and mass: a deep learning approach for cardiovascular MRI. Comput Methods Programs Biomed.

[200] Khened M, Kollerathu VA, Krishnamurthi G (2019). Fully convolutional multi-scale residual DenseNets for cardiac segmentation and automated cardiac diagnosis using ensemble of classifiers. Med Image Anal.

[201] Tan LK, McLaughlin RA, Lim E, Abdul Aziz YF, Liew YM (2018). Fully automated segmentation of the left ventricle in cine cardiac MRI using neural network regression. J Magn Reson Imaging.

[202] Tong Q, Li C, Si W, Liao X, Tong Y, Yuan Z, Heng PA (2019). RIANet: Recurrent interleaved attention network for cardiac MRI segmentation. Comput Biol Med.

[203] Tran PV. A fully convolutional neural network for cardiac segmentation in short-axis MRI. arXiv preprint. 2016.

[204] Vigneault DM, Xie W, Ho CY, Bluemke DA, Noble JA (2018). Omega-Net (Omega-Net): fully automatic, multi-view cardiac MR detection, orientation, and segmentation with deep neural networks. Med Image Anal.

[205] Campello VM, Martín-Isla C, Izquierdo C, Petersen SE, Ballester MAG, Lekadir K. Combining multi-sequence and synthetic images for improved segmentation of late gadolinium enhancement cardiac MRI. arXiv preprint. 2019.

[206] Moccia S, Banali R, Martini C, Muscogiuri G, Pontone G, Pepi M, Caiani EG (2019). Development and testing of a deep learning-based strategy for scar segmentation on CMR-LGE images. MAGMA.

[207] Fahmy AS, El-Rewaidy H, Nezafat M, Nakamori S, Nezafat R (2019). Automated analysis of cardiovascular magnetic resonance myocardial native T1 mapping images using fully convolutional neural networks. J Cardiovasc Magn Reson.

[208] Bratt A, Kim J, Pollie M, Beecy AN, Tehrani NH, Codella N, Perez-Johnston R, Palumbo MC, Alakbarli J, Colizza W (2019). Machine learning derived segmentation of phase velocity encoded cardiovascular magnetic resonance for fully automated aortic flow quantification. J Cardiovasc Magn Reson.

[209] Zheng Q, Delingette H, Ayache N (2019). Explainable cardiac pathology classification on cine MRI with motion characterization by semi-supervised learning of apparent flow. Med Image Anal.

[210] Ronneberger O, Fischer P, Brox T. U-net: Convolutional networks for biomedical image segmentation. In: International Conference on Medical image computing and computer-assisted intervention. Springer; 2015. p. 234–241.

[211] Grand challenges–All Challenges. https://grand-challenge.org/challenges/. Accessed 17 Oct 2019.

[212] Backhaus SJ, Staab W, Steinmetz M, Ritter CO, Lotz J, Hasenfuss G, Schuster A, Kowallick JT (2019). Fully automated quantification of biventricular volumes and function in cardiovascular magnetic resonance: applicability to clinical routine settings. J Cardiovasc Magn Reson.

[213] Radau P, Lu Y, Connelly K, Paul G, Dick A, Wright G. Evaluation framework for algorithms segmenting short axis cardiac MRI. In: The MIDAS Journal-Cardiac MR Left Ventricle Segmentation Challenge. 2009. p. 49.

[214] Suinesiaputra A, Cowan BR, Finn JP, Fonseca CG, Kadish AH, Lee DC, Medrano-Gracia P, Warfield SK, Tao W, Young AA. Left ventricular segmentation challenge from cardiac MRI: a collation study. In: International Workshop on Statistical Atlases and Computational Models of the Heart. Springer; 2011. p. 88–97.

[215] Petitjean C, Zuluaga MA, Bai W, Dacher J-N, Grosgeorge D, Caudron J, Ruan S, Ayed IB, Cardoso MJ, Chen H-C (2015). Right ventricle segmentation from cardiac MRI: a collation study. Med Image Anal.

[216] Booz Allen Hamilton Inc., Kaggle, 2015. Second annual data science bowl. https://www.kaggle.com/c/second-annual-data-science-bowl. Accessed 17 Oct 2019.

[217] Fonseca CG, Backhaus M, Bluemke DA, Britten RD, Chung JD, Cowan BR, Dinov ID, Finn JP, Hunter PJ, Kadish AH (2011). The Cardiac Atlas Project—an imaging database for computational modeling and statistical atlases of the heart. Bioinformatics.

