# Correction to: Reference ranges (“normal values”) for cardiovascular magnetic resonance (CMR) in adults and children: 2020 update

**DOI:** 10.1186/s12968-021-00815-3

**Published:** 2021-10-18

**Authors:** Nadine Kawel-Boehm, Scott J. Hetzel, Bharath Ambale-Venkatesh, Gabriella Captur, Christopher J. Francois, Michael Jerosch-Herold, Michael Salerno, Shawn D. Teague, Emanuela Valsangiacomo-Buechel, Rob J. van der Geest, David A. Bluemke

**Affiliations:** 1 Department of Radiology, Kantonsspital Graubuenden, Loestrasse 170, 7000 Chur, Switzerland; 2 Institute for Diagnostic, Interventional and Pediatric Radiology (DIPR), Bern University Hospital, Inselspital, University of Bern, Freiburgstrasse 10, 3010, Bern, Switzerland; 3 Department of Biostatistics and Medical Informatics, University of Wisconsin, 610 Walnut St, Madison, WI 53726 USA; 4 Department of Radiology, Johns Hopkins University, 600 N Wolfe Street, Baltimore, MD 21287 USA; 5 MRC Unit of Lifelong Health and Ageing At UCL, 5-19 Torrington Place, Fitzrovia, London, WC1E 7HB UK; 6 Inherited Heart Muscle Conditions Clinic, Royal Free Hospital NHS Foundation Trust, Hampstead, London, NW3 2QG UK; 7 Department of Radiology, University of Wisconsin School of Medicine and Public Health, 600 Highland Avenue, Madison, WI 53792 USA; 8 Department of Radiology, Brigham and Women’s Hospital, 75 Francis Street, Boston, MA 02115 USA; 9 Cardiovascular Division, University of Virginia Health System, 1215 Lee Street, Charlottesville, VA 22908 USA; 10 Department of Radiology, National Jewish Health, 1400 Jackson St, Denver, CO 80206 USA; 11 Division of Paediatric Cardiology, University Children’s Hospital Zurich, Steinwiesstrasse 75, 8032 Zurich, Switzerland; 12 Department of Radiology, Leiden University Medical Center, Albinusdreef 2, 2333ZA Leiden, The Netherlands

## Correction to: J Cardiovasc Magn Reson (2020) 22: 87 https://doi.org/10.1186/s12968-020-00683-3

Following publication of the original article [1], the authors identified an error in table 39.

The original publication by Davis et al. in JCMR in 2014, which was cited in table 39 of Kawel-Boehm in 2020, presented data in an unusual format (mean ± 2*SD instead of mean ± SD), inducing an error in the calculation of the lower and upper limits of Kawel-Boehm [1].

The incorrect and correct table 39 are shown in this correction article as Tables 1 and 2. The original article has been updated.

**Table.1:** Incorrect table 39

Level	Men (n = 208) Mean ± SD (LL–UL)^a^	Women (n = 239) Mean ± SD (LL–UL)^a^
Ascending aorta diameter (mm)	27 ± 8 (11–42)	26 ± 7 (11–40)
Proximal descending aorta diameter (mm)	21 ± 6 (9–32)	19 ± 4 (11–27)
Distal descending aorta diameter (mm)	18 ± 5 (7–28)	16 ± 4 (8–24)

Measurements obtained on cross-sectional bSSFP images of the aorta

*bSSFP* balanced steady-state free precession, *n* number of study subjects, *SD* standard deviation, *LL* lower limit, *UL* upper limit

^a^Calculated as mean ± 2*SD

**Table.2:** Correct table 39: Normal values of the thoracic aortic luminal diameters for men and women measured at diastole on bSSFP images according to [86]

Level	Men (n = 208) mean ± SD (LL–UL)*	Women (n = 239) mean ± SD (LL–UL)*
Ascending aorta diameter (mm)	27 ± 4 (19–34)	26 ± 4 (18–33)
Proximal descending aorta diameter (mm)	21 ± 3 (15–26)	19 ± 2 (15–23)
Distal descending aorta diameter (mm)	18 ± 3 (13–23)	16 ± 2 (12–20)

Measurements obtained on cross-sectional bSSFP images of the aorta

*bSSFP* balanced steady-state free precession, *n* number of study subjects, *SD* standard deviation, *LL* lower limit, *UL* upper limit

*Calculated as mean ± 2*SD
